# Conformationally Driven Dual Fluorescence Properties of Higher Heteroacenes With Periodically Incorporated Boron Atoms

**DOI:** 10.1002/anie.3365679

**Published:** 2026-03-10

**Authors:** Takeshi Yokochi, Hiromichi Yokoyama, Tetsuyoshi Tsukada, Hayato Sakai, Taku Hasobe, Takanori Fukushima, Yoshiaki Shoji

**Affiliations:** ^1^ Laboratory for Chemistry and Life Science Institute of Integrated Research, Institute of Science Tokyo Yokohama Japan; ^2^ Department of Chemical Science and Engineering School of Materials and Chemical Technology Institute of Science Tokyo Yokohama Japan; ^3^ Faculty of Science and Technology Department of Chemistry Keio University Yokohama Japan; ^4^ Research Center for Autonomous Systems Materialogy (ASMat) Institute of Integrated Research Institute of Science Tokyo Yokohama Japan

**Keywords:** boron, density functional calculations, heterocycles, luminescence, main group elements

## Abstract

Hexaboraheptacene (**B_6_‐hept**), featuring a periodic arrangement of boron atoms, together with its shorter homologues tetraborapentacene (**B_4_‐pent**) and diboraanthracene (**B_2_‐ant**), were synthesized via cyclocondensation of *o*‐bis(dihydroboryl)arenes. The reactive boron centers are effectively protected by 2,4,6‐triisopropylphenyl (Tip) groups, enabling handling under ambient conditions. As the number of boron bridges increases, the electron‐accepting ability as well as the multistep reversible redox properties are progressively enhanced. **B_6_‐hept** exhibits pronounced dual fluorescence in solution, in which relative intensities depend on the excitation wavelength and solvent viscosity. DFT calculations suggest that this phenomenon originates from two interconvertible conformers, namely, the bent‐zigzag and twisted forms, which result from a delicate balance between the extended π‐system and the steric demands of the Tip groups. This interpretation is supported by the fact that **B_4_‐pent** displays only weak dual emission, while **B_2_‐ant** shows none. This study not only introduces a new class of heteroacenes with potential applications in optoelectronics and electrocatalysis, but also provides insight into steric‐design strategies for tuning the conformational landscapes of boron‐containing π‐systems.

## Graphical abstract

We herein report the synthesis of a new heteroheptacene derivative with periodically incorporated six boron atoms. This compound exhibits dual fluorescence attributed to two interconvertible conformers that result from a delicate balance between the extended π‐system and the steric demand of bulky aryl groups attached to the boron centers.

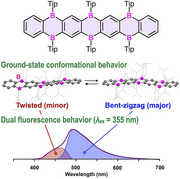

## 1 Introduction

Extending π‐conjugated systems by incorporating three‐coordinate boron atoms is an effective strategy to modulate their chemical and electronic properties. Embedded boron centers lower LUMO energies through p–π* interactions, leading to an enhancement in Lewis acidity and molecular polarization, as well as changes in the frontier orbital patterns [1, 2, 3, 4, 5, 6]. These effects are expected to become even more significant when a larger number of boron atoms are incorporated into a single π‐system. Despite this potential, boron‐rich π‐conjugated molecules remain limited due to challenges in both synthesis and chemical stability. In this context, 9,10‐diboraanthracene [7, 8, 9, 10, 11, 12, 13, 14, 15, 16, 17, 18, 19, 20, 21, 22, 23, 24, 25] has emerged as an attractive building block.

**FIGURE 1:**
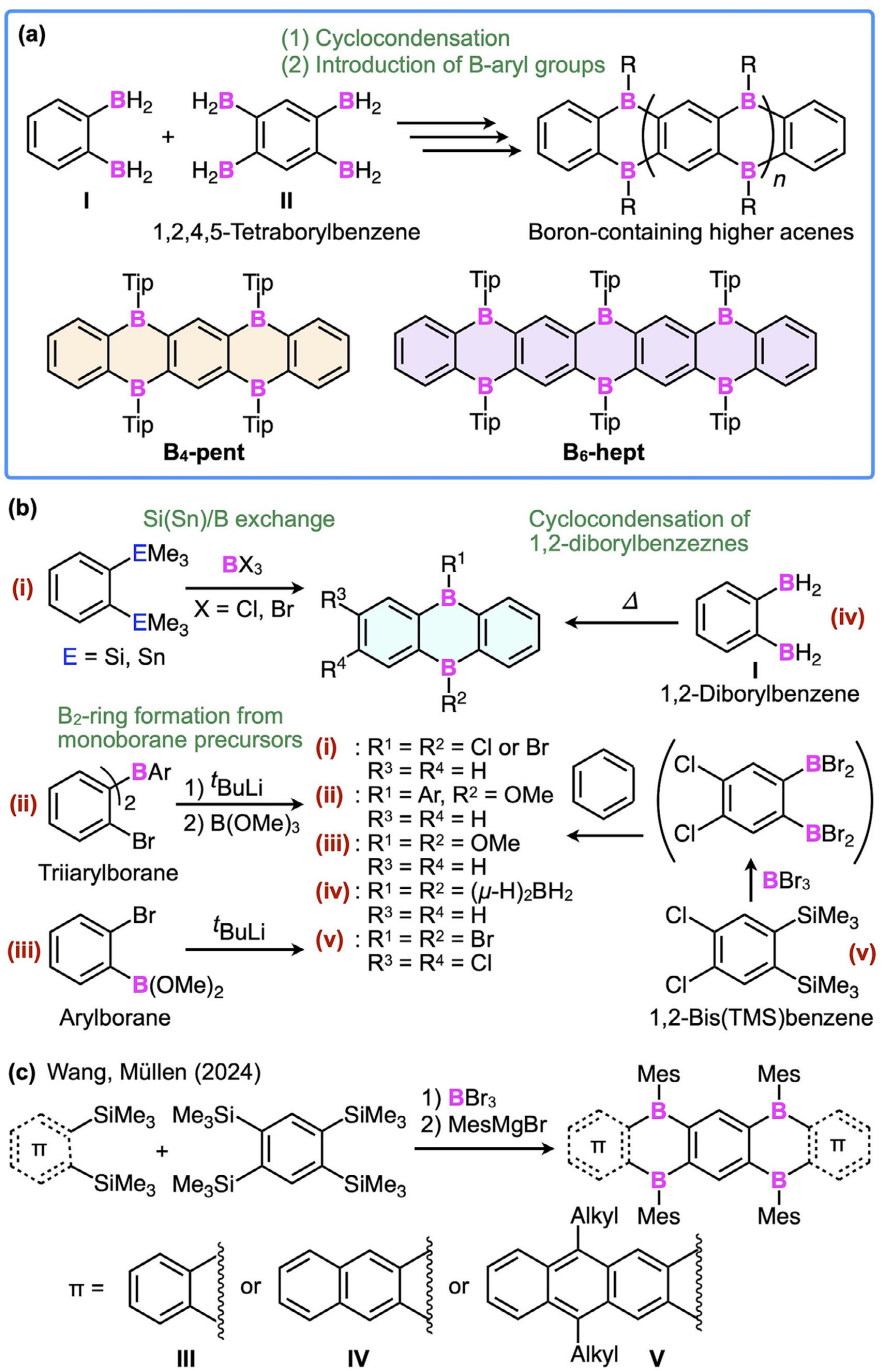
(a) Cyclocondensation of 1,2‐diborylbenzene (**I**) and 1,2,4,5‐tetraborylbenzene (**II**), followed by introduction of aryl groups at the boron centers, affording boron‐incorporating higher acenes **B_4_‐pent** and **B_6_‐hept**. (b) Representative synthetic methods for constructing 9,10‐diboraanthracene skeletons. (c) Reported synthesis of tetraborapentacene (**III**) and its π‐extended analogues (**IV** and **V**) bearing mesityl groups on the boron centers [25].

To date, a broad range of 9,10‐diboraanthracene derivatives have been synthesized and shown to display strong luminescence [7, 8, 9, 10, 11, 12] and multiple reduction behaviors [13], as well as catalytic activity as bidentate Lewis acids [14, 15, 16]. These properties arise from the pair of adjacent electron‐deficient boron centers embedded within the aromatic framework. Motivated by these characteristics, we sought to extend the 9,10‐diboraanthracene motif by linearly connecting multiple benzene rings through boron atoms to access higher homologues (Figure 1a). Such extended π‐systems with periodically incorporated boron atoms may serve as platforms to elucidate how, and to what extent, boron incorporation influences the structure and properties of π‐conjugated systems.

**FIGURE 2:**
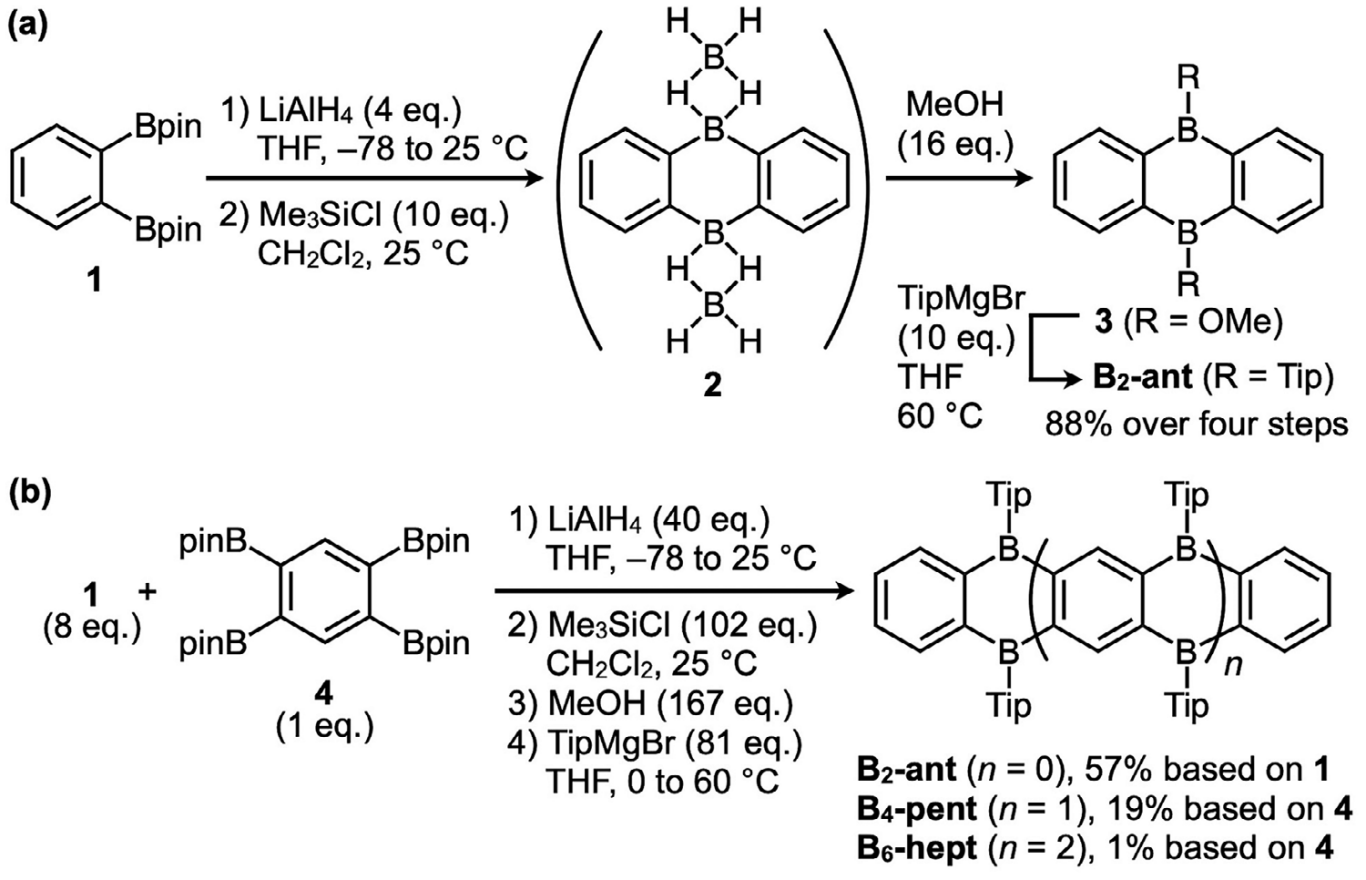
(a) Optimized conditions for the synthesis of **B_2_‐ant** from 1,2‐benzenediboronic acid bis(pinacol) ester (**1**). (b) Synthesis of **B_6_‐hept**, along with **B_4_‐pent** and **B_2_‐ant**, from **1** and 1,2,4,5‐benzenetetraboronic acid tetra(pinacol) ester (**4**).

Here we report the synthesis and properties of a new heptacene derivative (**B_6_‐hept**) in which six boron atoms are embedded at the 5,7,9,14,16, and 18‐positions (Figures 1a and 2). This compound, the most boron‐rich acene reported to date, has been obtained by the cyclocondensation of 1,2‐diborylbenzene (**I**) and 1,2,4,5‐tetraborylbenzene (**II**), which also furnishes its shorter analogues, 5,7,12,14‐tetraborapentacene (**B_4_‐pent**) and 9,10‐diboraanthracene (**B_2_‐ant**). The bulky 2,4,6‐triisopropylphenyl (Tip) groups protect the hydrolyzable boron centers, allowing chromatographic isolation of these boron‐containing acenes. As expected, the multielectron‐accepting ability increases as the π‐system is extended through boron bridges. Unexpectedly, however, extension of the π‐framework also introduces substantial steric congestion from the Tip groups, which distorts the heteroacene backbone and results in a pronounced environment‐dependent dual fluorescence.

## 2 Results and Discussion

Figure 1b summarizes representative synthetic methods for constructing the 9,10‐diboraanthracene skeleton, including (i) Si(Sn)/B exchange [17, 18, 19], (ii, iii) B_2_‐ring formation from monoborane precursors [20, 21], (iv) spontaneous cyclocondensation of *o*‐bis(dihydroboryl)benzene [22, 23], and (v) direct fusion of 4,5‐dichloro‐1,2‐bis(dibromoboryl)benzene with arenes [24]. Recently, Wang et al. employed method (i) to synthesize tetraborapentacene **III** and its π‐extended analogues **IV** and **V** (Figure 1c) [25], which bear mesityl groups on the boron centers, using 1,2,4,5‐tetrakis(trimethylsilyl) benzene and the corresponding 1,2‐bis(trimethylsilyl)arene as starting materials. However, the synthesis of higher acenes incorporating more than four boron atoms has thus far been unsuccessful due to steric hindrance effects along with the instability of reaction intermediates.

To achieve further structural extension via boron bridges, we focused on method (iv), originally developed by Wagner and co‐workers, due to its operational simplicity [22, 23]. We optimized the reaction conditions in terms of the solvent, reagent stoichiometry, reaction temperature, and reaction time to enable the efficient synthesis of **B_2_‐ant**. In addition, to improve the overall yield of the final heteroacene products, we developed a protocol in which the four‐step sequence from compound **1** to **B_2_‐ant** is carried out without isolating the synthetic intermediates. This synthetic protocol can be readily adapted to access higher heteroacenes (Figure 1a). Figure 2a outlines the optimized four‐step synthesis of **B_2_‐ant** starting from **1** [26]. The initial step involves hydride reduction of **1** to generate **I**, which then undergoes spontaneous cyclocondensation to produce diboraanthracene **2** containing two sets of exocyclic B(*μ*‐H)_2_B three‐center two‐electron bonds [22, 23]. Subsequent treatment of **2** with dry methanol affords methoxy‐substituted derivative **3**. Finally, **B_2_‐ant** is obtained by reacting **3** with 2,4,6‐triisopropylphenylmagnesium bromide (TipMgBr). Notably, this procedure does not require the isolation of intermediates and furnishes **B_2_‐ant** in a satisfactory high overall yield (88% from **1**).

We applied these optimized conditions to the synthesis of higher acenes using **1** and 1,2,4,5‐benzenetetraboronic acid tetra(pinacol) ester **4** as the starting materials [26]. The crude mixture obtained from the three‐step procedure (Figure 2b) was subjected to size‐exclusion chromatography (see Figure S1 for details), which enabled the isolation of **B_6_‐hept** (yellow powder, 1% yield from **4**), **B_4_‐pent** (pale yellow powder, 19% yield from **4**), and **B_2_‐ant** (colorless powder, 57% yield from **1**). All products were unambiguously characterized by ^1^H and ^13^C NMR spectroscopy, IR spectroscopy, and high‐resolution APCI‐TOF mass spectrometry (Figures S2–S7 and S15–S26).

The NMR spectrum of **B_6_‐hept** recorded in CDCl_3_ at 298 K exhibits a *C*_2_‐symmetric pattern (Figures S2 and S3). For instance, the singlet at 8.01 ppm is assigned to the aromatic protons adjacent to the central boron bridges, whereas two doublets of doublets at 7.65 and 7.39 ppm correspond to the aromatic protons on the terminal phenylene units. The two aromatic protons of the Tip groups appear as singlets at 6.84 and 6.71 ppm. **B_4_‐pent** and **B_2_‐ant** also show *C*_2_‐symmetric NMR spectral patterns (Figures S4–S7).

**FIGURE 3:**
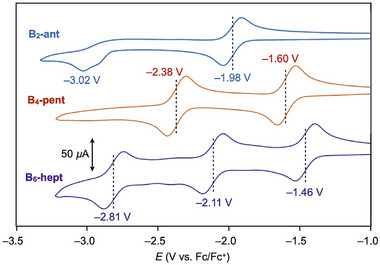
Cyclic voltammograms of **B_2_‐ant** (blue), **B_4_‐pent** (orange), and **B_6_‐hept** (purple) in THF (0.5 mM) containing lithium bis(trifluoromethanesulfonyl)imide (0.1 M) as the supporting electrolyte, recorded at a scan rate of 100 mV s^−1^ at 25°C.

Cyclic voltammetry (CV) measurements revealed that structural extension via boron bridges enhances the electron‐accepting ability. In THF at 25°C, **B_6_‐hept** exhibits three reversible reduction waves at *E*_1/2_ = −1.46, −2.11, and −2.81 V versus the ferrocene/ferrocenium redox couple (Figure 3). In contrast, **B_4_‐pent** shows only two reversible reductions within the electrochemical window at *E*_1/2_ = −1.60 and −2.38 V, both of which occur at more negative potentials than those of **B_6_‐hept**. Meanwhile, **B_2_‐ant** displays a single reversible reduction at an even more negative half potential of −1.98 V, whereas its second reduction (−3.02 V) is irreversible [27]. These results clearly indicate that the multielectron‐accepting character of the boron‐containing acenes increases with increasing π‐conjugation length.

**FIGURE 4:**
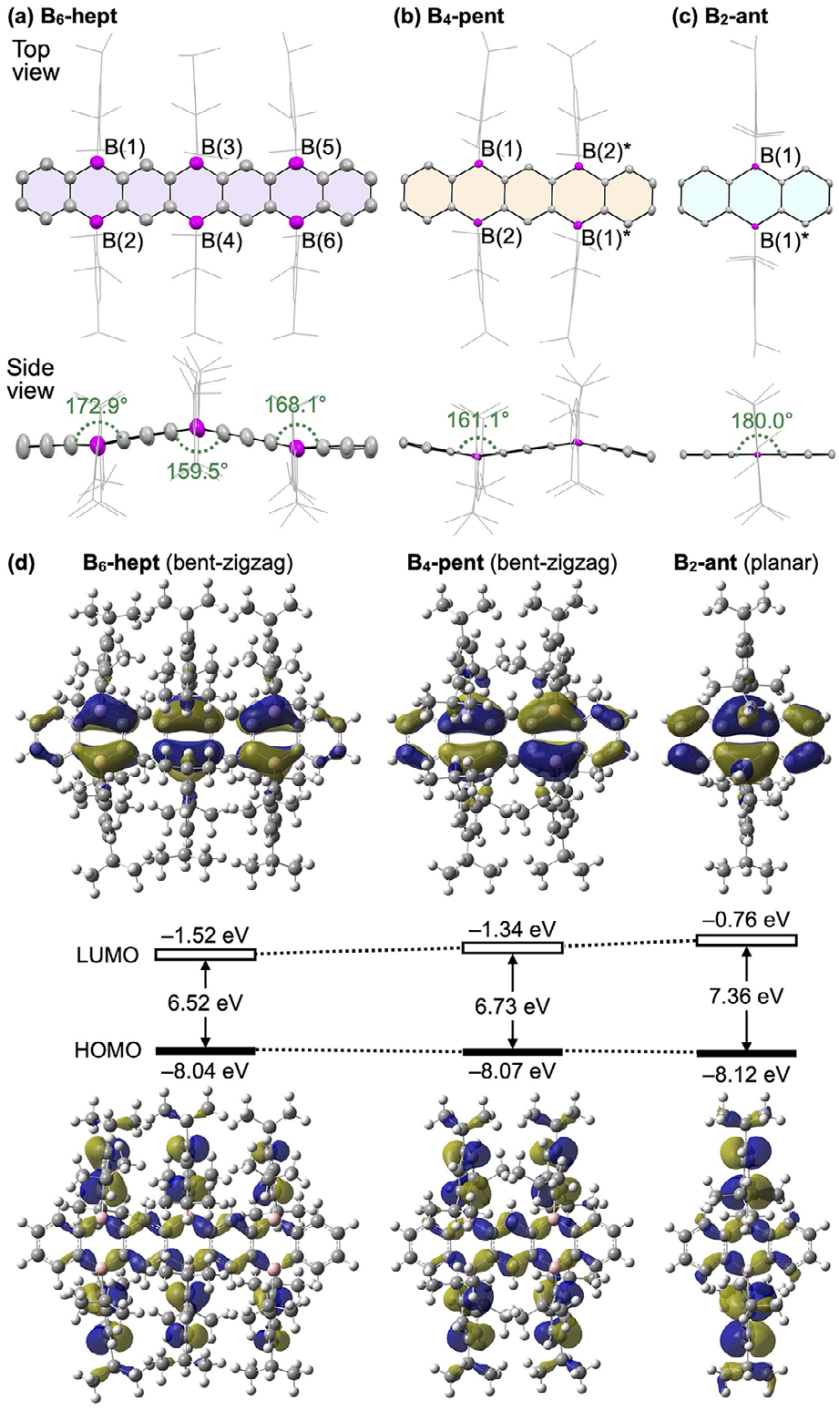
X‐ray crystal structures of (a) **B_6_‐hept**, (b) **B_4_‐pent**, and (c) **B_2_‐ant**. The atomic displacement ellipsoids are drawn at 50% probability. For clarity, the Tip groups are depicted in a line format, and hydrogen atoms are omitted. Color code: boron = pink, carbon = gray. (d) Kohn–Sham orbitals of **B_6_‐hept** (left), **B_4_‐pent** (middle), and **B_2_‐ant** (right), calculated at their respective S_0_‐state equilibrium geometries optimized at the *ω*B97X‐D/6‐311G(d,p)//B3LYP‐D3(BJ)/6‐31G(d) level in vacuum. Energy values are given in eV.

Fortunately, yellow prism‐shaped single crystals of **B_6_‐hept** suitable for x‐ray diffraction analysis were obtained by slow evaporation of its toluene solution [26, 28]. As shown in Figure 4a, **B_6_‐hept** adopts a bent‐zigzag conformation in the crystal. The bending angles at the central boron‐bridged position and the adjacent bridging positions are 159.5° and 172.9/168.1°, respectively. This geometrical distortion is attributed to the bulky Tip groups, which alternate above and below the acene core to minimize intramolecular crowding (Figure 4a, bottom). A similar bent‐zigzag conformation is also observed in the crystal structure of **B_4_‐pent** (Figure 4b). This stands in contrast to the planar molecular structure reported for **III**, which bears less sterically demanding mesityl groups on the boron centers (Figure 1c) [25]. Meanwhile, **B_2_‐ant**, being devoid of significant intramolecular steric repulsion between the Tip groups, adopts a planar conformation in the crystal (Figure 4c).

We were initially concerned that the bending of the acene frameworks in **B_6_‐hept** and **B_4_‐pent** might impair effective p–π* conjugation. However, this concern proved unfounded. In the optimized ground‐state (S_0_) geometries of all heteroacene derivatives, obtained by density functional theory (DFT) calculations under vacuum at the *ω*B97X‐D/6‐311G(d,p)//B3LYP‐D3(BJ)/6‐31G(d) level, the LUMO orbitals are distributed over the entire acene cores (Figure 4d, Tables S2, S3, and S5) [26]. Moreover, the LUMO level clearly decreases with extension of the acene cores (**B_2_‐ant**: −0.76 eV, **B_4_‐pent**: −1.34 eV, and **B_6_‐hept**: −1.52 eV). These results suggest that the observed structural bending has only a limited impact on the overall conjugation. The HOMO level remains nearly unchanged across the homologues (ca. −8.1 eV), reflecting the characteristic electronic influence of the embedded boron centers. Such a selective lowering of the LUMO level upon backbone extension is analogous to the trends reported for monoborane‐bridged linear and cyclic oligomers, where p–π* conjugation with boron plays a key role [10, 11, 12].

**FIGURE 5:**
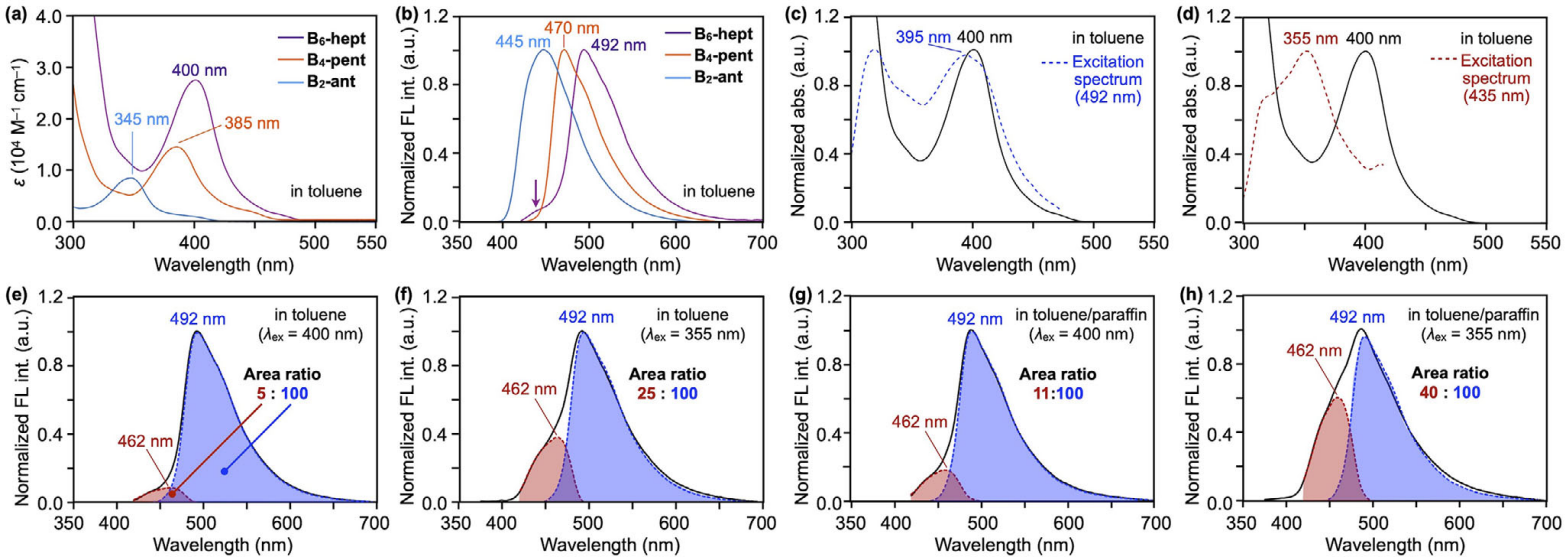
(a) Absorption and (b) fluorescence emission spectra of **B_6_‐hept** (purple), **B_4_‐pent** (orange), and **B_2_‐ant** in toluene. (c) Excitation spectrum of **B_6_‐hept** monitored at 492 nm (blue dotted curve), overlaid with its absorption spectrum (black). (d) Excitation spectrum of **B_6_‐hept** monitored at 435 nm (red dotted curve) overlaid with its absorption spectrum (black). (e,f) Fluorescence spectra of **B_6_‐hept** (black) in toluene upon excitation at (e) 400 nm and (f) 355 nm, with deconvoluted longer‐wavelength (blue) and shorter‐wavelength (red) components. (g,h) Fluorescence spectra of **B_6_‐hept** (black) in toluene/paraffin (1/1 v/v) upon excitation at (g) 400 nm and (h) 355 nm, with deconvoluted longer‐wavelength (blue) and shorter‐wavelength (red) components. All spectra were recorded under air at 298 K in 1.0 × 10^−5^ M solutions.

Figure 5a,b shows the UV‐vis absorption and fluorescence spectra of the boron‐containing acenes in toluene at 25°C (1.0 × 10^−5^ M). As expected, extension of the π‐conjugation leads to bathochromic shifts and enhanced molar absorptivity: *λ*_max_ = 345 nm for **B_2_‐ant**, 385 nm for **B_4_‐pent**, and 400 nm for **B_6_‐hept**. The fluorescence emission spectra, recorded upon excitation at the respective absorption maxima, exhibit progressive red shifts in the order of **B_2_‐ant** (*λ*_em_ = 445 nm, fluorescence quantum yield *Φ*_FL_ = 2.3%), **B_4_‐pent** (*λ*_em_ = 470 nm, *Φ*_FL_ = 2.5%), and **B_6_‐hept** (*λ*_em_ = 492 nm, *Φ*_FL_ = 1.4%). Notably, the fluorescence profile of **B_6_‐hept** differs from those of the other derivatives, displaying a relatively broad, low‐intensity onset centered around 435 nm (purple arrow in Figure 5b) in addition to the main emission peak at 492 nm.

The excitation spectrum of **B_6_‐hept** associated with the 492 nm emission shows a maximum at 395 nm (Figure 5c, blue dotted curve), closely matching the absorption spectrum. In contrast, the excitation spectrum monitored at the 435 nm emission displays a distinctly different profile, lacking the 400 nm peak and instead exhibiting a maximum at 355 nm (Figure 5d, red dotted curve).

Furthermore, when excited at 355 nm, the fluorescence spectrum presents a distinctly different pattern from that observed upon excitation at 400 nm, showing an enhanced relative intensity at 435 nm (Figure 5e,f, black solid curves). These excitation wavelength‐dependent spectra allowed deconvolution into two emissive components, depicted in red and blue in Figure 5e,f. Importantly, the absorption and fluorescence spectral profiles of **B_6_‐hept** remain essentially unchanged upon dilution from 1.0 × 10^−5^ to 1.0 × 10^−6^ M in toluene (Figure S8), ruling out contributions from intermolecular aggregation. Therefore, the dual fluorescence should arise from an intrinsic unimolecular process, most likely conformational isomerism. To verify this notion, we examined the effect of solvent viscosity on the fluorescence behavior, since increased viscosity is expected to suppress intramolecular motions that could influence the emission process [29, 30].

As shown in Figure 5g,h, in a high‐viscosity toluene/paraffin (1/1 v/v) mixture, **B_6_‐hept** exhibits dual fluorescence in which the shorter‐wavelength emission (red) is relatively intense compared to the longer‐wavelength one (blue). Specifically, the area ratio of the red to blue components increases to 11:100 upon 400 nm excitation and to 40:100 upon 355 nm excitation, compared to the corresponding values in toluene (5:100 and 25:100, respectively, Figure 5e,f). In contrast, in a low‐viscosity toluene/*n*‐hexane (1/1 v/v) mixture, the fluorescence spectrum remains essentially identical to that observed in toluene (Figure S9). Importantly, the absorption spectral features of **B_6_‐hept** show no dependence on the viscosity of the medium. Based on the steady‐state spectroscopic results, it is reasonable to conclude that (i) **B_6_‐hept** adopts two distinct conformations in the S_0_ state, (ii) these conformers give rise to two emissive species responsible for the dual fluorescence in the excited singlet (S_1_) state, and (iii) the two emissive species are interconvertible.

**FIGURE 6:**
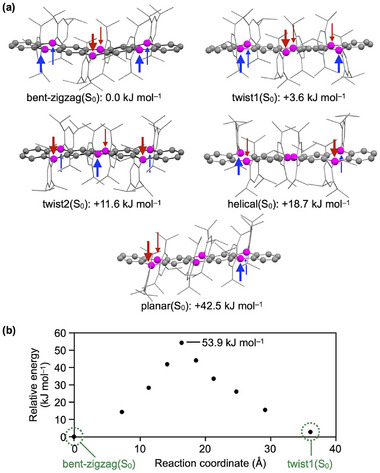
(a) Computed structures and relative Gibbs free energies of five conformers of **B_6_‐hept** obtained from conformational analysis at the *ω*B97X‐D/6‐311G(d,p)//B3LYP‐D3(BJ)/6‐31G(d) level. Blue and red arrows indicate the orientations of the Tip groups with respect to the plane of the B_6_‐heptacene core. (b) Plots of calculated relative total electronic energies (kJ mol^−1^) for bent‐zigzag→twist1 conformational change in the S_0_ state based on NEB calculations performed at the CPCM(toluene)‐B3LYP‐D3(BJ)/6‐31G(d) level. The bent‐zigzag conformer is set as the zero‐energy reference. See also Figure S11 for the highest‐energy structure along the NEB pathway.

To gain insight into the conformational isomerism of **B_6_‐hept**, we performed DFT calculations at the *ω*B97X‐D/6‐311G(d,p)//B3LYP‐D3(BJ)/6‐31G(d) level [26]. We identified five possible equilibrium S_0_ geometries (Figure 6a), consisting of the most stable bent‐zigzag(S_0_) conformer (Figure 4a) and four additional metastable conformers (Tables S5–S9). All conformers are interconvertible through flipping the Tip groups above and below the heptacene core, which modulates the degree of backbone bending and twisting. Among these, twist1(S_0_), in which one terminal Tip group is flipped relative to the bent‐zigzag(S_0_) structure, is the second most stable conformer, lying only 3.6 kJ mol^−1^ higher in energy (Figures 6a and S10, and Table S1). The remaining conformers are much less stable and are therefore expected to contribute negligibly in solution. The activation barrier for the bent‐zigzag(S_0_)→twist1(S_0_) conformational change is estimated to be 53.9 kJ mol^−1^ (Figure 6b) based on nudged elastic band (NEB) calculations [26] performed at the CPCM(toluene)‐B3LYP‐D3(BJ)/6‐31G(d) level (Figure S11, Tables S10 and S11). This barrier indicates that bent‐zigzag(S_0_) and twist1(S_0_) should be in equilibrium at around room temperature. Based on their calculated energy difference, a Boltzmann distribution at 298 K predicts populations of approximately 81% and 19% for bent‐zigzag(S_0_) and twist1(S_0_), respectively. Thus, the major longer‐wavelength (blue) and minor shorter‐wavelength (red) fluorescence components (Figure 5e–h) can be reasonably assigned to excited‐state species originating from bent‐zigzag(S_0_) and twist1(S_0_), hereafter referred to as bent‐zigzag(S_1_) and twist1(S_1_), respectively. Indeed, geometry optimizations of **B_6_‐hept** in the S_1_ state starting from bent‐zigzag(S_0_) and twist1(S_0_) converged to two distinct equilibrium structures that retain the key features of the respective S_0_ geometries (Figure S12, Tables S12 and S13). These results provide additional support for the presence of conformational isomerism in both the ground and excited states.

**FIGURE 7:**
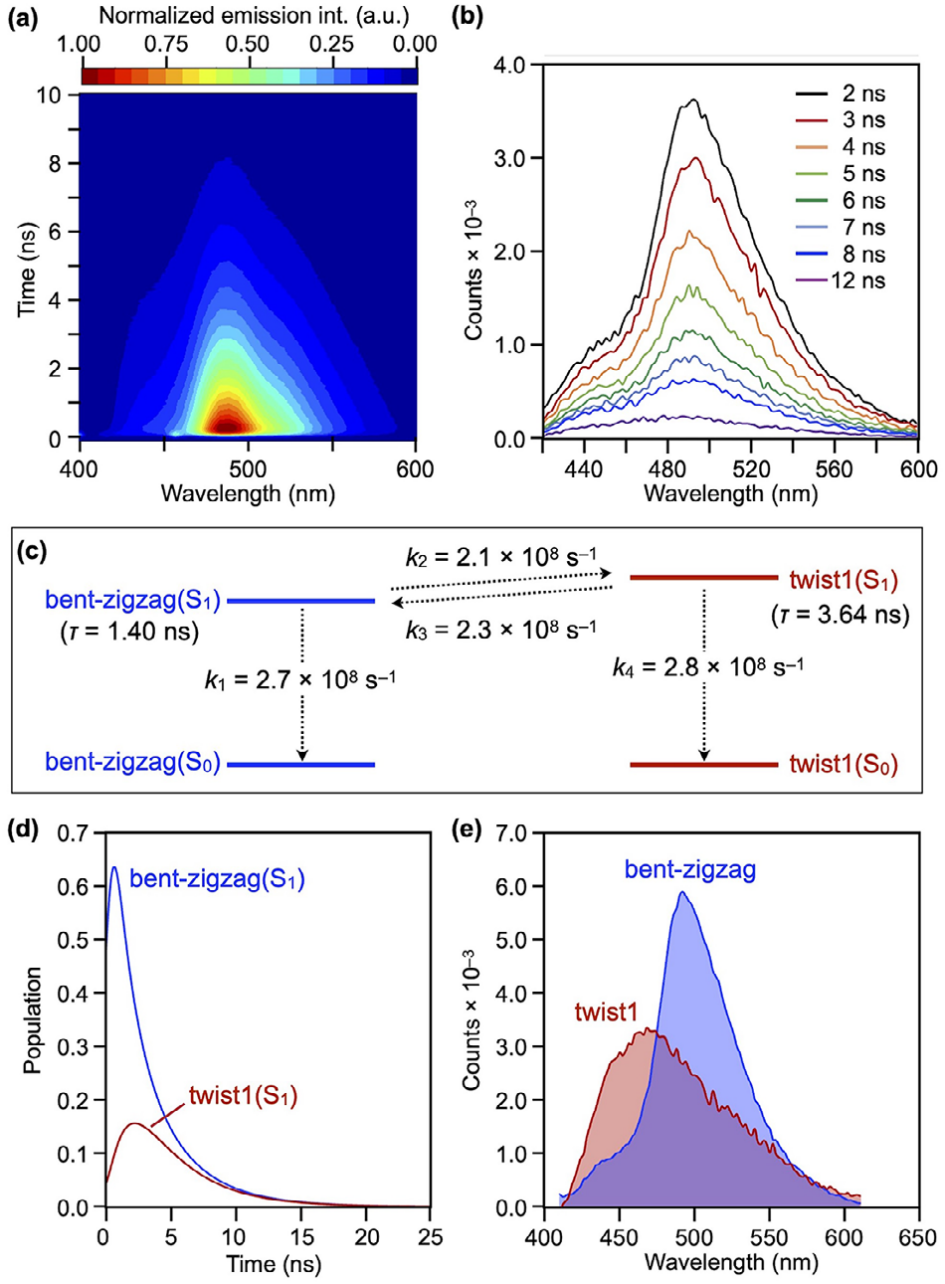
(a) 2D time‐resolved fluorescence spectral profile of **B_6_‐hept** (1.0 × 10^−5^ M) in toluene at 298 K (*λ*_ex_ = 365 nm). (b) Time‐dependent fluorescence spectral change at selected delay time, extracted from panel (a). (c) Summary of the rate constants for the excited‐state processes of **B_6_‐hept** obtained from target analysis. *k*_1_: S_1_→S_0_ decay for bent‐zigzag(_S1_), *k*_2_: bent‐zigzag(S_1_)→twist1(S_1_) conformational change, *k*_3_: twist1(S_1_)→bent‐zigzag(S_1_) conformational change, *k*_4_: S_1_→S_0_ decay for twist1(S_1_). (d) Time course of the populations of bent‐zigzag(S_1_) and twist1(S_1_), calculated using the fitted kinetic parameters. (e) Species‐associated spectra (SAS) obtained from the target analysis, corresponding to the bent‐zigzag (blue) and twist1 (red) conformers.

To further investigate the dual fluorescence behavior of **B_6_‐hept**, we performed time‐resolved fluorescence measurements in toluene at 298 K. The resulting two‐dimensional (2D) spectral profile displays two distinct emission components at around 450 and 500 nm, each with a different lifetime (Figure 7a,b). Target analysis [31] was carried out for these data using a kinetic model (Figure 7c) involving interconversion between bent‐zigzag(S_1_) and twist1(S_1_). The extracted species‐associated spectra (SAS), shown in blue and red in Figure 7e, qualitatively agree with the deconvoluted longer‐ and shorter‐wavelength emission components (Figure 5e–h), which originate from bent‐zigzag(S_1_) and twist1(S_1_), respectively. The time courses of the bent‐zigzag(S_1_) and twist1(S_1_) populations obtained from the target analysis (Figure 7d) reproduce the experimental observation that the relative contribution of the shorter‐wavelength emission component, that is, twist1(S_1_), increases over time while the overall fluorescence intensity decays (Figure 7b). Figure 7c summarizes the obtained rate constants, from which the excited‐state lifetimes (*τ*) were calculated to be 1.40 and 3.64 ns for bent‐zigzag(S_1_) and twist1(S_1_), respectively. The consistency between the experimental and modeled spectral profiles validates the kinetic model established in the target analysis and ensures an accurate description of the conformationally driven dual fluorescence behavior of **B_6_‐hept**.

With the above results in mind, we reinvestigated the steady‐state fluorescence profile of **B_4_‐pent**, focusing on its dependence on excitation wavelength and solvent viscosity (Figure S13). This analysis revealed that **B_4_‐pent** also exhibits dual fluorescence emission, with maxima at 450 and 470 nm, the former being relatively weak. The area ratio of the minor (450 nm) to the major (470 nm) emission component does not exceed 5:100 under the examined conditions (Figure S13). As in the case of **B_6_‐hept**, DFT calculations for **B_4_‐pent** identified the most stable bent‐zigzag (Figure 4b and Table S3) and a higher‐energy twisted conformer (Figure S14 and Table S4), which are considered to give rise to the longer‐ and shorter‐wavelength emissions, respectively. However, the larger energy gap (7.2 kJ mol^−1^) between these two conformers limits the population of the twisted conformer to a minor level, accounting for the much smaller fraction of shorter‐wavelength emission compared with **B_6_‐hept**. As expected, **B_2_‐ant**, which features a rigid and planar geometry, shows no detectable dual fluorescence. Accordingly, conformational diversity and the resulting dual fluorescence properties become more pronounced in the longer π‐extended boron‐containing acenes. This trend can be attributed to the relative stabilization of the metastable conformers in the extended systems, facilitated by increased backbone flexibility and enhanced intramolecular interactions between the bulky Tip groups.

## 3 Conclusion

We have described the synthesis and properties of an unprecedented hexaboraheptacene (**B_6_‐hept**), along with those of its shorter homologues, tetraborapentacene (**B_4_‐pent**) and diboraanthracene (**B_2_‐ant**). These heteroacenes were obtained through cyclocondensation of *o*‐bis(dihydroboryl)arenes. Structural, electrochemical, and theoretical investigations demonstrate that π‐extension via boron bridges progressively enhances the multielectron‐accepting ability. Remarkably, **B_6_‐hept** exhibits dual fluorescence behavior in solution, originating from the most stable bent‐zigzag and metastable twisted conformers that coexist in thermodynamic equilibrium. This behavior reflects the delicate balance between π‐conjugation and steric effects imposed by the bulky aryl substituents at the boron centers. In contrast, **B_4_‐pent** displays only weak dual fluorescence and **B_2_‐ant** none, consistent with their more limited conformational flexibility. These findings establish steric and conformational design as an effective approach to modulating emission properties in boron‐containing π‐systems, offering new opportunities for the development of responsive functional materials in optoelectronics and photocatalysis.

## Conflicts of Interest

The authors declare no conflicts of interest.

## Supporting information




**Supporting File 1**: anie71760‐sup‐0001‐SuppMat.pdf.

## Data Availability

The data that supports the findings of this study are available in the Supporting Information of this article.

## References

[1] S. Yamaguchi , S. Akiyama , and K. Tamao , “Colorimetric Fluoride Ion Sensing by Boron‐Containing π‐Electron Systems,” Journal of the American Chemical Society 123 (2001): 11372–11375, 10.1021/ja015957w. 11707112

[2] Z. Zhou , A. Wakamiya , T. Kushida , and S. Yamaguchi , “Planarized Triarylboranes: Stabilization by Structural Constraint and Their Plane‐to‐Bowl Conversion,” Journal of the American Chemical Society 134 (2012): 4529–4532, 10.1021/ja211944q. 22369126

[3] C. Dou , S. Saito , K. Matsuo , I. Hisaki , and S. Yamaguchi , “A Boron‐Containing PAH as a Substructure of Boron‐Doped Graphene,” Angewandte Chemie International Edition 124 (2012): 12206–12210; 10.1002/anie.201206699 23081889

[4] T. Hatakeyama , K. Shiren , K. Nakajima , et al., “Ultrapure Blue Thermally Activated Delayed Fluorescence Molecules: Efficient HOMO–LUMO Separation by the Multiple Resonance Effect,” Advanced Materials 28 (2016): 2777–2781, 10.1002/adma.201505491. 26865384

[5] S. Kawai , S. Saito , S. Osumi , et al., “Atomically Controlled Substitutional Boron‐Doping of Graphene Nanoribbons,” Nature Communications 6 (2015): 8098, 10.1038/ncomms9098. PMC4560828 26302943

[6] R. R. Cloke , T. Marangoni , G. D. Nguyen , et al., “Site‐Specific Substitutional Boron Doping of Semiconducting Armchair Graphene Nanoribbons,” Journal of the American Chemical Society 137 (2015): 8872–8875, 10.1021/jacs.5b02523. 26153349

[7] A. Lorbach , M. Bolte , H. Li , et al., “9,10‐Dihydro‐9,10‐diboraanthracene: Supramolecular Structure and Use as a Building Block for Luminescent Conjugated Polymers,” Angewandte Chemie International Edition 121 (2009): 4584–4588; 10.1002/anie.200901226 19455524

[8] C. Reus , S. Weidlich , M. Bolte , H.‐W. Lerner , and M. Wagner , “C‐Functionalized, Air‐ and Water‐Stable 9,10‐Dihydro‐9,10‐diboraanthracenes: Efficient Blue to Red Emitting Luminophores,” Journal of the American Chemical Society 135 (2013): 12892–12907, 10.1021/ja406766e. 23899377

[9] T.‐L. Wu , C.‐H. Yeh , W.‐T. Hsiao , et al., “High‐Performance Organic Light‐Emitting Diode With Substitutionally Boron‐Doped Graphene Anode,” ACS Applied Materials & Interfaces 9 (2017): 14998–15004, 10.1021/acsami.7b03597. 28385015

[10] P. Chen , R. A. Lalancette , and F. Jäkle , “Applying the Oligomer Approach to Luminescent Conjugated Organoboranes,” Journal of the American Chemical Society 133 (2011): 8802–8805, 10.1021/ja201436a. 21591640

[11] N. Baser‐Kirazli , R. A. Lalancette , and F. Jäkle , “Enhancing the Acceptor Character of Conjugated Organoborane Macrocycles: A Highly Electron‐Deficient Hexaboracyclophane,” Angewandte Chemie International Edition 132 (2020): 8689–8697; 10.1002/anie.202001904 32129932

[12] X. Yin , J. Liu , and F. Jäkle , “Electron‐Deficient Conjugated Materials via p–π* Conjugation With Boron: Extending Monomers to Oligomers, Macrocycles, and Polymers,” Chemistry A–European Journal 27 (2021): 2973–2986, 10.1002/chem.202003481. 32852793

[13] P. Müller , S. Huck , H. Köppel , H. Pritzkow , and W. Siebert , “Synthesis and Structures of 9,10‐Dihydro‐9,10‐Diboraanthracene Derivatives,” Z Naturforsch 50b (1995): 1476–1484, 10.1515/znb-1995-1008.

[14] S. N. Kessler and H. A. Wegner , “Lewis Acid Catalyzed Inverse Electron‐Demand Diels−Alder Reaction of 1,2‐Diazines,” Organic Letters 12 (2010): 4062–4065, 10.1021/ol101701z. 20718452

[15] M. L. Landry , G. M. McKenna , and N. Z. Burns , “Enantioselective Synthesis of Azamerone,” Journal of the American Chemical Society 141 (2019): 2867–2871, 10.1021/jacs.8b12566. 30707836 PMC6497086

[16] M. Balkenhohl , H. Jangra , T. Lenz , et al., “Lewis‐Säure‐dirigierte regioselektive Metallierungen an Pyridazin,” Angewandte Chemie International Edition 131 (2019): 9244–9247; 10.1002/anie.201903839 31034125

[17] W. Schacht and D. Kaufmann , “Thermolyse von Arylhalogenboranen; Synthese von 1,3‐Dibora‐ und 1,3‐Borasilaindanen,” Journal of Organometallic Chemistry 331 (1987): 139–152, 10.1016/0022-328X(87)80015-3.

[18] M. V. Metz , D. J. Schwartz , C. L. Stern , P. N. Nickias , and T. J. Marks , “Organo‐Lewis Acid Cocatalysts in Single‐Site Olefin Polymerization—A Highly Acidic Perfluorodiboraanthracene,” Angewandte Chemie International Edition 112 (2000):1312–1316; 10.1002/(sici)1521-3773(20000403)39:7<1312::aid-anie1312>3.0.co;2-4 10767042

[19] J. Chen , J. W. Kampf , and A. J. Ashe III , “Syntheses and Structures of 6,13‐Dihydro‐6,13‐diborapentacenes: Π‐Stacking in Heterocyclic Analogues of Pentacene,” Organometallics 27 (2008): 3639–3641, 10.1021/om8005068.

[20] T. Agou , M. Sekine , and T. Kawashima , “Stepwise Synthesis and Properties of a 9,10‐Dihydro‐9,10‐diboraanthracene,” Tetrahedron Letters 51 (2010): 5013–5015, 10.1016/j.tetlet.2010.07.068.

[21] S. Luliński , J. Smętek , K. Durka , and J. Serwatowski , “Tandem Synthesis of 9,10‐Dihydro‐9,10‐diboraanthracenes via Elusive ortho‐Lithiated Phenylboronates,” European Journal of Organic Chemistry (2013): 8315–8322, 10.1002/ejoc.201300868.

[22] Ö. Seven , Z.‐W. Qu , H. Zhu , et al., “Synthesis, Coupling, and Condensation Reactions of 1,2‐Diborylated Benzenes: An Experimental and Quantum‐Chemical Study,” Chemistry A–European Journal 18 (2012): 11284–11295, 10.1002/chem.201201547. 22847999

[23] A. John , S. Kirschner , M. K. Fengel , M. Bolte , H.‐W. Lerner , and M. Wagner , “Simultaneous Expansion of 9,10 Boron‐Doped Anthracene in Longitudinal and Lateral Directions,” Dalton Transactions 48 (2019): 1871–1877, 10.1039/C8DT04820G. 30608493

[24] A. John , M. Bolte , H.‐W. Lerner , and M. Wagner , “A Vicinal Electrophilic Diborylation Reaction Furnishes Doubly Boron‐Doped Polycyclic Aromatic Hydrocarbons,” Angewandte Chemie International Edition 129 (2017): 5588–5592; 10.1002/anie.201701591 28402016

[25] C. Chen , Y. Guo , Z. Chang , K. Müllen , and X.‐Y. Wang , “Synthesis of Quadruply Boron‐Doped Acenes With Stimuli‐Responsive Multicolor Emission,” Nature Communications 15 (2024): 8555, 10.1038/s41467-024-51806-8. PMC11450196 39362864

[26] See the Supporting Information for Experimental and Computational Details.

[27] In Contrast to the Irreversible Second Reduction Observed for **B_2_‐ant**, a Diboraanthracene Derivative Bearing Mesityl Groups Instead of Tip on the Boron Atoms has been Reported to Exhibit two Reversible Reduction Waves Under Similar Cyclic Voltammetry Conditions (ref. [22]).

[28] Deposition Numbers 2517732 (for **B_6_‐hept**), 2517731 (for **B_4_‐pent**), and 2517730 (for **B_2_‐ant**) Contain the Supplementary Crystallographic Data for This Paper. These Data are Provided Free of Charge by the Joint Cambridge Crystallographic Data Centre and Fachinformationszentrum Karlsruhe Access Structures Service, www.ccdc.cam.ac.uk/structures.

[29] S. Sasaki , G. P. C. Drummen , and G. Konishi , “Recent Advances in Twisted Intramolecular Charge Transfer (TICT) Fluorescence and Related Phenomena in Materials Chemistry,” Journal of Materials Chemistry C 4 (2016): 2731–2743, 10.1039/C5TC03933A.

[30] Y. Shoji , N. Tanaka , Y. Ikabata , et al., “Tetraaryldiborane(4) Can Emit Dual Fluorescence Responding to the Structural Change around the B–B Bond,” Angewandte Chemie International Edition 61 (2022): e202113549, 10.1002/anie.202113549; 34677888

[31] J. J. Snellenburg , S. Laptenok , R. Seger , K. M. Mullen , and I. H. M. van Stokkum , “Glotaran: A Java‐Based Graphical User Interface for the R Package TIMP,” Journal of Statistical Software 49 (2012): 1–22, 10.18637/jss.v049.i03.

