# Supplementary material for: Conformationally Driven Dual Fluorescence Properties of Higher Heteroacenes With Periodically Incorporated Boron Atoms
Source: Angew Chem Int Ed Engl. 2026 Mar 10;65(17):e3365679. doi: 10.1002/anie.3365679 (PMC13098299; doi:10.1002/anie.3365679)
Supplement: Supplementary file 1 — Supporting File 1: anie71760‐sup‐0001‐SuppMat.pdf. [file ANIE-65-e3365679-s001.pdf]

Supporting Information for "Conformationally Driven Dual Fluorescence Properties of Higher Heteroacenes with Periodically Incorporated Boron Atoms"

Table of Contents

1. **Materials and Methods** S2
2. **Synthesis** S2
3. **Single-crystal X-ray Diffraction Analysis** S4
4. **Computational Details** S5
5. **Supporting References** S6
6. **Supporting Figures and Table (Figures S1–S14 and Table S1)** S8
7. **Analytical Data (Figures S15–S26)** S20
8. **Optimized Cartesian Coordinates (in Å) and Energies (Tables S2–S13)** S37

1. Materials and Methods

Handling of air- and/or moisture-sensitive compounds were performed either using standard Schlenk-line techniques or in a glove box under argon. Anhydrous hexane, THF, and CH_2Cl_2 were dried by passage through an activated alumina column and a Q-5 column (Nikko Hansen & Co., Ltd.). 1,2-Benzenediboronic acid bis(pinacol)ester[S1] (**1**) and 1,2,4,5-benzenetetraboronic acid tetra(pinacol)ester[S2] (**4**) were prepared according to previously reported procedures.

Infrared (IR) spectra were recorded at 25 °C on a JASCO model FT/IR-660*Plus* Fourier transform infrared spectrometer. Nuclear magnetic resonance (NMR) spectroscopy measurements were carried out on a Bruker model AVANCE-400 spectrometer (400.0 MHz for ^{1}H, ^{13}C: 100.6 MHz) or on a Bruker model AVANCE III HD-500 spectrometer (^{1}H: 500.0 MHz, ^{13}C: 125.7 MHz), where chemical shifts (δ) were determined with respect to residual solvent for ^{1}H (residual non-deuterated $CHCl_3$ in $CDCl_3$: $^{1}H(\delta)$ = 7.26 ppm), and residual solvent for ^{13}C ($CDCl_3$: $^{13}C(\delta)$ = 78.0 ppm). The absolute values of the coupling constants are given in Hertz (Hz), regardless of their signs. Multiplicities are abbreviated as singlet (s), doublet (d), triplet (t), quartet (q), multiplet (m), and broad (br). Mass spectrometric measurements were carried out on a Bruker micrOTOF II mass spectrometer equipped with an atmospheric pressure chemical ionization (APCI) probe. Electronic absorption spectra were recorded using a quartz cell on a JASCO model V-670 UV/VIS spectrophotometer. Steady-state fluorescence spectra were measured using a quartz cell under air on a JASCO model FP-8500 spectrophotometer. Time-resolved fluorescence spectra were recorded on a Hamamatsu model Quantaurus-Tau C16361 fluorescence lifetime spectrometer. The absolute PLQY values were measured by a Hamamatsu Photonics model Quantaurus-QYC11347 absolute PLQY spectrometer. Cyclic voltammetry measurements were conducted using an ALS Instruments model 622C electrochemical analyzer (working electrode: glassy carbon, counter electrode: Pt wire; and pseudo-reference electrode: Ag wire)

2. Synthesis

Synthesis of B_2-ant (optimized conditions). Under argon at –78 °C, a THF solution (10 mL) of 1,2-benzenediboronic acid bis(pinacol) ester **1** (400 mg, 1.21 mmol) was added dropwise to a THF suspension (20 mL) of lithium aluminum hydride (190 mg, 4.98 mmol). The reaction mixture was allowed to warm to 25 °C and stirred at this temperature for 15 h. The resulting suspension was filtered through Celite®, and the filtrate was evaporated to dryness under reduced pressure. CH_2Cl_2 (20 mL) and chlorotrimethylsilane (1.6 mL, 12.7 mmol) were successively added to the residue, and the resulting suspension was stirred at 25 °C for 21 h. The reaction mixture was evaporated to dryness. CH_2Cl_2 (20 mL) and dry methanol (0.80 mL, 19.7 mmol) was added to the residue, and the mixture was stirred at 25 °C for 2 h and then evaporated to dryness under reduced pressure. THF (20 mL) was added to the residue, and to the resulting suspension was added at 0 °C a THF solution (40 mL) of 1,3,5-triisopropylphenyl magnesium bromide (TipMgBr), prepared from a mixture of 1-bromo-

2,4,6-triisopropylbenzene (3.1 mL, 12.3 mmol) and magnesium turnings (518 mg, 21.3 mmol). After stirring at 60 °C for 64 h, isopropyl alcohol (10 mL) was added to the reaction mixture, and the resulting mixture was passed through a plug of silica gel and then evaporated to dryness under reduced pressure. $CHCl_3$ (50 mL) was added to the residue, and the resulting suspension was filtered through a glass filter. The filtrate was evaporated to dryness under reduced pressure, and the residue was subjected to recycling preparative SEC (JAIGEL 1HH/2HH) using $CHCl_3$ as an eluent. Fractions containing **B_2-ant** were collected and evaporated to dryness under reduced pressure, affording **B_2-ant** (310 mg, 534 μmol) as a pale yellow powder in 88% yield. FT-IR (KBr): ν (cm^{-1}) 3058, 2966, 2927, 2866, 1604, 1576, 1554, 1459, 1416, 1383, 1362, 1343, 1278, 1239, 1184, 1168, 1153, 1099, 1078, 942, 903, 876, 777, 756, 693, 675, 667, 619, 591. ^{1}H NMR (400 MHz, $CDCl_3$, 25 °C): δ (ppm) 7.70 (dd, J = 5.3, 3.3 Hz, 4H), 7.46 (dd, J = 5.3, 3.3 Hz, 4H), 7.08 (s, 4H), 3.00 (sep, J = 6.9 Hz, 2H), 2.45 (sep, J = 6.7 Hz, 4H), 1.38 (d, J = 6.9 Hz, 12H), 1.09 (d, J = 6.7 Hz, 24H). ^{13}C NMR (100 MHz, $CDCl_3$, 25 °C): δ (ppm) 149.5, 148.4, 146.1, 139.0, 132.8, 120.1, 35.8, 34.4, 24.3 (two peaks). One peak due to the carbon atoms of the Tip groups, *ipso*-positions to the boron atoms, were not observed. A ^{11}B NMR signal of **B_2-ant** was not detected, presumably due to line broadening caused by rapid quadrupolar relaxation.[S3,S4] High-resolution APCI-TOF mass: calcd. for $C_{42}H_{54}B_2$ $[M]^+$: m/z = 580.4420; found: 580.4420. ^{1}H NMR (Figure S6), ^{13}C NMR (Figure S7), HMQC (Figure S15), HMBC (Figure S16), FT-IR (Figure S17), and high-resolution APCI-TOF mass spectra (Figure S18) of **B_2-ant** are shown in the respective Supporting Figures.

Synthesis of B_6-hept and B_4-pent. Under argon at –78 °C, a THF solution (40 mL) of a mixture of 1,2-benzenediboronic acid bis(pinacol) ester **1** (2.27 g, 6.87 mmol) and 1,2,4,5-benzenetetraboronic acid tetra(pinacol) ester **4** (499 mg, 857 μmol) was added dropwise to a THF suspension (40 mL) of lithium aluminum hydride (1.32 g, 34.7 mmol). The reaction mixture was allowed to warm to 25 °C and stirred at this temperature for 22 h. The resulting suspension was filtered through Celite®, and the filtrate was evaporated to dryness under reduced pressure. CH_2Cl_2 (90 mL) and chlorotrimethylsilane (10.0 mL, 87.1 mmol) were successively added to the residue, and the resulting mixture was stirred at 25 °C for 17 h. The reaction mixture was evaporated to dryness. CH_2Cl_2 (90 mL) and dry methanol (5.8 mL, 143 mmol) was added to the residue, and the mixture was stirred at 25 °C for 2 h and then evaporated to dryness under reduced pressure. THF (50 mL) was added to the residue, and to the resulting suspension was added at 0 °C a THF solution (60 mL) of 1,3,5-triisopropylphenyl magnesium bromide (TipMgBr), prepared from a mixture of 1-bromo-2,4,6-triisopropylbenzene (17.5 mL, 69.2 mmol) and magnesium turnings (2.03 g, 83.3 mmol). After stirring at 60 °C for 69 h, isopropyl alcohol (30 mL) was added to the reaction mixture, and the resulting mixture was passed through a plug of silica gel and then evaporated to dryness under reduced pressure. $CHCl_3$ (100 mL) was added to the residue, and the resulting suspension was filtered through a glass filter. The residue was subjected to silica gel column chromatography (hexane, then $CHCl_3$/hexane 1/5 v/v), where fractions containing a mixture

of **B_2-ant**, **B_4-pent**, and **B_6-hept** were collected and evaporated to dryness under reduced pressure. The residue was subjected to recycling preparative SEC (JAIGEL 1HH/2HH) using $CHCl_3$ as an eluent. Fractions containing **B_2-ant**, **B_4-pent**, and **B_6-hept** were separately collected (For details, see Figure S1) and evaporated to dryness under reduced pressure, affording **B_2-ant** (1.13 g, 1.95 mmol, 57% yield from **1**) as a pale yellow powder, **B_4-pent** (180 mg, 166 μmol, 19% yield from **4**) as a yellow powder, and **B_6-hept** (7.0 mg, 4.4 μmol, 1% yield from **4**) as a yellow powder, respectively.

B_4-pent: FT-IR (KBr): ν (cm^{-1}) 3047, 2956, 2926, 2866, 1604, 1578, 1557, 1457, 1421, 1382, 1362, 1278, 1262, 1232, 1219, 1168, 1152, 1092, 943, 917, 903, 874, 758, 668, 587. ^{1}H NMR (400 MHz, $CDCl_3$, 25 °C): δ (ppm) 8.07 (s, 2H), 7.67 (dd, J = 5.4, 3.3 Hz, 4H), 7.40 (dd, J = 5.4, 3.3 Hz, 4H), 6.89 (s, 8H), 2.92 (sep, J = 6.9 Hz, 4H), 2.32 (sep, J = 6.7 Hz, 8H), 1.33 (d, J = 6.9 Hz, 24H), 0.97 (d, J = 6.7 Hz, 24H), 0.70 (d, J = 6.7 Hz, 24H). ^{13}C NMR (126 MHz, $CDCl_3$, 25 °C): δ (ppm) 148.9, 148.7, 148.2, 146.9, 146.1, 138.7, 138.5, 132.6, 120.0, 35.9, 34.5, 24.7, 24.4, 24.1. A ^{11}B NMR signal of **B_4-pent** was not detected, presumably due to line broadening caused by rapid quadrupolar relaxation.[S3,S4] High-resolution APCI-TOF mass: calcd. for $C_{78}H_{104}B_4$ $[M]^+$: m/z = 1082.8388; found: 1082.8387. ^{1}H NMR (Figure S4), ^{13}C NMR (Figure S5), HMQC (Figure S19, HMBC (Figure S20), FT-IR (Figure S21), and high-resolution APCI-TOF mass spectra (Figure S22) of **B_4-pent** are shown in the respective Supporting Figures.

B_6-hept: FT-IR (KBr): ν (cm^{-1}) 3047, 2957, 2926, 2867, 1604, 1557, 1457, 1421, 1382, 1362, 1278, 1259, 1237, 1209, 1094, 1030, 956, 920, 875, 804, 759, 669. ^{1}H NMR (400 MHz, $CDCl_3$, 25 °C): δ (ppm) 8.01 (s, 4H), 7.65 (dd, J = 5.3, 3.3 Hz, 4H), 7.38 (dd, J = 5.3, 3.3 Hz, 4H), 6.83 (s, 8H), 6.71 (s, 4H), 2.91–2.78 (m, 6H), 2.28 (sep, J = 6.6 Hz, 8H), 2.15 (sep, J = 6.6 Hz, 4H), 1.30–1.28 (m, 36H), 0.93 (d, J = 6.6 Hz, 24H), 0.67 (d, J = 6.6 Hz, 24H), 0.60 (d, J = 6.6 Hz, 24H). ^{13}C NMR (126 MHz, $CDCl_3$, 25 °C): δ (ppm) 148.9, 148.8, 148.7, 148.3, 148.2, 148.1, 146.6, 146.2, 138.6, 138.5, 138.4, 132.6, 120.0, 119.9, 36.1, 35.8, 34.9, 34.4, 24.8, 24.6, 24.4 (two peaks), 24.1. ^{11}B NMR signals of **B_6-hept** were not detected, presumably due to line broadening caused by rapid quadrupolar relaxation.[S3,S4] High-resolution APCI-TOF mass: calcd. for $C_{114}H_{150}B_6$ $[M]^+$: m/z = 1585.2359; found: 1585.2362. ^{1}H NMR (Figure S2), ^{13}C NMR (Figure S3), HMQC (Figure S23), HMBC (Figure S24), FT-IR (Figure S25), and high-resolution APCI-TOF mass spectra (Figure S26) of **B_6-hept** are shown in the respective Supporting Figures.

3. Single-crystal X-ray Diffraction Analysis

Single crystals of **B_6-hept** and **B_4-pent** were prepared by slow evaporation of their toluene and $CHCl_3$ solutions, respectively. Single crystals of **B_2-ant** were obtained by recrystallization from a mixture of $CHCl_3$ and hexane. Each single crystal was coated with immersion oil (type B: code 1248, Cargille Laboratories, Inc.) and mounted on a MicroMount (MiTeGen, LLC). Diffraction data were collected at 90 K under a cold nitrogen gas stream on

a RIGAKU model XtaLAB Synergy-DW diffractometer system equipped with a HyPix-6000 detector, using Cu-$K\alpha$ radiation (λ = 1.54184 Å).

Crystal data for B_6-hept: yellow prism, 0.30 x 0.11 x 0.05 mm^3, monoclinic, $P2_1/n$, a = 18.1432(4) Å, b = 62.6609(11) Å, c = 22.1687(5) Å, β = 110.694(3)°, V = 23576.8(10) Å^3, Z = 8, density = 1.010 g cm^{-3}, T = 90K, $2\theta_{max}$ = 153.796 °, Cu-$K\alpha$ radiation, λ = 1.54184 Å, μ = 0.408 mm^{-1}, 47769 reflections measured, 19270 unique reflections, 2755 parameters, R_{int} = 0.0761, GOF = 1.037, R_1 = 0.1028 ($I > 2\sigma(I)$), wR_2 = 0.3658 (all data), $\Delta\rho_{min, max}$ = –0.372, 0.051 e Å^{-3}, CCDC-2517732.

Crystal data for B_4-pent: pale-yellow prism, 0.08 x 0.07 x 0.04 mm^3, monoclinic, $P2_1/n$, a = 15.1699(2) Å, b = 17.0240(2) Å, c = 15.8619(2) Å, β = 112.5010(10)°, V = 3784.53(9) Å^3, Z = 4, density = 1.160 g cm^{-3}, T = 293 K, $2\theta_{max}$ = 149.844 °, Cu-$K\alpha$ radiation, λ = 1.54184 Å, μ = 2.371 mm^{-1}, 7507 reflections measured, 6713 unique reflections, 418 parameters, R_{int} = 0.0296, GOF = 1.046, R_1 = 0.0435 ($I > 2\sigma(I)$), wR_2 = 0.1078 (all data), $\Delta\rho_{min, max}$ = –0.440, 0.454 e Å^{-3}, CCDC-2517731.

Crystal data for B_2-ant: colourless prism, 0.15 x 0.11 x 0.09 mm^3, orthorhombic, $Pccn$, a = 26.0421(9) Å, b = 14.0622(5) Å, c = 9.6541(3) Å, V = 3535.4(2) Å^3, Z = 4, density = 1.091 g cm^{-3}, T = 293 K, $2\theta_{max}$ = 152.986 °, Cu-$K\alpha$ radiation, λ = 1.54184 Å, μ = 0.441 mm^{-1}, 3519 reflections measured, 3101 unique reflections, 205 parameters, R_{int} = 0.0347, GOF = 1.137, $R1$ = 0.0646 ($I > 2\sigma(I)$), w$R2$ = 0.1971 (all data), $\Delta\rho_{min, max}$ = –0.259, 0.596 e Å^{-3}, CCDC-2517730.

4. Computational Details

All calculations, except for the nudged elastic band (NEB) calculations, were carried out using the Gaussian 16 software package.[S5]

Ground-state geometry optimizations of **B_6-hept**, **B_4-pent**, and **B_2-ant** were performed at the B3LYP-D3(BJ)[S6]/6-31G(d) level of theory. Vibrational frequency analyses were conducted to confirm stationary points and to obtain thermochemical corrections at the same level. The optimized structures were then subjected to single-point energy calculations at the ωB97X-D[S7]/6-311G(d,p) level. Conformational analyses were partly performed with the GFN-FF[S8] method implemented in the xTB program (version 6.5.1),[S9] and initial coordinates were generated using the CREST program (version 2.12).[S10] To account for solvent effects, geometry optimizations of the bent-zigzag(S_0) and twist1(S_0) forms of **B_6-hept** were also carried out at the CPCM(toluene)[S11]-B3LYP-D3(BJ)/6-31G(d) level of theory. Vibrational frequency analyses were performed to confirm stationary points and to obtain thermochemical corrections at the same level. Single-point energies were computed at the CPCM(toluene)-ωB97X-D/6-311G(d,p) level of theory. The resulting data were visualized using the GaussView program package.[S12] The nudged elastic band (NEB)[S13] calculations were performed using the ORCA 5.0.4 program package[S14] at the CPCM(toluene)[S15]-B3LYP-D3(BJ)/6-31G(d) level of theory (Gaussian-style B3LYP). The

Cartesian coordinates of the NEB-optimized minimum-energy paths (MEPs) are provided in the Supporting Information in a separate XYZ file (NEB_trj_B6hept_MEP.xyz). The Gaussian optimized structures were used for the start and end point structure.

Geometry optimizations of **B_6-hept** in the S_1 state were performed using time-dependent (TD)-DFT calculations at the CPCM(toluene)-TD-CAM-B3LYP-D3(BJ)[16]/6-31G(d) level of theory, starting from the bent-zigzag(S_0) and twist1(S_0) geometries. Vibrational frequency analyses were carried out at the same level to confirm the stationary points and to obtain thermochemical corrections. The optimized structures were then subjected to single-point energy calculations at the CPCM(toluene)-TD-ωB97X-D/6-311G(d,p) level. The Tamm-Dancoff approximation (TDA)[17] was applied in all TD-DFT calculations. The optimized Cartesian coordinates and energies are listed in Tables S2–S11 (S_0 geometries) and Tables S12 and 13 (S_1 geometries).

5. Supporting References

S1. L. Biesen, J. Krenzer, N. Nirmalananthan-Budau, U. Resch-Genger, T. J. J. Müller, *Chem. Sci.* **2022**, *13*, 5374–5381.

S2. J. Kim, H. T. Teo, Y. Hong, J. Oh, H. Kim, C. Chi, D. Kim, *Angew. Chem. Int. Ed.* **2020**, *59*, 20956–20964.

S3. B. Wrackmeyer, in Annual Reports on NMR Spectroscopy, Vol. 20, G. A. Webb (Ed.), Academic Press, London, **1988**, pp. 1–124.

S4. H. Nöth, B. Wrackmeyer, Nuclear Magnetic Resonance Spectroscopy of Boron Compounds, Springer-Verlag, Berlin, **1978**.

S5. Gaussian 16, Revision C.02, M. J. Frisch, G. W. Trucks, H. B. Schlegel, G. E. Scuseria, M. A. Robb, J. R. Cheeseman, G. Scalmani, V. Barone, G. A. Petersson, H. Nakatsuji, X. Li, M. Caricato, A. V. Marenich, J. Bloino, B. G. Janesko, R. Gomperts, B. Mennucci, H. P. Hratchian, J. V. Ortiz, A. F. Izmaylov, J. L. Sonnenberg, D. Williams-Young, F. Ding, F. Lipparini, F. Egidi, J. Goings, B. Peng, A. Petrone, T. Henderson, D. Ranasinghe, V. G. Zakrzewski, J. Gao, N. Rega, G. Zheng, W. Liang, M. Hada, M. Ehara, K. Toyota, R. Fukuda, J. Hasegawa, M. Ishida, T. Nakajima, Y. Honda, O. Kitao, H. Nakai, T. Vreven, K. Throssell, J. A. Montgomery, Jr., J. E. Peralta, F. Ogliaro, M. J. Bearpark, J. J. Heyd, E. N. Brothers, K. N. Kudin, V. N. Staroverov, T. A. Keith, R. Kobayashi, J. Normand, K. Raghavachari, A. P. Rendell, J. C. Burant, S. S. Iyengar, J. Tomasi, M. Cossi, J. M. Millam, M. Klene, C. Adamo, R. Cammi, J. W. Ochterski, R. L. Martin, K. Morokuma, O. Farkas, J. B. Foresman, and D. J. Fox, Gaussian, Inc., Wallingford CT, 2016.

S6. S. Grimme, S. Ehrlich and L. Goerigk, *J. Comp. Chem.* **2011**, *32*, 1456–1465.

S7. J.-D. Chai, M. Head-Gordon, *Phys. Chem. Chem. Phys.* **2008**, *10*, 6615–6620.

S8. S. Spicher, S. Grimme, *Angew. Chem. Int. Ed.* **2020**, *59*, 15665–15673.

S9. C. Bannwarth, E. Caldeweyher, S. Ehlert, A. Hansen, P. Pracht, J. Seibert, S. Spicher, *WIREs Comput. Mol. Sci.* **2020**, *11*, e1493.

S10. P. Pracht, F. Bohle, S. Grimme, *Phys. Chem. Chem. Phys.* **2020,** *22*, 7169–7192.

S11. (a) V. Barone, M. Cossi, *J. Phys. Chem. A*, **1998**, *102*, 1995–2001; (b) M. Cossi, N. Rega, G. Scalmani, V. Barone, *J. Comp. Chem.* **2003**, *24*, 669-681.
S12. GaussView, Version 6.1, R. Dennington, T. A. Keith, J. M. Millam, Semichem Inc., Shawnee Mission, KS, **2016**.
S13. G. Henkelman, H. Jónsson. *J. Chem. Phys.* **2000**, *113*, 9978–9985.
S14. Neese, F. *Wiley Interdiscip. Rev.: Comput. Mol. Sci.*, **2012**, *2*, 73–78.
S15. M. Garcia-Rates, F. Neese, *J. Comput. Chem.* **2020**, *41*, 922–939.
S16. T. Yanai, D. P. Tew, N. C. Handy, *Chem. Phys. Lett.* **2004**, *393*, 51–57.
S17. S. Hirata, M. Head-Gordon, *Chem. Phys. Lett.* **1999**, *314*, 291–299.

6. Supporting Figures and Table

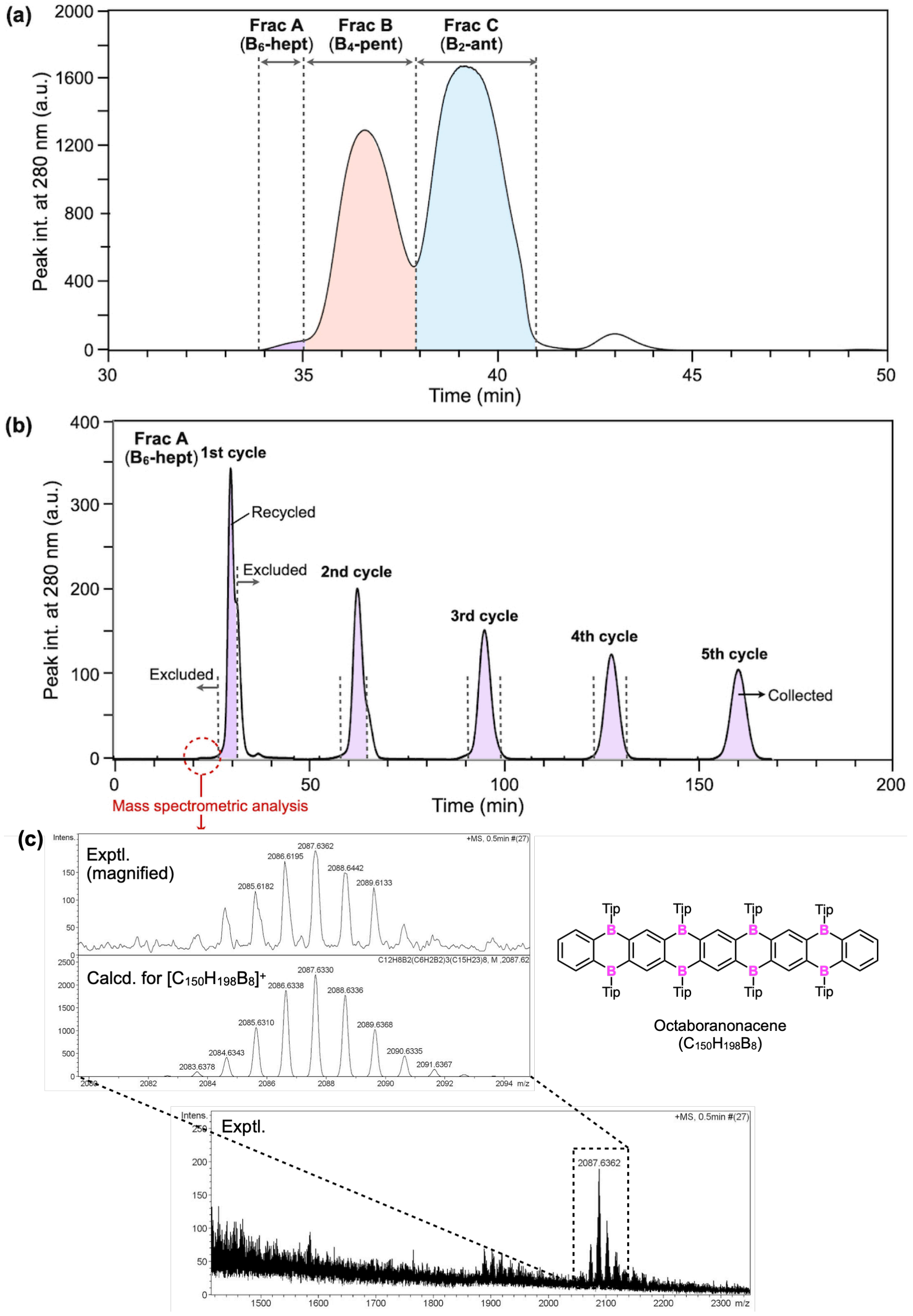

Figure S1. (a) Preparative SEC profile of a mixture of boron-doped heteroacenes. Fractions mainly containing **B_6-hept** (**Frac A**), **B_4-pent** (**Frac B**), and **B_2-ant** (**Frac C**) were collected separately. (b) Recycling preparative SEC profile of **Frac A**. The central fraction was recycled at each cycle, while the peak-edge fractions were excluded. The elution peak after the 5th cycle was collected to allow isolation of **B_6-hept**. **B_4-pent** and **B_2-ant** were isolated in an analogous manner from **Frac B** and **Frac C**, respectively. (c) APCI-TOF mass spectrum of a fraction with a shorter retention time than that containing **B_6-hept**, showing ion peaks consistent with those calculated for an octaboranonacene derivative.

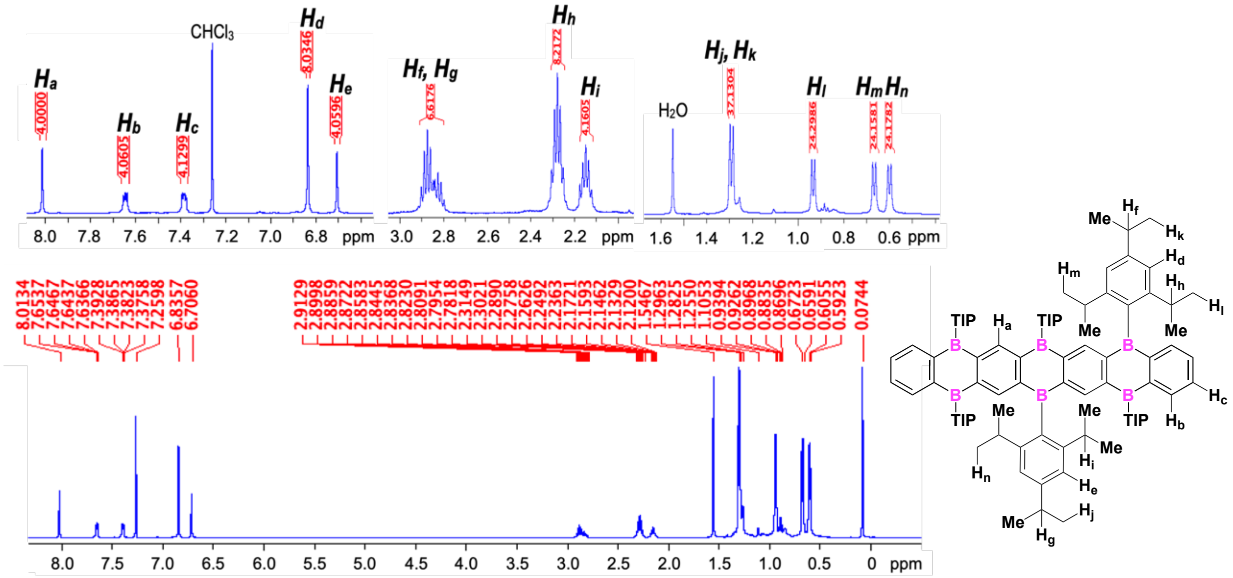

Figure S2. ^{1}H NMR spectrum (400 MHz) of **B$_6$-hept** in $CDCl_3$ at 25 °C.

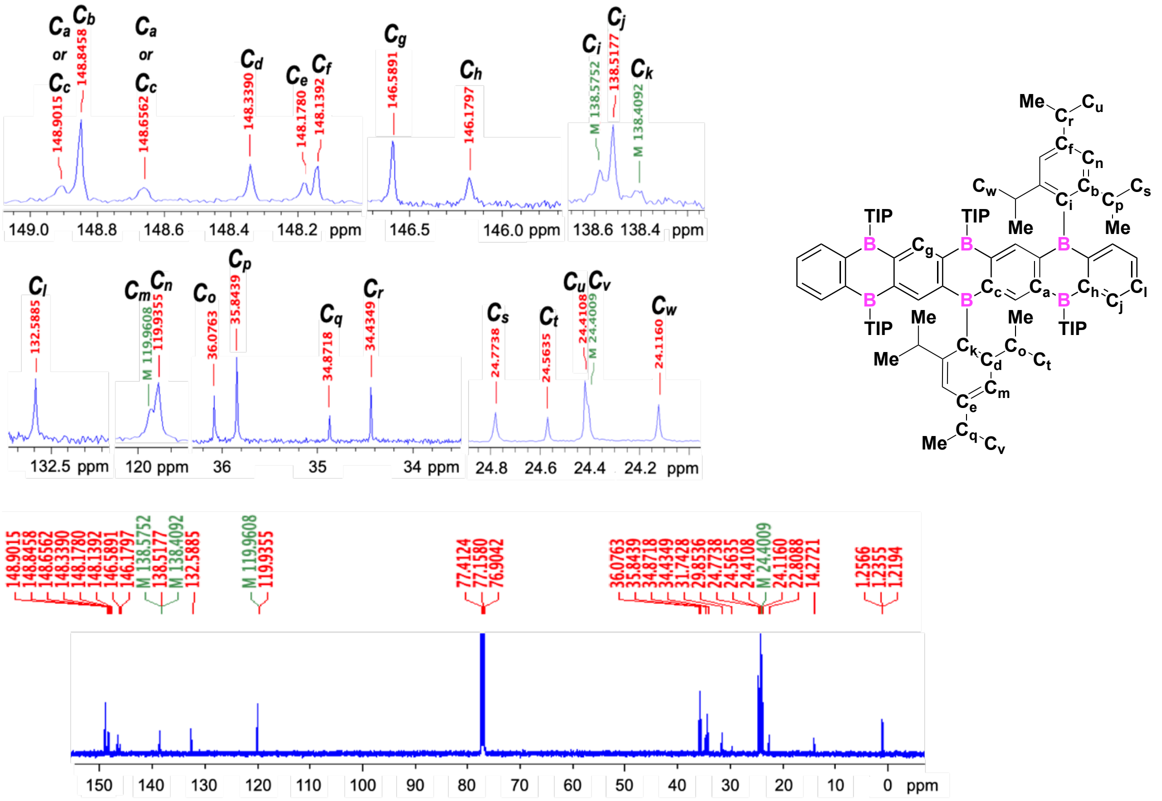

Figure S3. ^{13}C NMR spectrum (126 MHz) of **B$_6$-hept** in $CDCl_3$ at 25 °C.

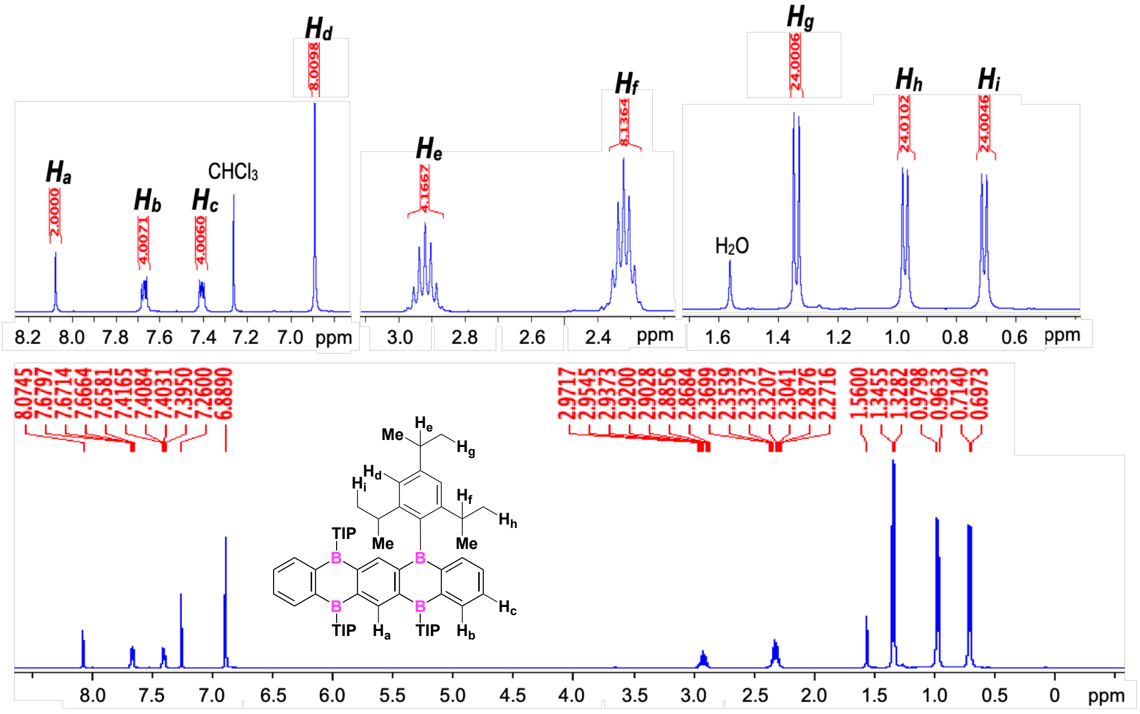

Figure S4. ^{1}H NMR spectrum (400 MHz) of **B₄-pent** in $CDCl_3$ at 25 °C.

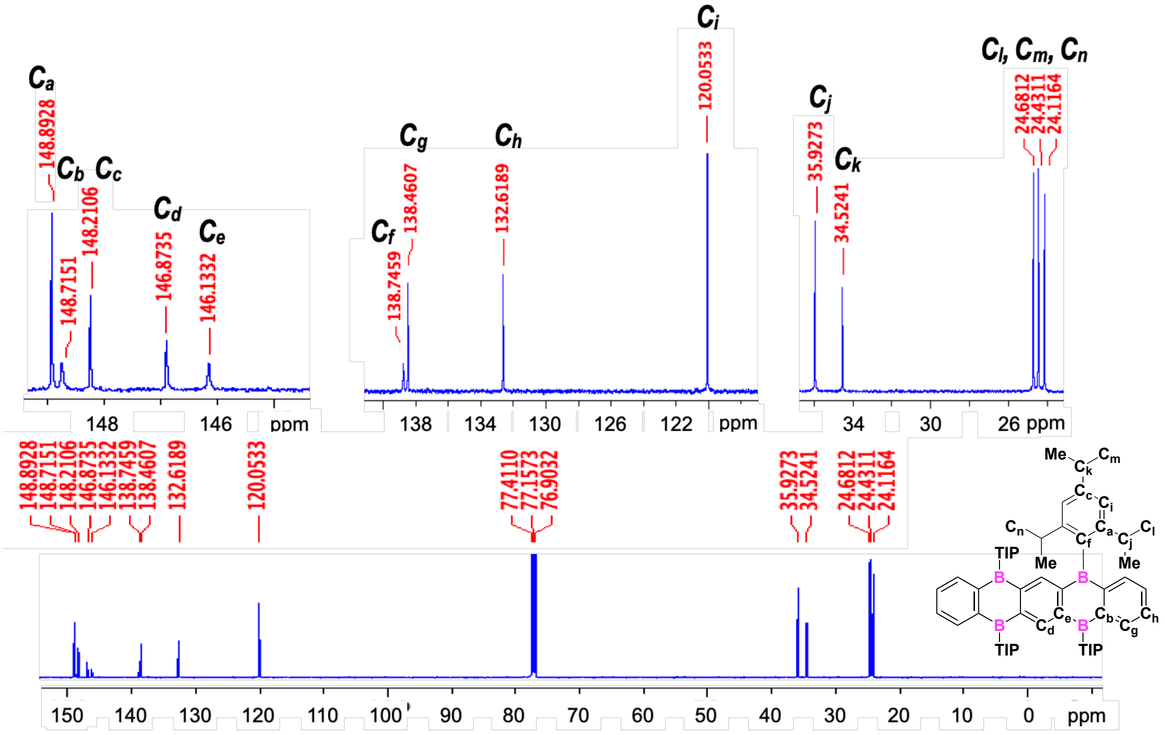

Figure S5. ^{13}C NMR spectrum (100 MHz) of **B₄-pent** in $CDCl_3$ at 25 °C.

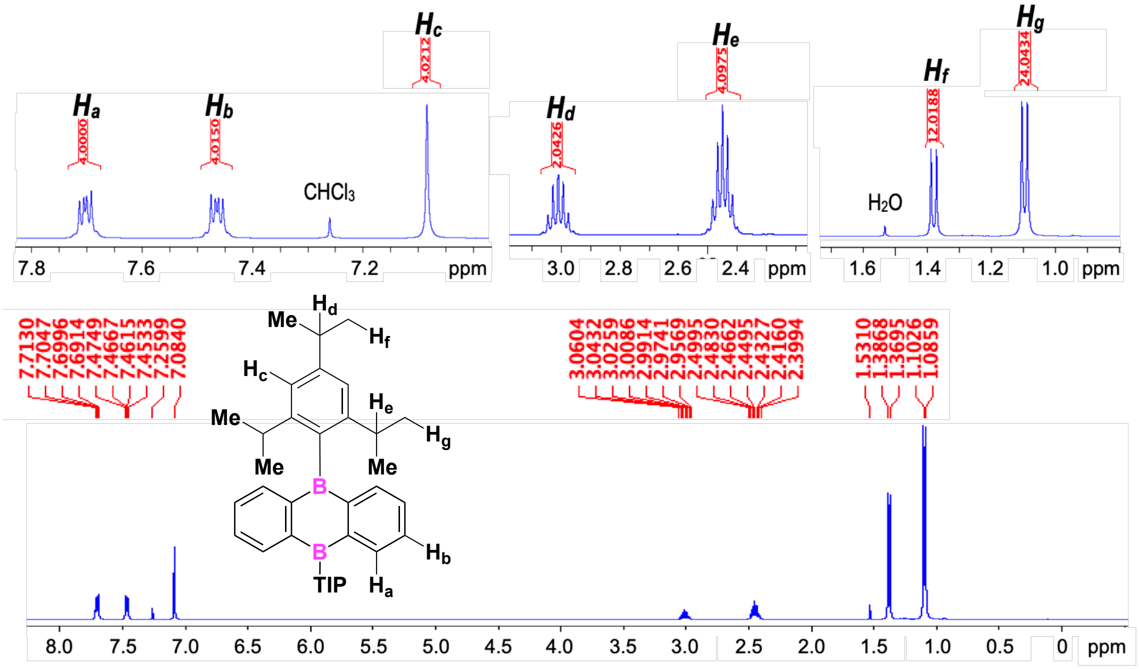

Figure S6. ^{1}H NMR spectrum (400 MHz) of **B$_2$-ant** in $CDCl_3$ at 25 °C.

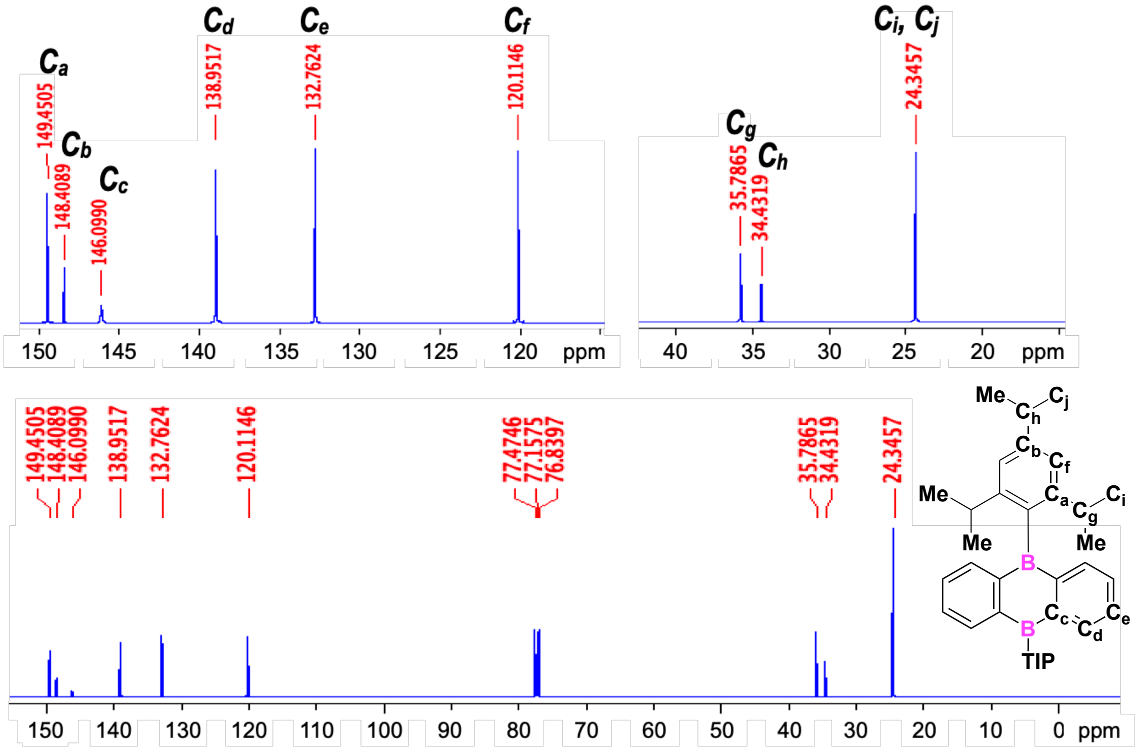

Figure S7. ^{13}C NMR spectrum (100 MHz) of **B$_2$-ant** in $CDCl_3$ at 25 °C.

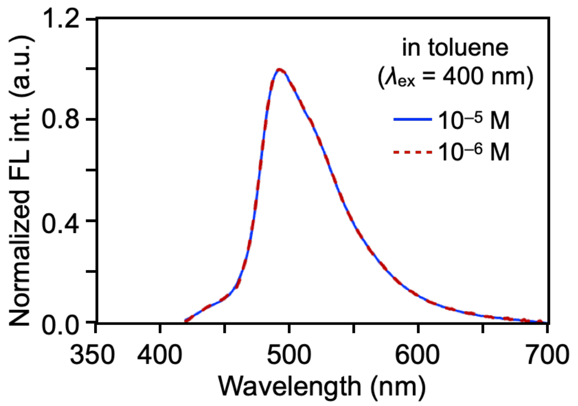

Figure S8. Normalized fluorescence spectra of **B$_6$-hept** (λ_{ex} = 400 nm) in toluene at 298 K. Blue solid curve: 10^{-5} M, red dotted curve: 10^{-6} M.

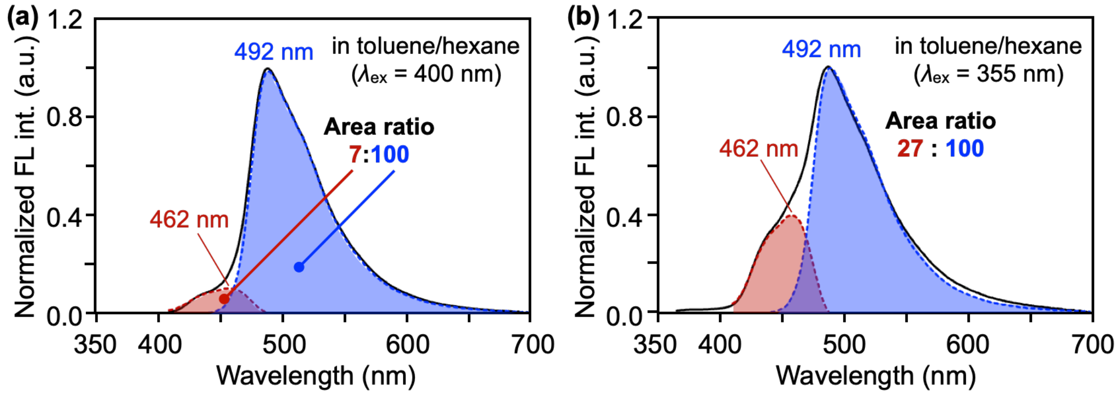

Figure S9. Fluorescence spectra of **B$_6$-hept** (black solid curves) in toluene/hexane (1/1 v/v) upon excitation at (a) 400 nm and (b) 355 nm, with deconvoluted longer-wavelength (blue) and shorter-wavelength (red) components.

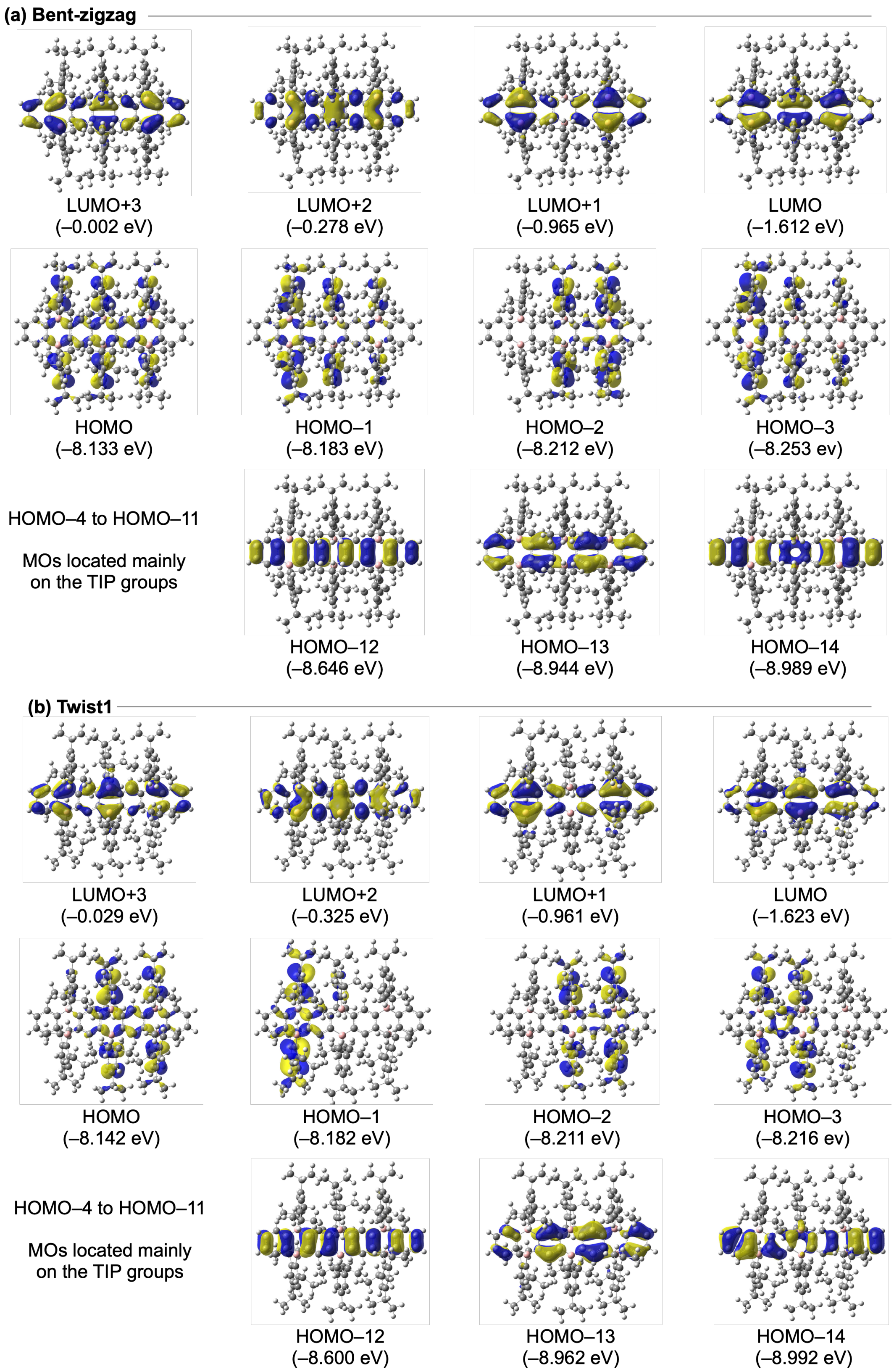

Figure S10. Selected MOs and energies of (a) bent-zigzag and (b) twist1 conformers of **B_6-hept** calculated at the CPCM(toluene)-B3LYP-D3(BJ)/6-31G(d) level of theory.

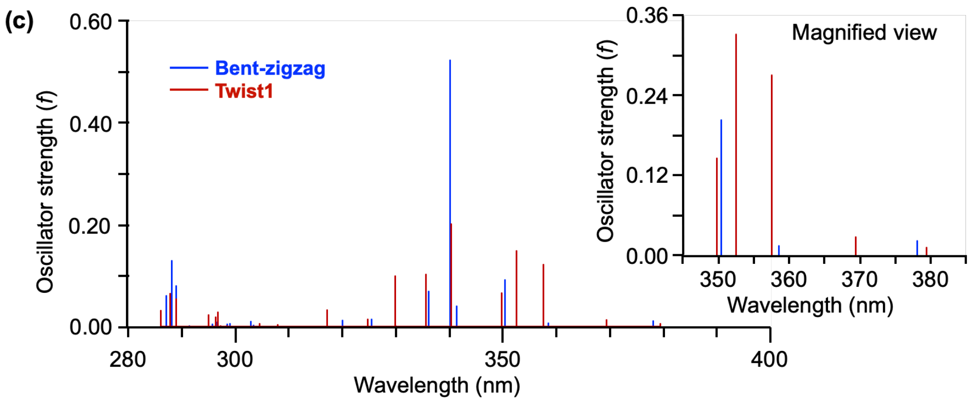

Figure S10(continued). (c) Plots of calculated electronic transition energies and oscillator strengths for the bent-zigzag and twist1 conformers of **B_6-hept** obtained by TD-DFT calculations at the CPCM(toluene)-ωB97X-D/6-31+G(d,p)//B3LYP-D3(BJ)/6-31G(d) level. While the calculated HOMO and LUMO energies of the bent-zigzag and twist1 conformers are similar, clear differences are found in the spatial distributions of the frontier molecular orbitals [panels (a) and (b)]. These differences are reflected more prominently in the electronic transition energies and oscillator strengths (Table S1), resulting in distinct absorption spectral patterns for the two conformers. The TD-DFT calculations suggest that the twist1 conformer exhibits absorption bands shifted to longer wavelengths compared to the bent-zigzag conformer [panel (c)]. This is consistent with the experimentally obtained excitation spectra, showing that the absorption bands assigned to the twist1 conformer extend toward longer wavelengths relative to those of the bent-zigzag conformer (Figure 5c,d), although the excitation spectrum of the twist1 conformer is partially truncated due to its proximity to the emission wavelength. Therefore, the observed spectral behavior is in qualitative agreement with the TD-DFT calculation results.

Table S1. Excitation energies, configurations, and oscillator strengths calculated for the bent-zigzag and twist1 conformers of **B_6-hept** (States 1–5) at the CPCM(toluene)-B3LYP-D3(BJ)/6-31G(d) level of theory.

	**Bent-zigzag**			**Twist1**		
State	Configuration	Coefficient	Energy<br>λ<br>(f)	Configuration	Coefficient	Energy<br>λ<br>(f)
1	HOMO → LUMO<br>HOMO–2 → LUMO<br>HOMO–2 → LUMO+3<br>HOMO–1 → LUMO<br>HOMO–1 → LUMO+1<br>HOMO–20 → LUMO	0.54386<br>-0.13225<br>-0.11447<br>0.11191<br>-0.17452<br>0.23081	3.28 eV<br>378 nm<br>(0.0104)	HOMO → LUMO<br>HOMO–12 → LUMO<br>HOMO–2 → LUMO<br>HOMO–2 → LUMO+3<br>HOMO–1 → LUMO<br>HOMO–20 → LUMO	0.50454<br>0.11043<br>-0.18403<br>-0.11321<br>-0.16779<br>0.20021	3.27 eV<br>379 nm<br>(0.0058)
2	HOMO–12 → LUMO<br>HOMO–18 → LUMO<br>HOMO–14 → LUMO+1<br>HOMO–7 → LUMO<br>HOMO–4 → LUMO<br>HOMO–3 → LUMO	0.48371<br>0.11846<br>-0.22833<br>0.14112<br>-0.30701<br>0.16939	3.38 eV<br>366 nm<br>(0.0001)	HOMO–12 → LUMO<br>HOMO–18 → LUMO<br>HOMO–14 → LUMO+1<br>HOMO–4 → LUMO<br>HOMO–3 → LUMO<br>HOMO–1 → LUMO<br>HOMO → LUMO	0.49587<br>0.10427<br>0.20586<br>-0.17911<br>-0.14208<br>-0.18953<br>-0.18912	3.36 eV<br>369 nm<br>(0.0128)
3	HOMO–1 → LUMO<br>HOMO–1 → LUMO+1<br>HOMO → LUMO+1<br>HOMO–24 → LUMO<br>HOMO–20 → LUMO+1<br>HOMO–2 → LUMO<br>HOMO–2 → LUMO+1	0.48189<br>0.15206<br>-0.32826<br>0.10030<br>-0.11314<br>0.10733<br>-0.13402	3.46 eV<br>358 nm<br>(0.0068)	HOMO–20 → LUMO+1<br>HOMO–13 → LUMO<br>HOMO–12 → LUMO<br>HOMO–3 → LUMO<br>HOMO–2 → LUMO<br>HOMO–1 → LUMO<br>HOMO–1 → LUMO+1<br>HOMO → LUMO<br>HOMO → LUMO+1	0.10932<br>0.14200<br>0.16566<br>-0.14022<br>0.24833<br>0.30429<br>-0.26483<br>0.18379<br>0.22046	3.47 eV<br>358 nm<br>(0.1206)
4	HOMO–2 → LUMO<br>HOMO–2 → LUMO+1<br>HOMO–1 → LUMO<br>HOMO–1 → LUMO+1<br>HOMO → LUMO+3<br>HOMO–13 → LUMO	0.48112<br>-0.22350<br>-0.13278<br>-0.23233<br>-0.15088<br>0.17487	3.54 eV<br>350 nm<br>(0.0907)	HOMO–13 → LUMO<br>HOMO–12 → LUMO<br>HOMO–3 → LUMO<br>HOMO–3 → LUMO+1<br>HOMO–2 → LUMO<br>HOMO–1 → LUMO<br>HOMO–1 → LUMO+1	0.16544<br>0.12343<br>0.39497<br>-0.16154<br>0.32805<br>-0.11502<br>0.13682	3.52 eV<br>352 nm<br>(0.1481)
5	HOMO–5 → LUMO+1<br>HOMO–4 → LUMO<br>HOMO–4 → LUMO+3<br>HOMO–3 → LUMO<br>HOMO–3 → LUMO+1	-0.12015<br>0.37703<br>0.15303<br>0.42991<br>0.17320	3.63 eV<br>341 nm<br>(0.0397)	HOMO–12 → UMO<br>HOMO–3 → LUMO<br>HOMO–3 → LUMO+1<br>HOMO–2 → LUMO<br>HOMO–2 → LUMO+1<br>HOMO–1 → LUMO<br>HOMO–1 → LUMO+1<br>HOMO → LUMO+1<br>HOMO → LUMO+3	-0.14165<br>-0.29440<br>-0.11578<br>0.29759<br>0.17848<br>-0.27663<br>0.17285<br>0.16790<br>-0.15892	3.55 eV<br>350 nm<br>(0.0653)

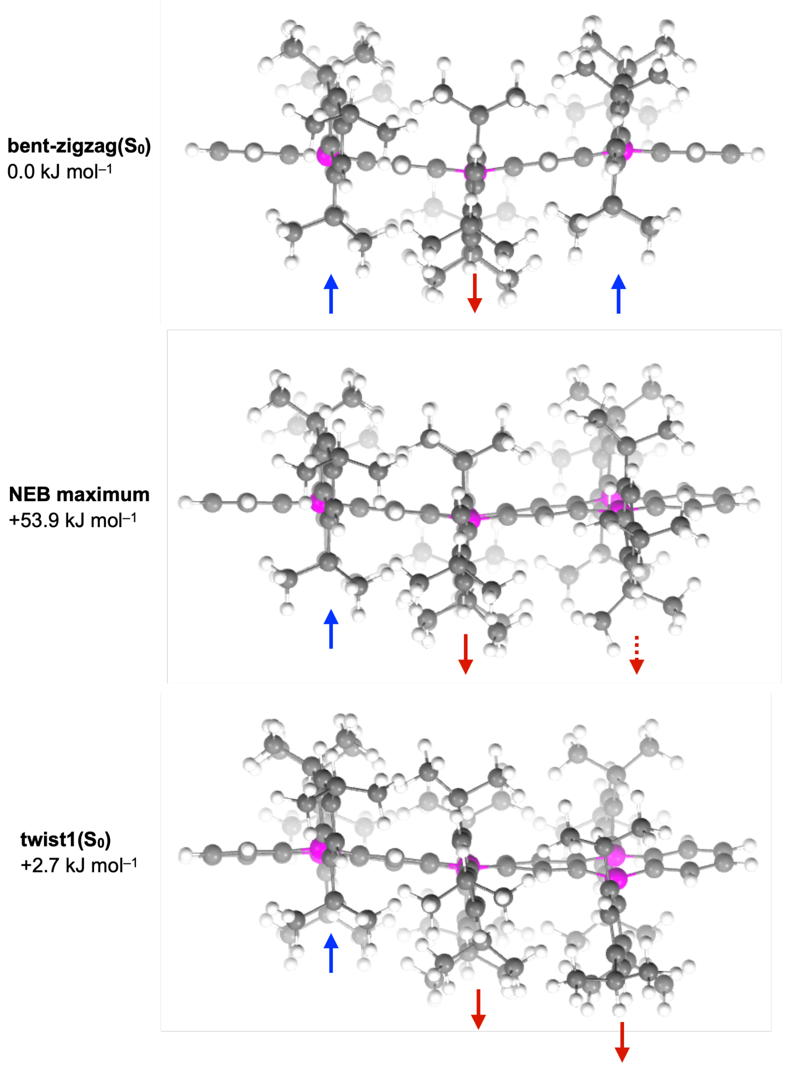

Figure S11. Calculated highest-energy structure along the NEB pathway (NEB maximum), shown as an approximate transition-state-like structure during the interconversion between the bent-zigzag(S$_0$) and twist1(S$_0$) conformers of **B$_6$-hept**. Blue and red arrows indicate the orientations of the three Tip groups on the viewer-facing side with respect to the plane of the B$_6$-heptacene core.

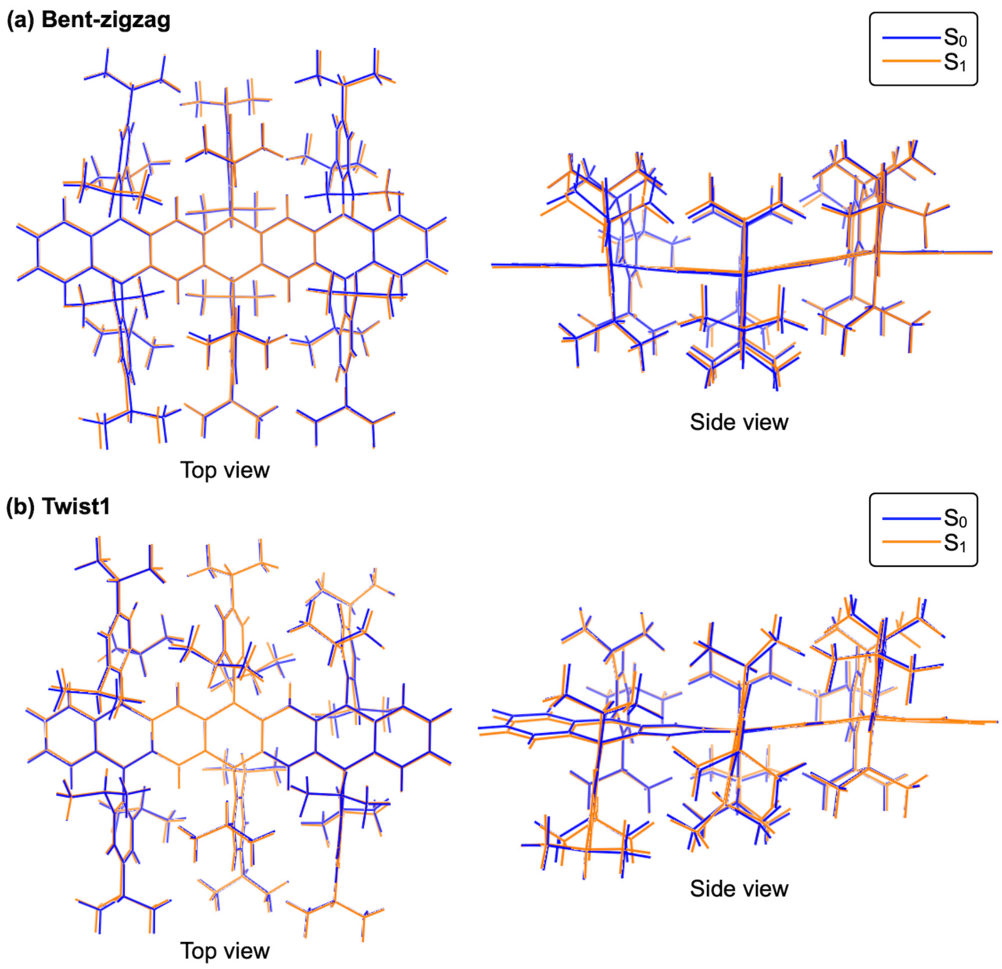

Figure S12. Optimized S_1-state geometries of (a) bent-zigzag and (b) twist1 conformers of **B_6-hept** (orange) overlaid with the corresponding S_0 geometries (blue).

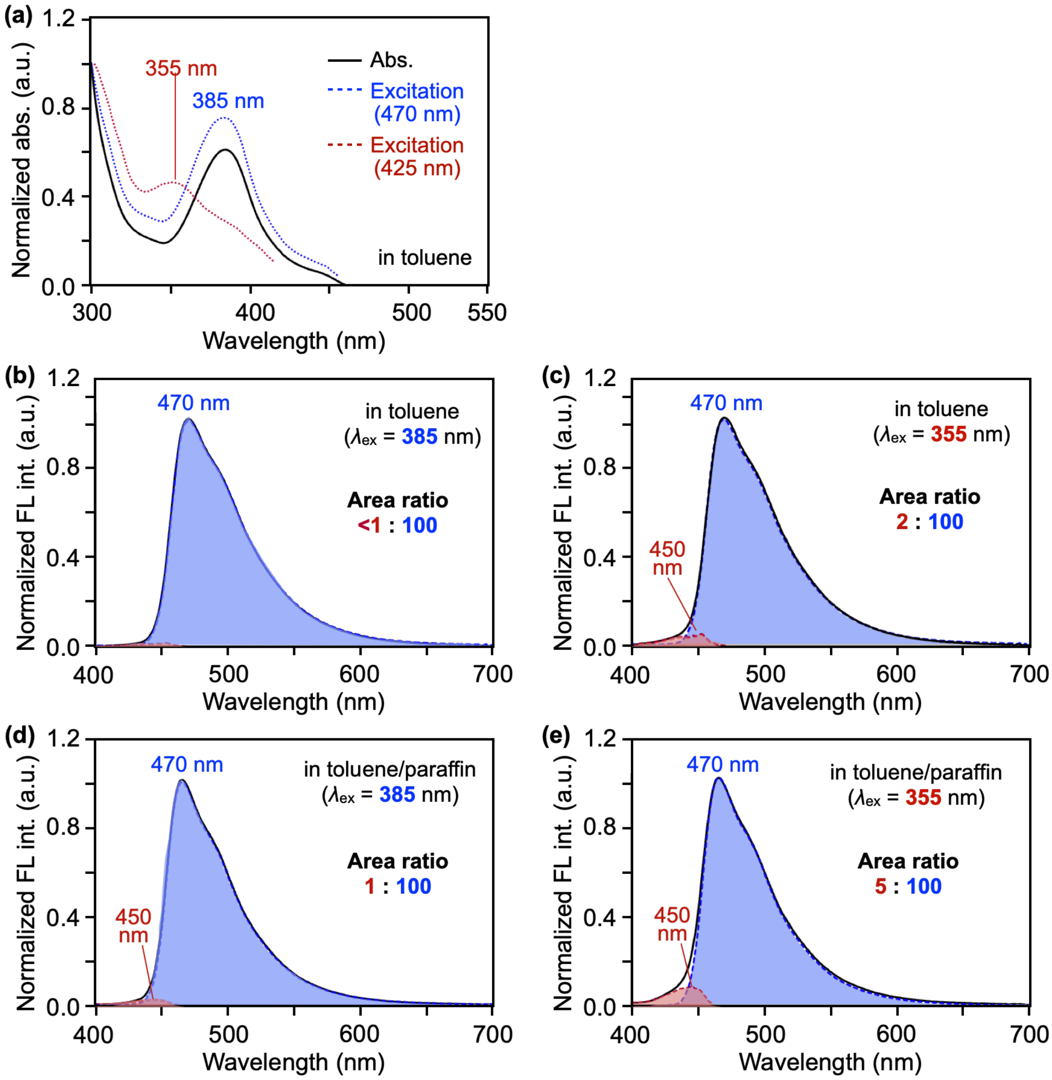

Figure S13. (a) Absorption (black solid curve) and excitation spectra (blue and red dotted curves: monitored at 470 and 425 nm, respectively) of $\mathbf{B_4}$**-pent** in toluene. (b,c) Fluorescence spectra of $\mathbf{B_4}$**-pent** (black solid curve) in toluene upon excitation at (b) 385 nm and (c) 355 nm, with deconvoluted longer-wavelength (blue) and shorter-wavelength (red) components. (d,e) Fluorescence spectra of $\mathbf{B_4}$**-pent** (black solid curve) in toluene/paraffin (1/1 v/v) upon excitation at (d) 385 nm and (e) 355 nm, with deconvoluted longer-wavelength (blue) and shorter-wavelength (red) components. All spectra were recorded at 298 K in 1.0×10^{-5} M solutions. As shown in panel (a), $\mathbf{B_4}$**-pent** displays clearly different excitation profiles, with maxima at 385 nm (blue dotted curve) and 355 nm (red dotted curve) when monitored at the fluorescence maximum (470 nm) and near the onset (425 nm), respectively. When excited at 355 nm, $\mathbf{B_4}$**-pent** exhibits a modest enhancement in the short-wavelength region of the fluorescence spectrum [panel (c)]. Such an enhancement is not observed upon excitation at 385 nm [panel (b)]. Deconvolution of the fluorescence spectra yields two emission components, a major one (blue) at 470 nm and a minor one (red) at 450 nm. The red-to-blue area ratio does not exceed 2:100 [panel (c)]. However, this ratio increases to about 5:100 when the solvent is changed from toluene to a toluene/paraffin mixture [panel (e)].

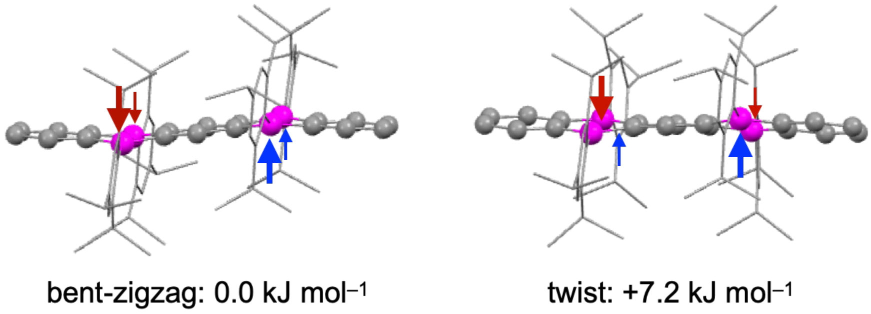

Figure S14. Two conformational isomers of **B_4-pent** obtained by conformational analysis at the *ω*B97XD/6-311G(d,p)//B3LYP-GD3BJ/6-31G(d) level. Blue and red arrows indicate the orientation of the Tip groups with respect to the plane of the B_4-pentacene core.

7. Analytical Data

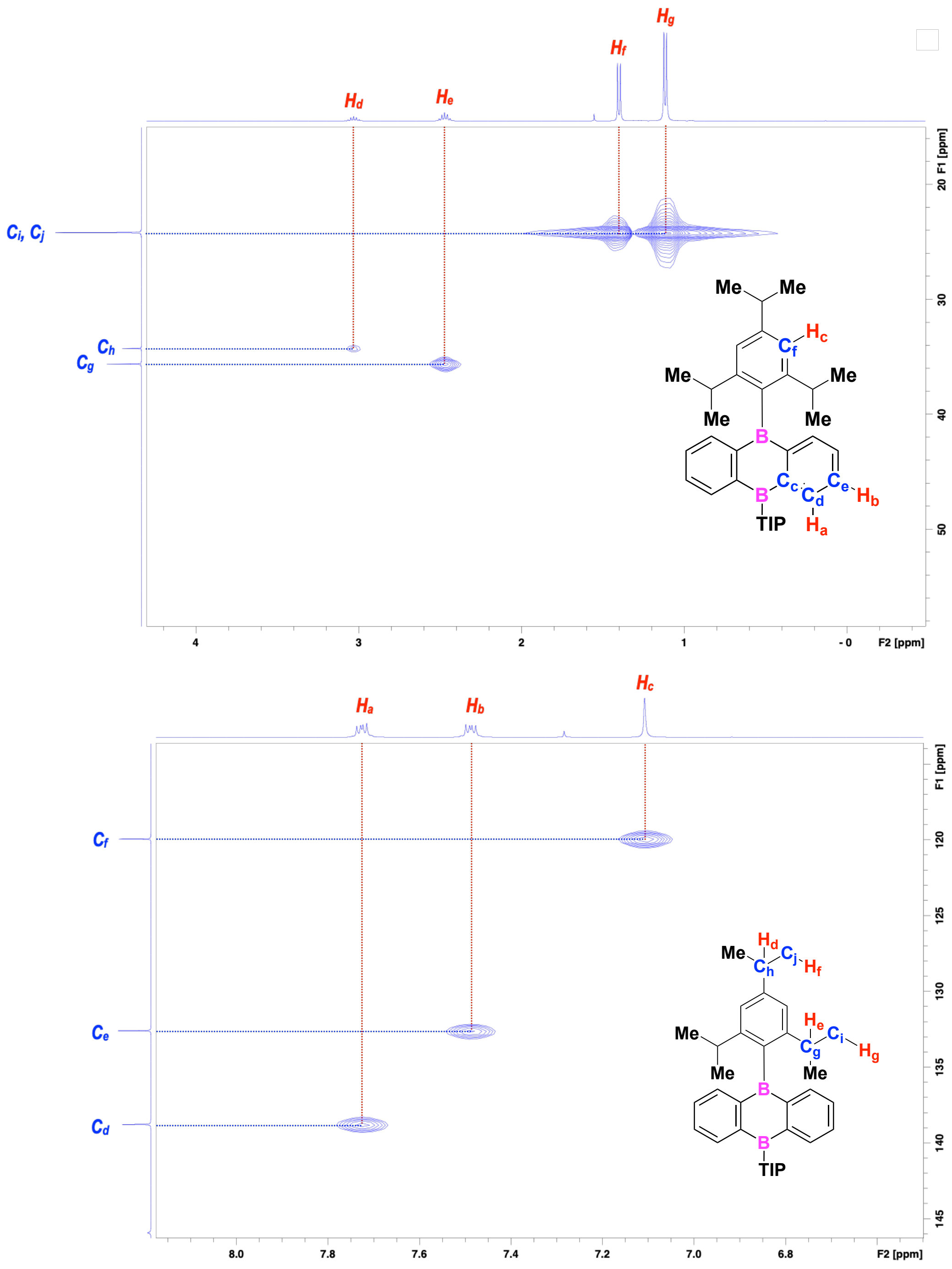

Figure S15. Partial HMQC correlations of **B_2-Ant** (^{1}H: 400 MHz, ^{13}C: 100 MHz) in $CDCl_3$ at 25 °C.

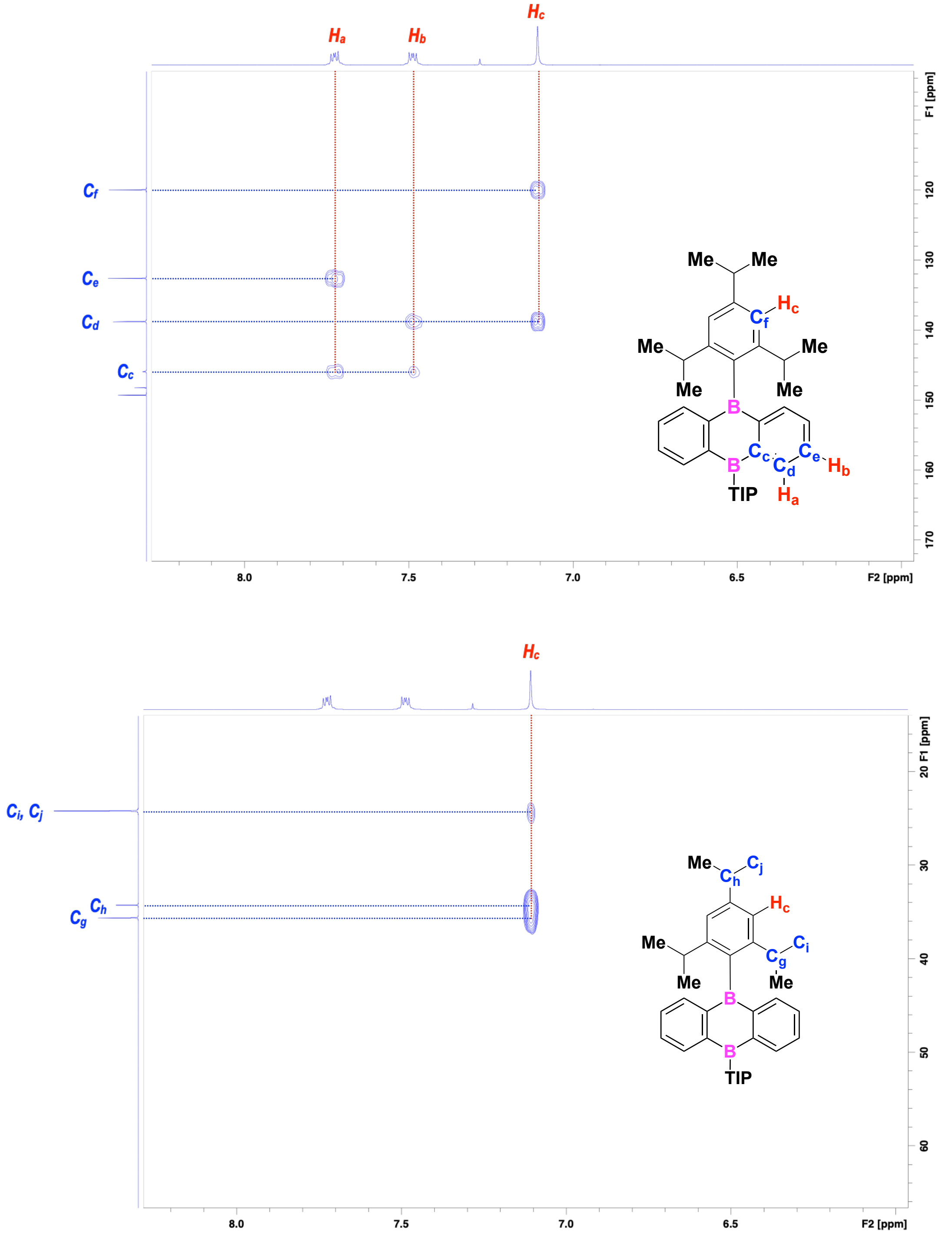

Figure S16(1). Partial HMBC correlations of **B_2-Ant** (^{1}H: 400 MHz, ^{13}C: 100 MHz) in $CDCl_3$ at 25 °C.

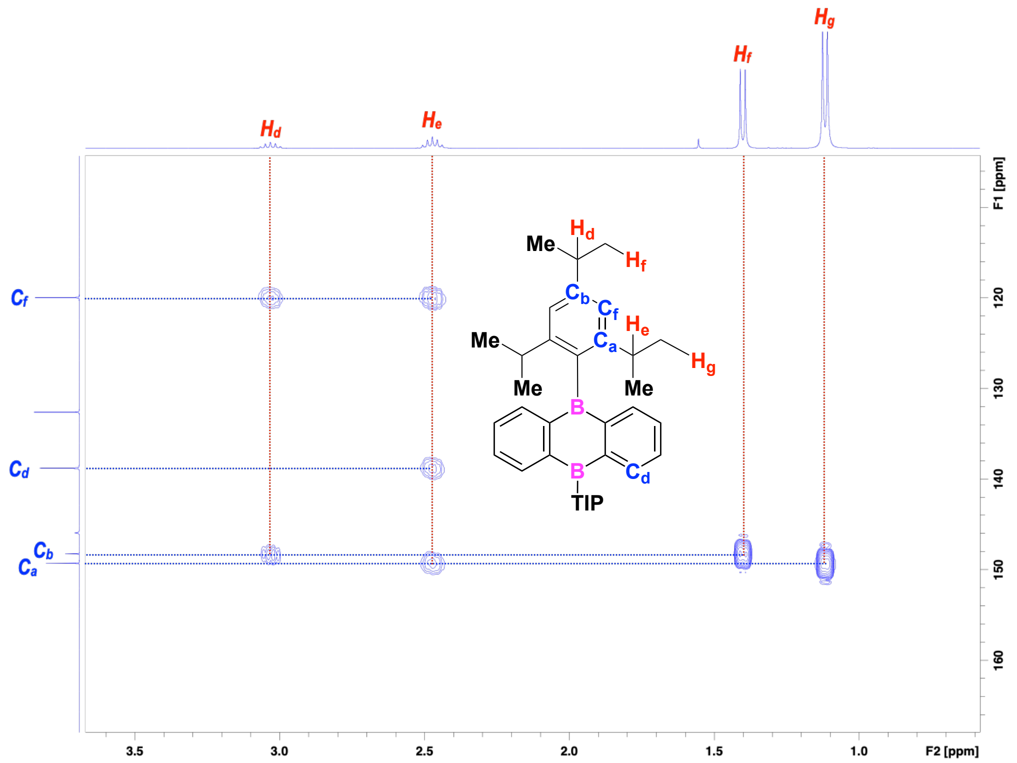

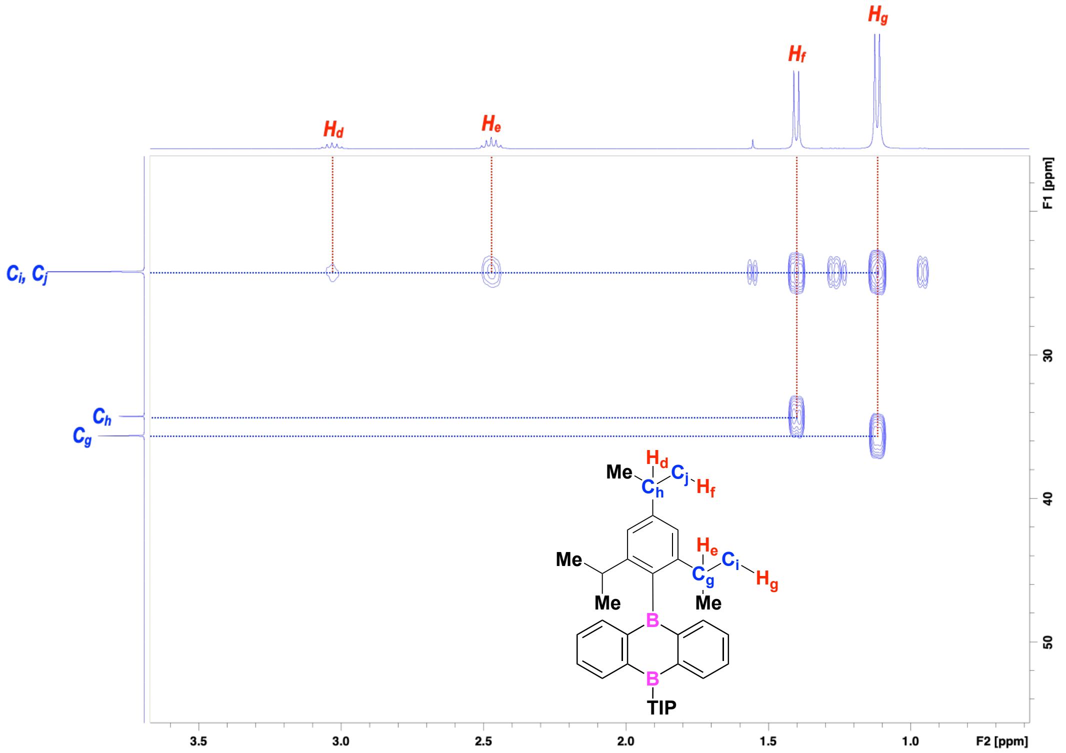

Figure S16(2, continued). Partial HMBC correlations of **B_2-Ant** (^{1}H: 400 MHz, ^{13}C: 100 MHz) in $CDCl_3$ at 25 °C.

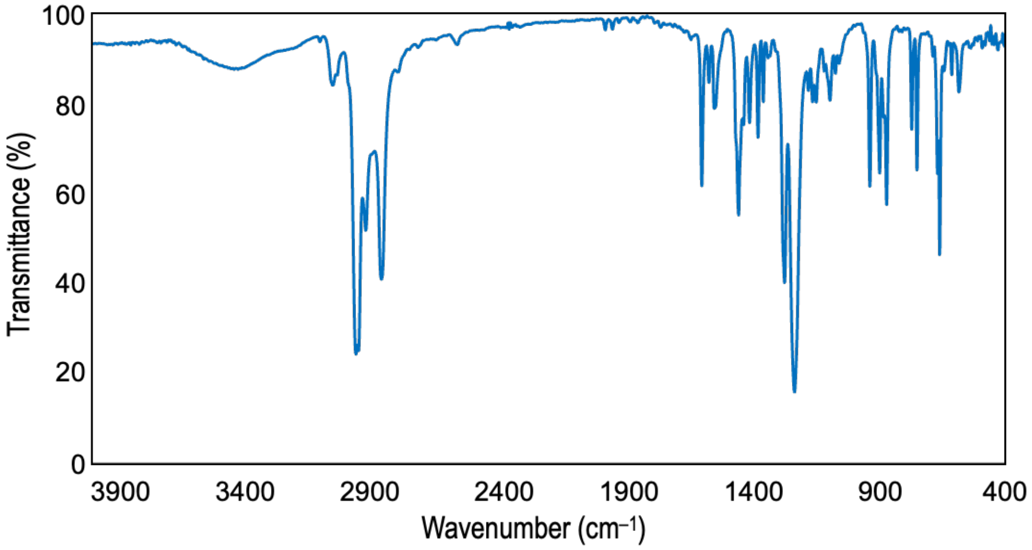

Figure S17. FT-IR spectrum of **B_2-Ant** (KBr) at 25 °C

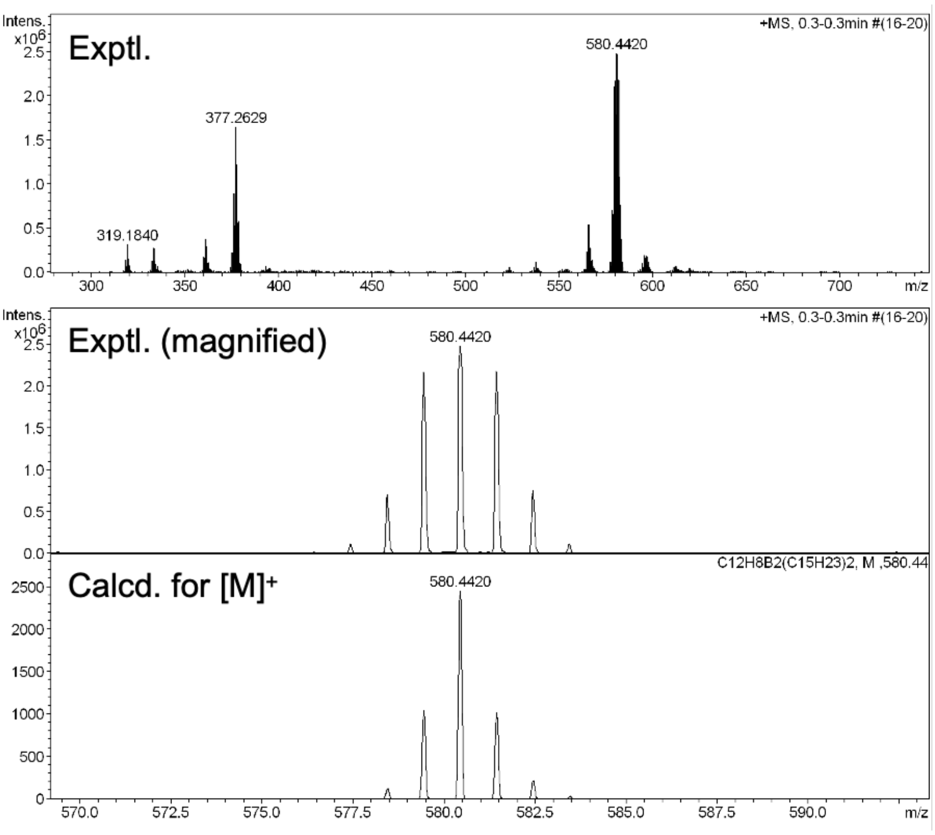

Figure S18. High-resolution APCI-TOF mass spectrum of **B_2-Ant**.

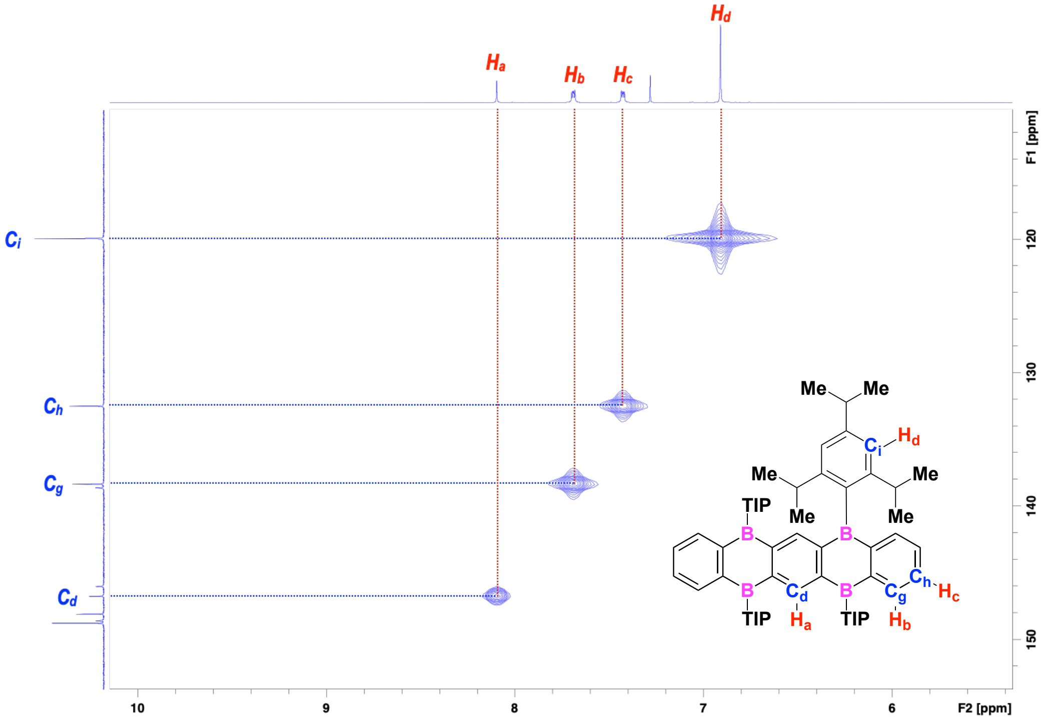

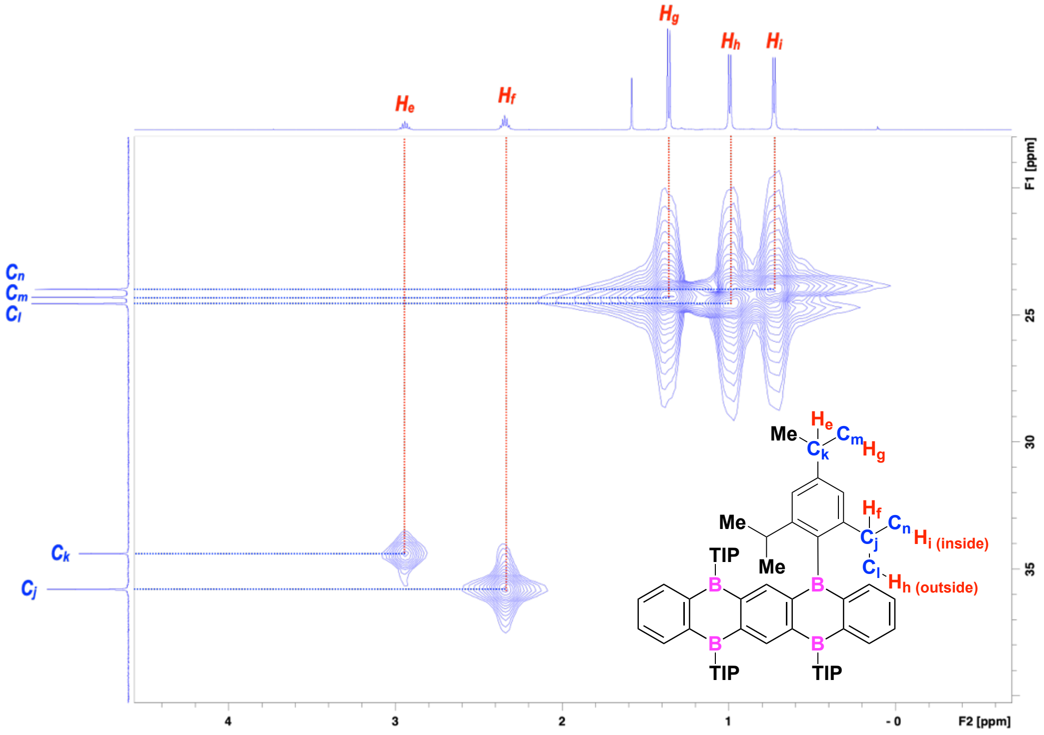

Figure S19. Partial HMQC correlations of **B$_4$-pent** (^{1}H: 400 MHz, ^{13}C: 100 MHz) in $CDCl_3$ at 25 °C.

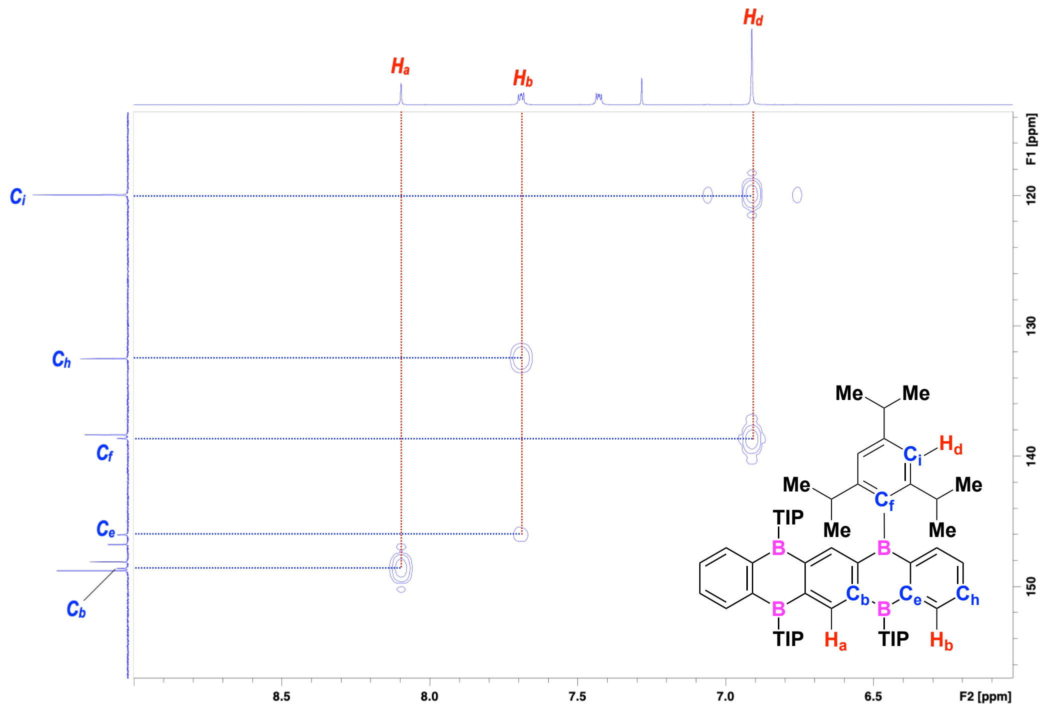

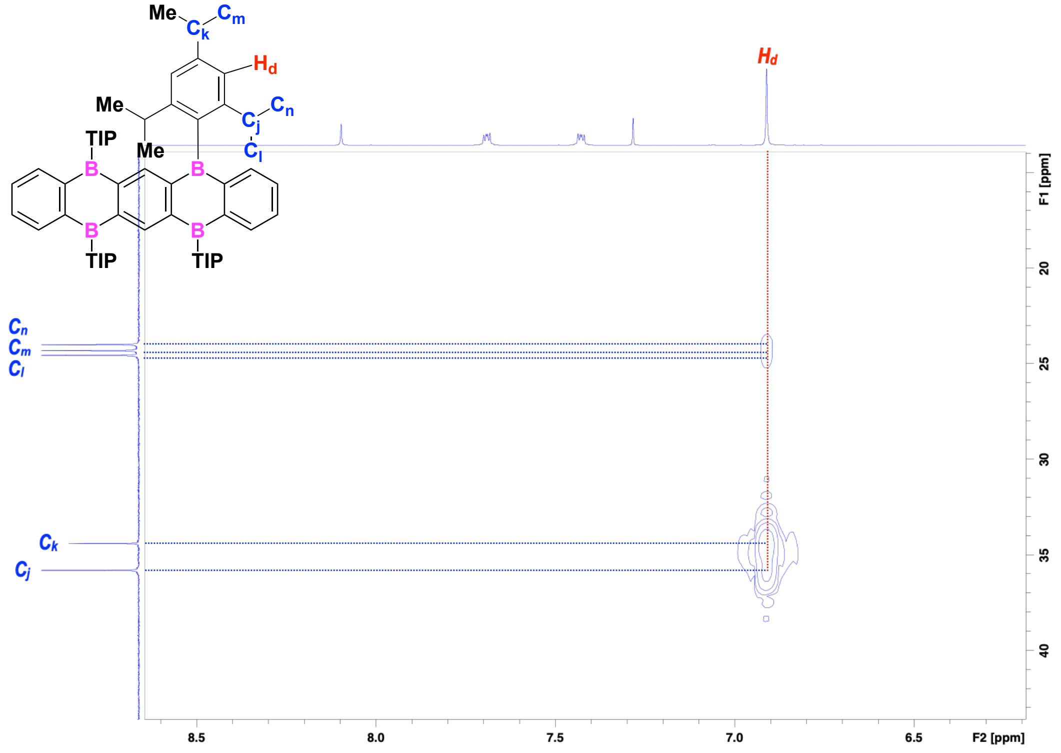

Figure S20(1). Partial HMBC correlations of **B_4-pent** (^{1}H: 400 MHz, ^{13}C: 100 MHz) in $CDCl_3$ at 25 °C.

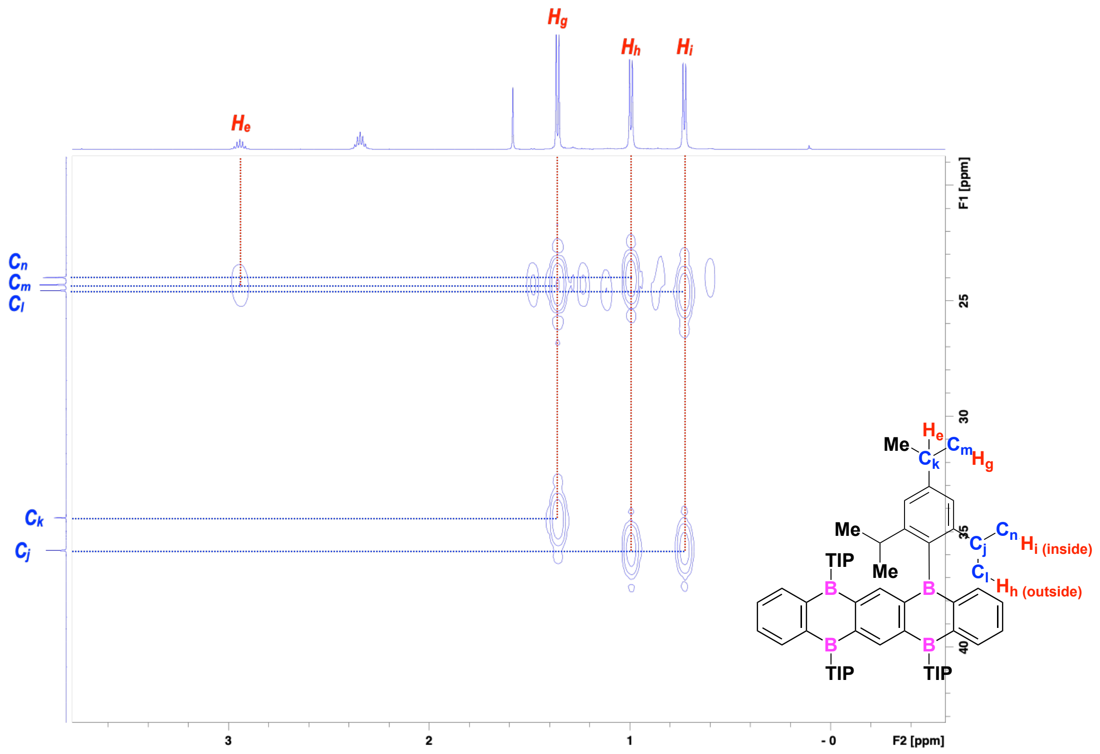

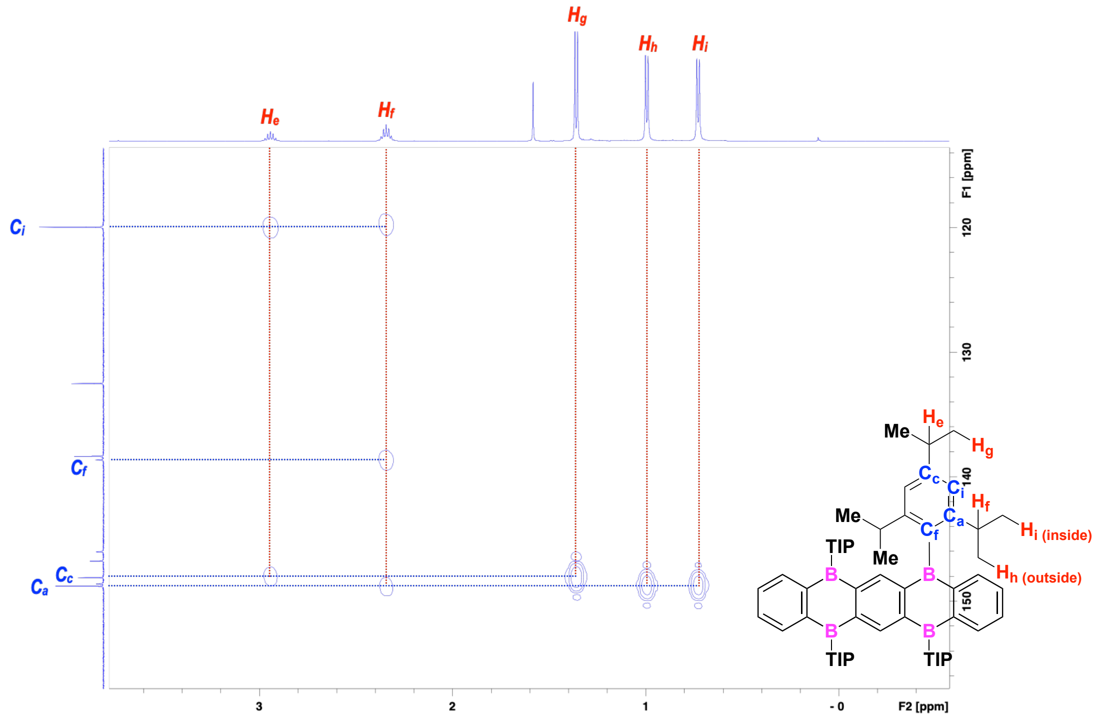

Figure S20(2, continued). Partial HMBC correlations of **B$_4$-pent** (^{1}H: 400 MHz, ^{13}C: 100 MHz) in $CDCl_3$ at 25 °C.

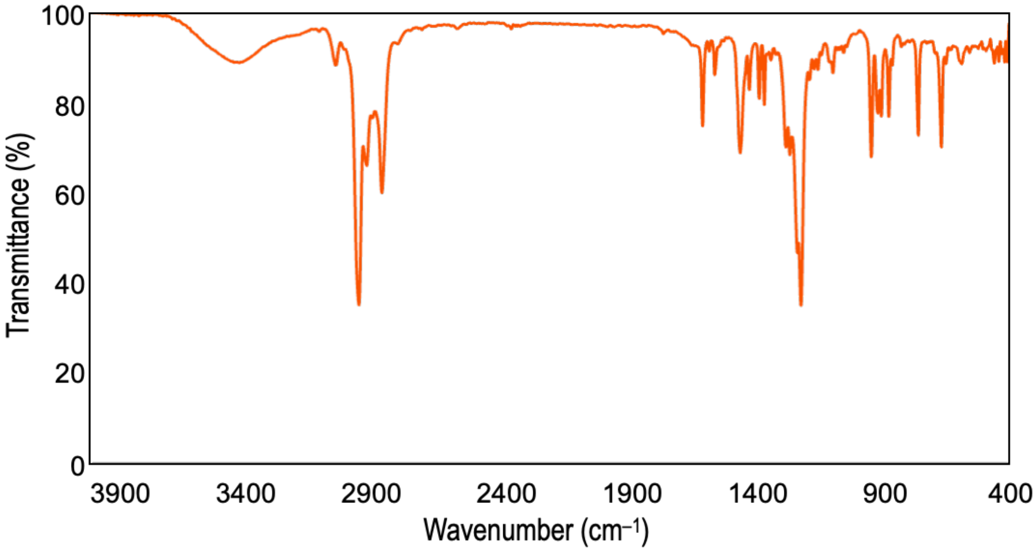

Figure S21. FT-IR spectrum of **B$_4$-pent** (KBr) at 25 °C.

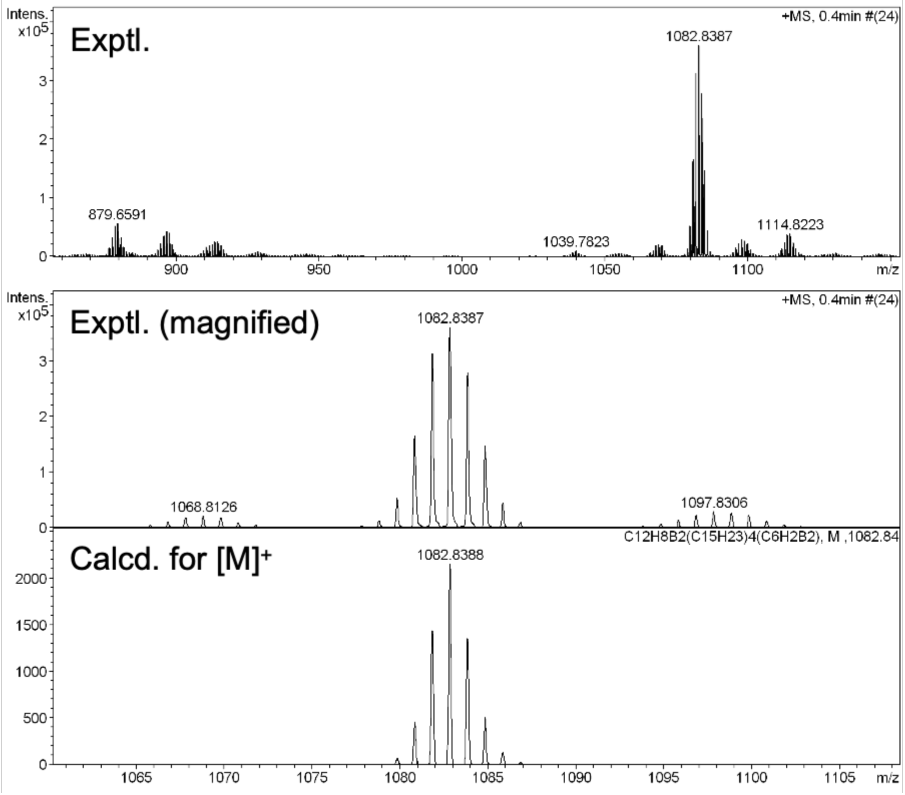

Figure S22. High-resolution APCI-TOF mass spectrum of **B$_4$-pent.**

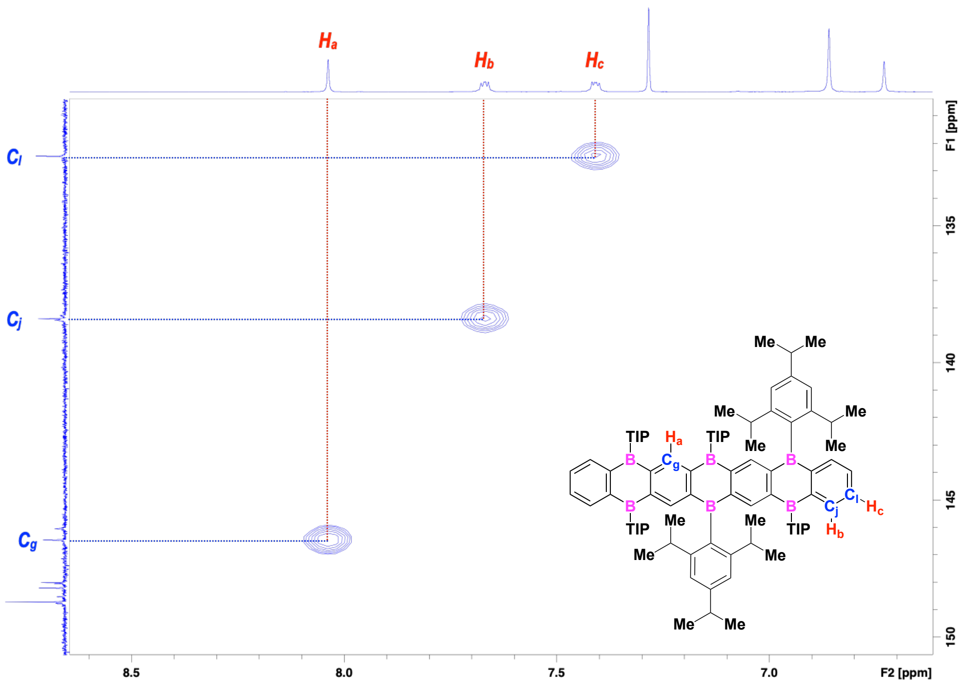

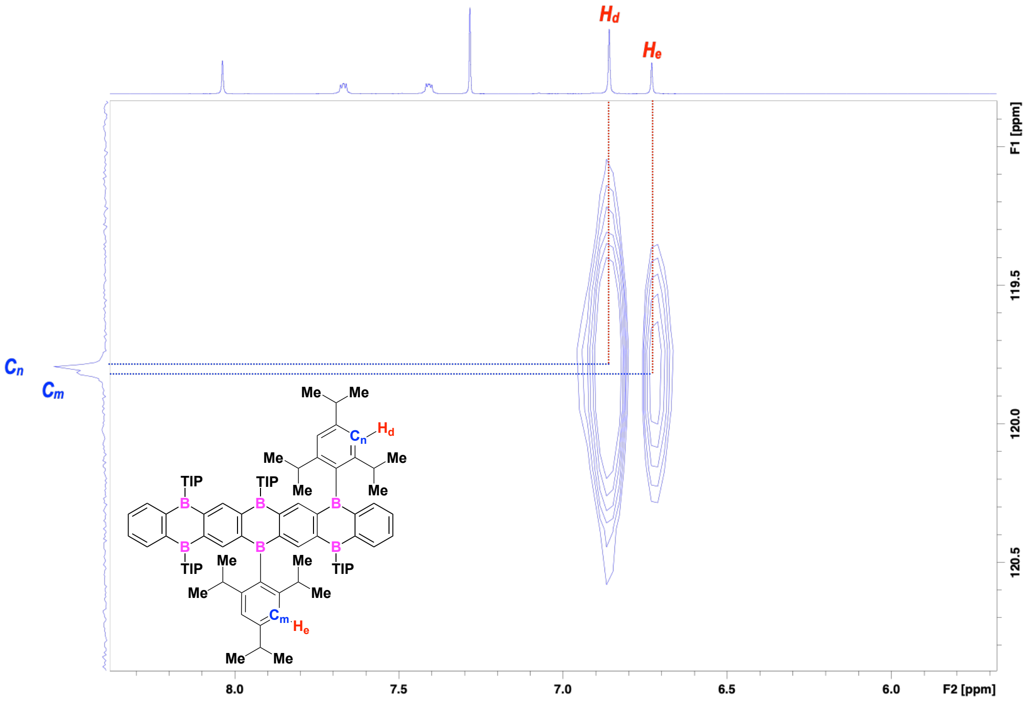

Figure S23(1). Partial HMQC correlation of **B$_6$-hept** (^{1}H: 500 MHz, ^{13}C: 126 MHz) in $CDCl_3$ at 25 °C.

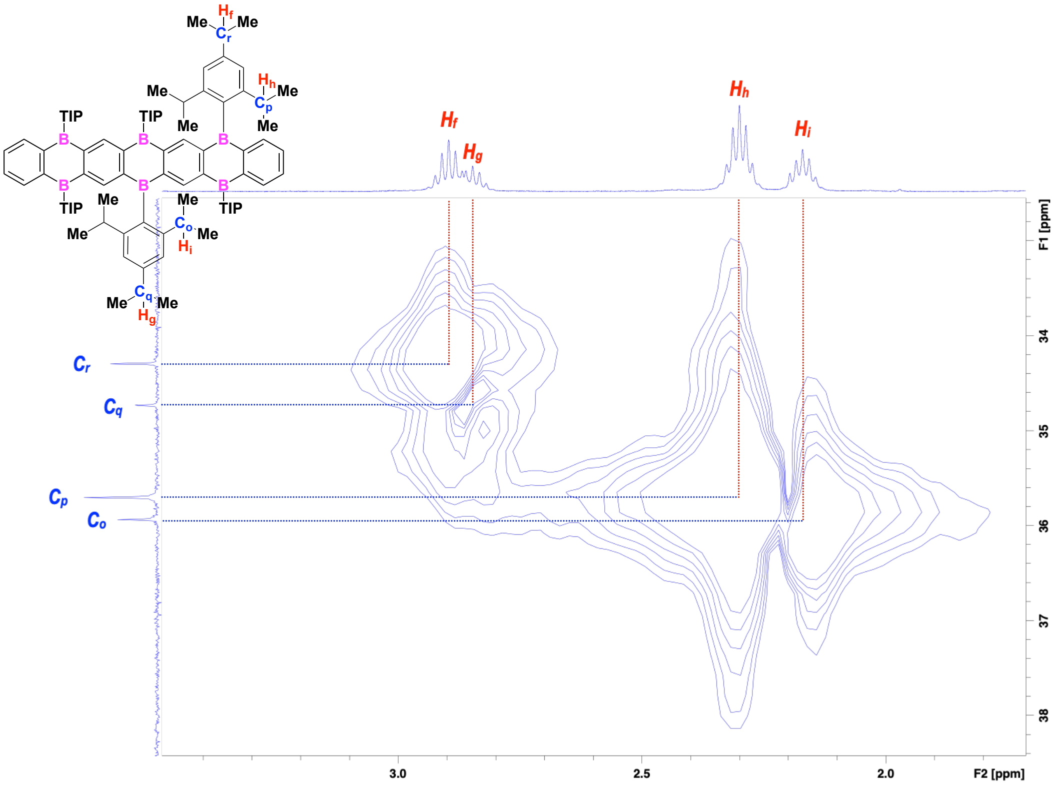

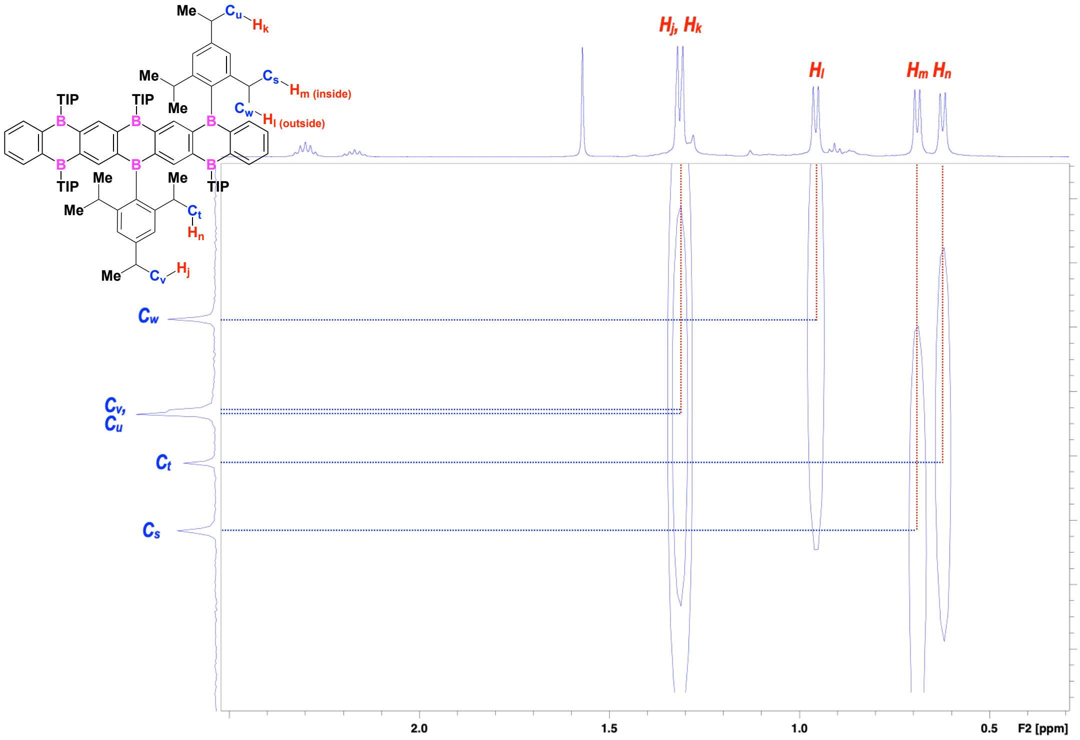

Figure S23(2, continued). Partial HMQC correlation of **B_6-hept** (^{1}H: 500 MHz, ^{13}C: 126 MHz) in $CDCl_3$ at 25 °C.

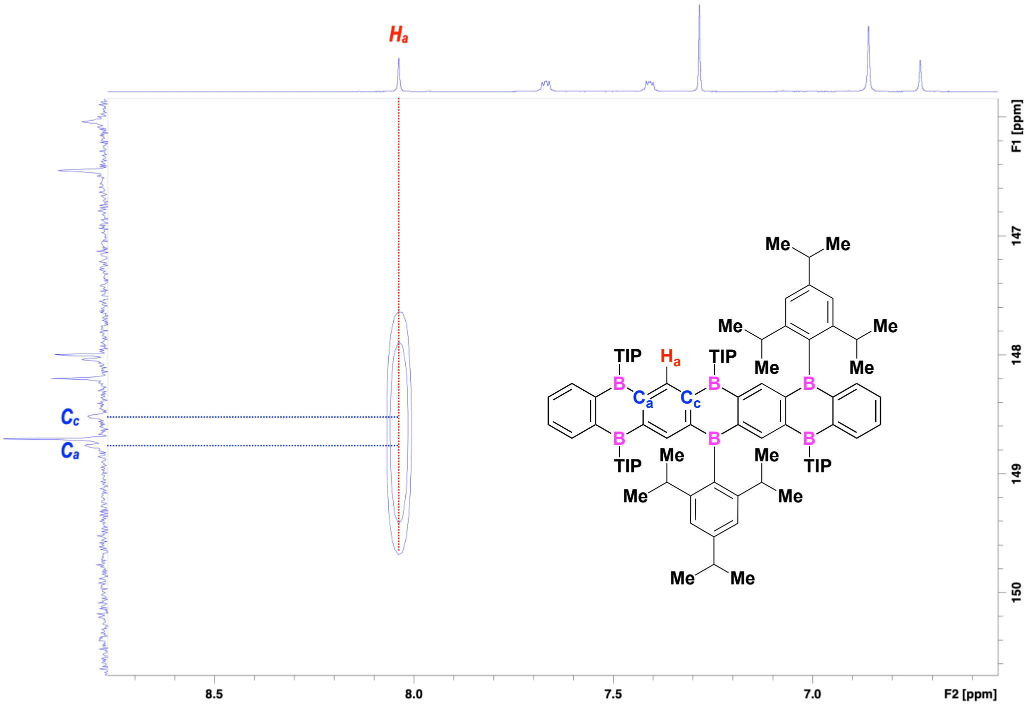

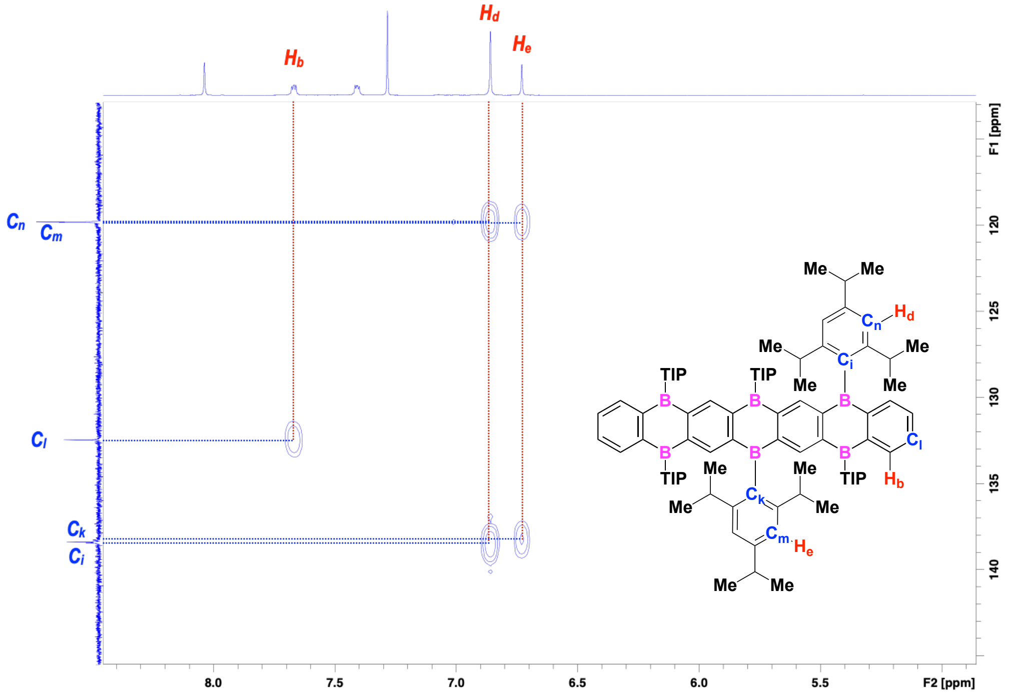

Figure S24(1). Partial HMBC correlations of **B_6-hept** (^{1}H: 500 MHz, ^{13}C: 126 MHz) in $CDCl_3$ at 25 °C.

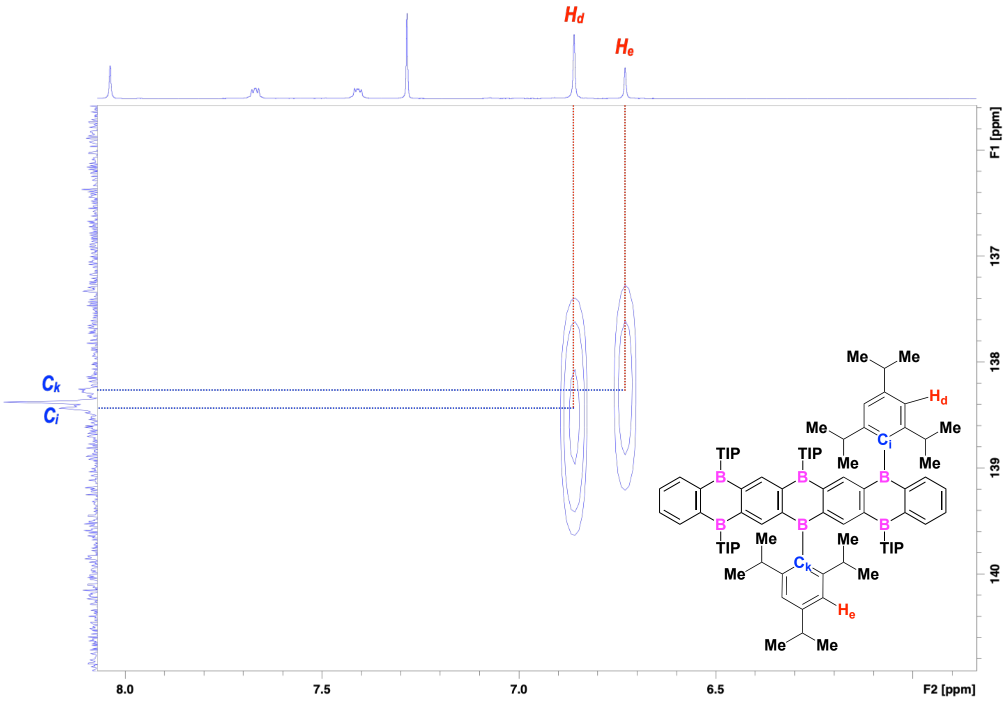

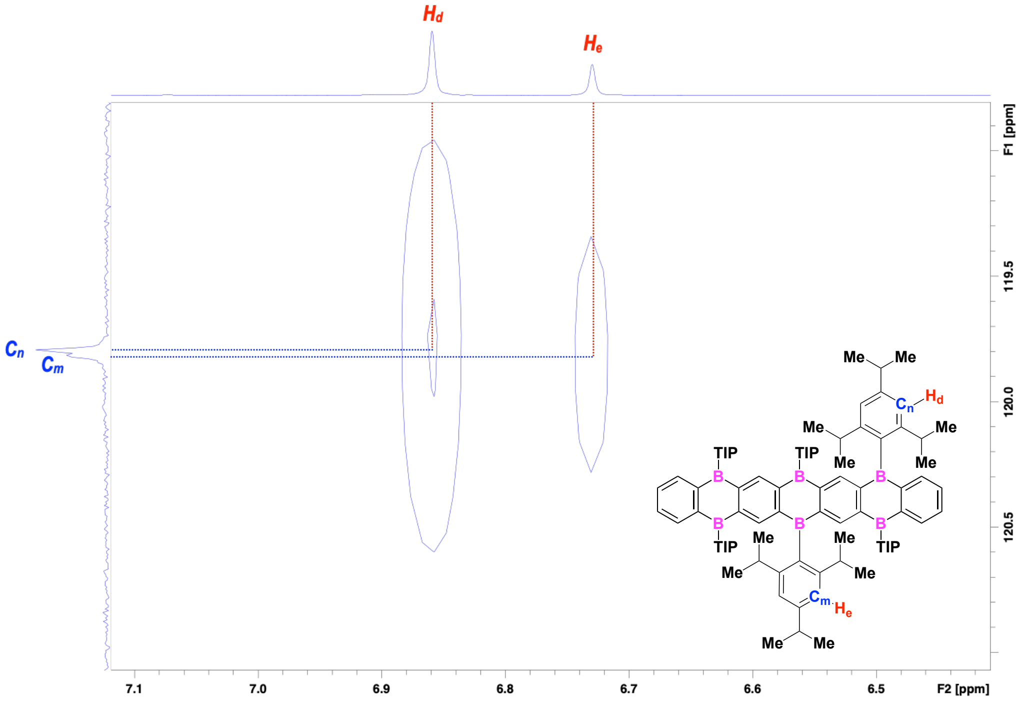

Figure S24(2, continued). Partial HMBC correlations of **B_6-hept** (^{1}H: 500 MHz, ^{13}C: 126 MHz) in $CDCl_3$ at 25 °C.

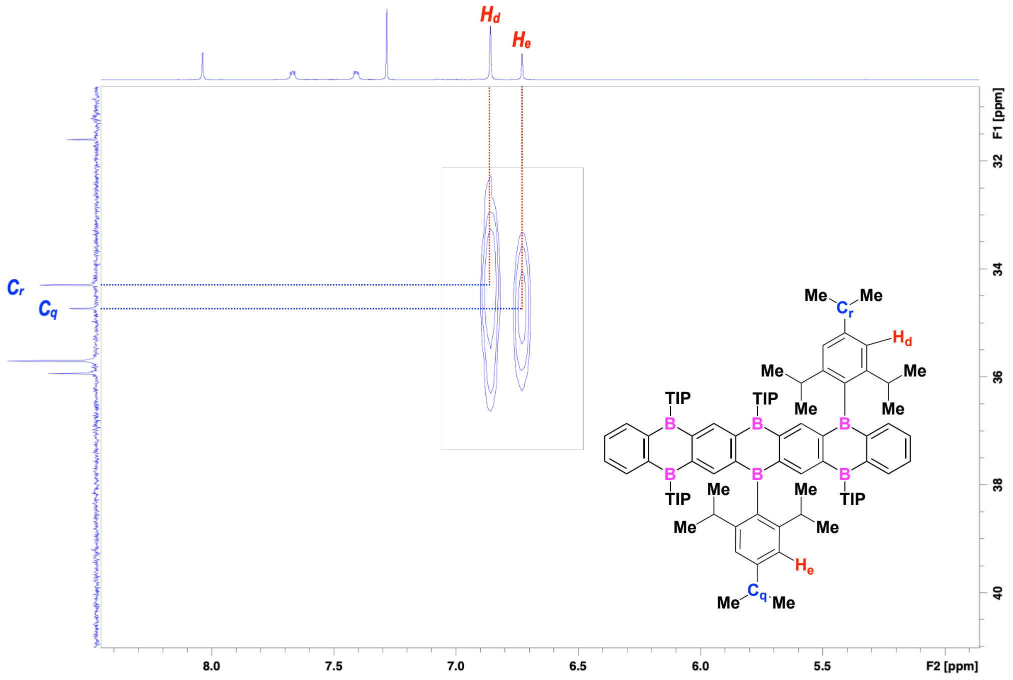

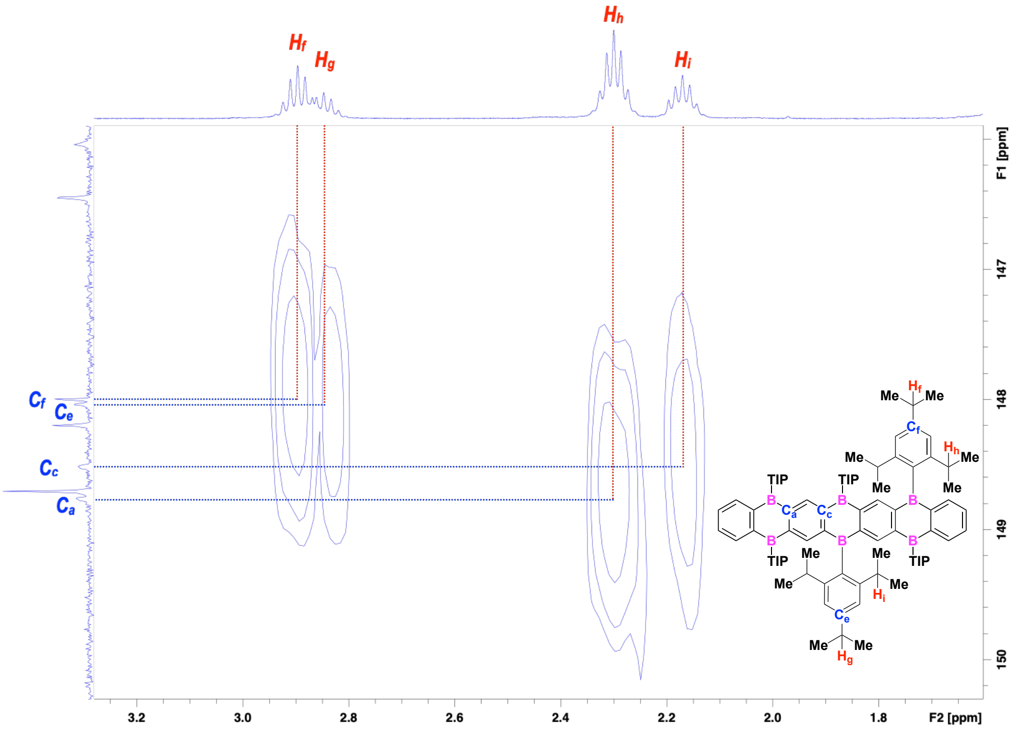

Figure S24(3, continued). Partial HMBC correlations of **B_6-hept** (^{1}H: 500 MHz, ^{13}C: 126 MHz) in $CDCl_3$ at 25 °C.

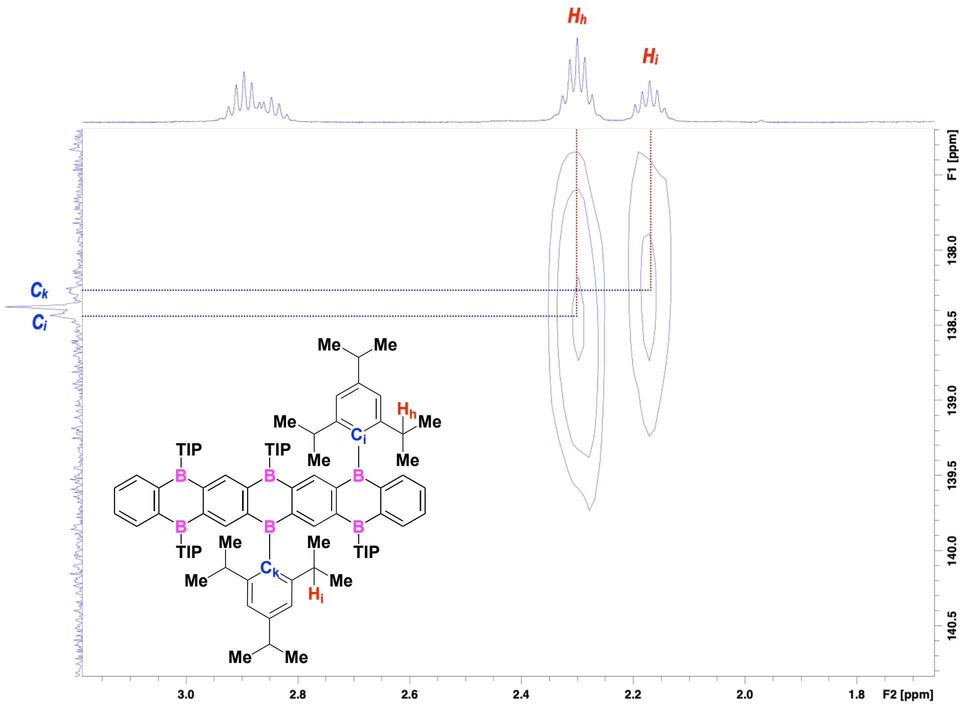

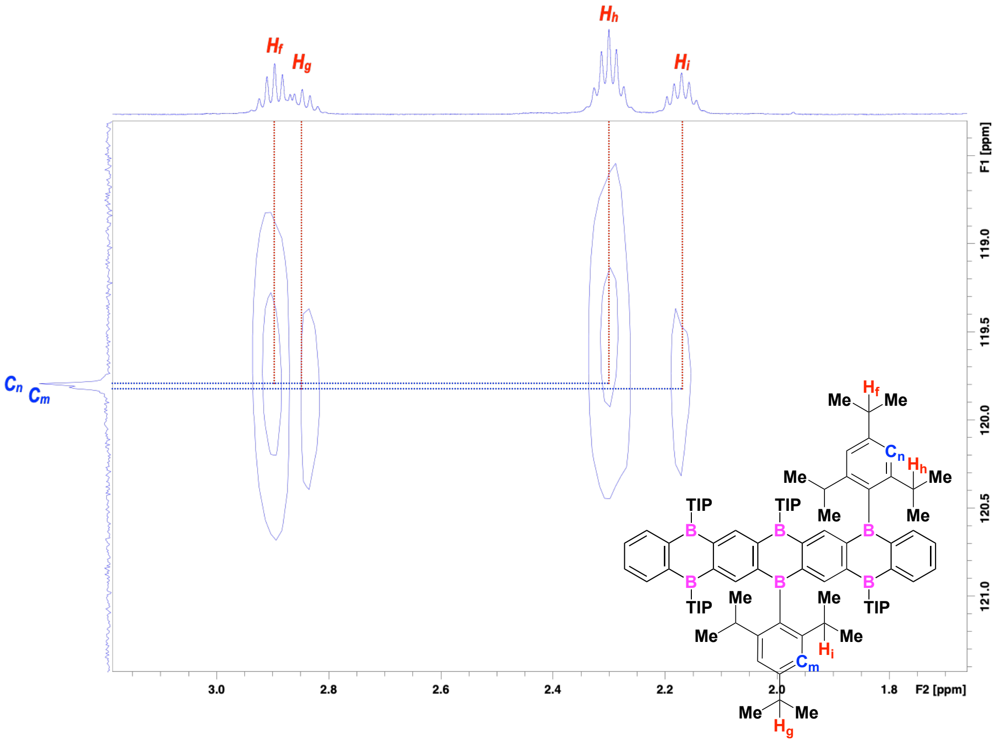

Figure S24(4, continued). Partial HMBC correlations of **B_6-hept** (^{1}H: 500 MHz, ^{13}C: 126 MHz) in $CDCl_3$ at 25 °C.

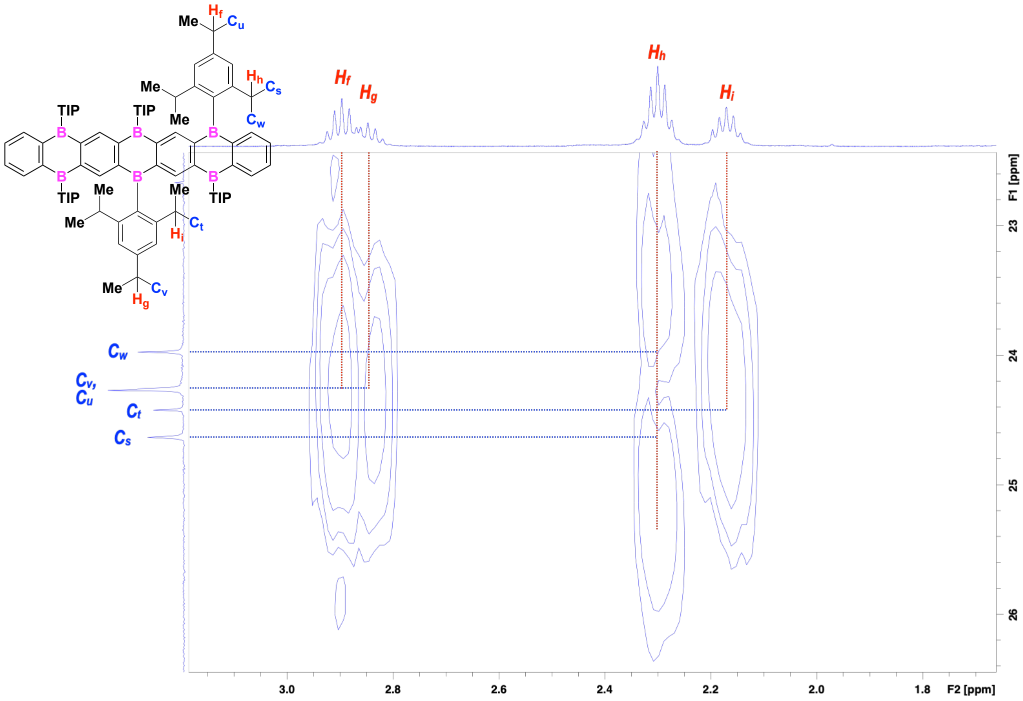

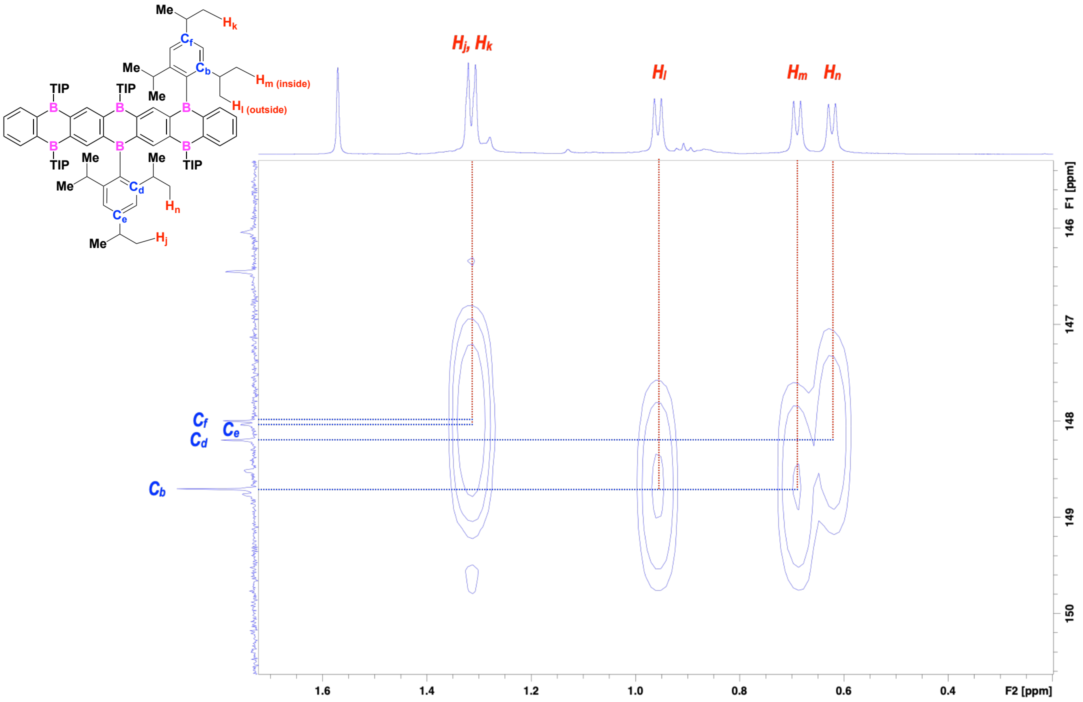

Figure S24(5, continued). Partial HMBC correlations of **B$_6$-hept** (^{1}H: 500 MHz, ^{13}C: 126 MHz) in $CDCl_3$ at 25 °C.

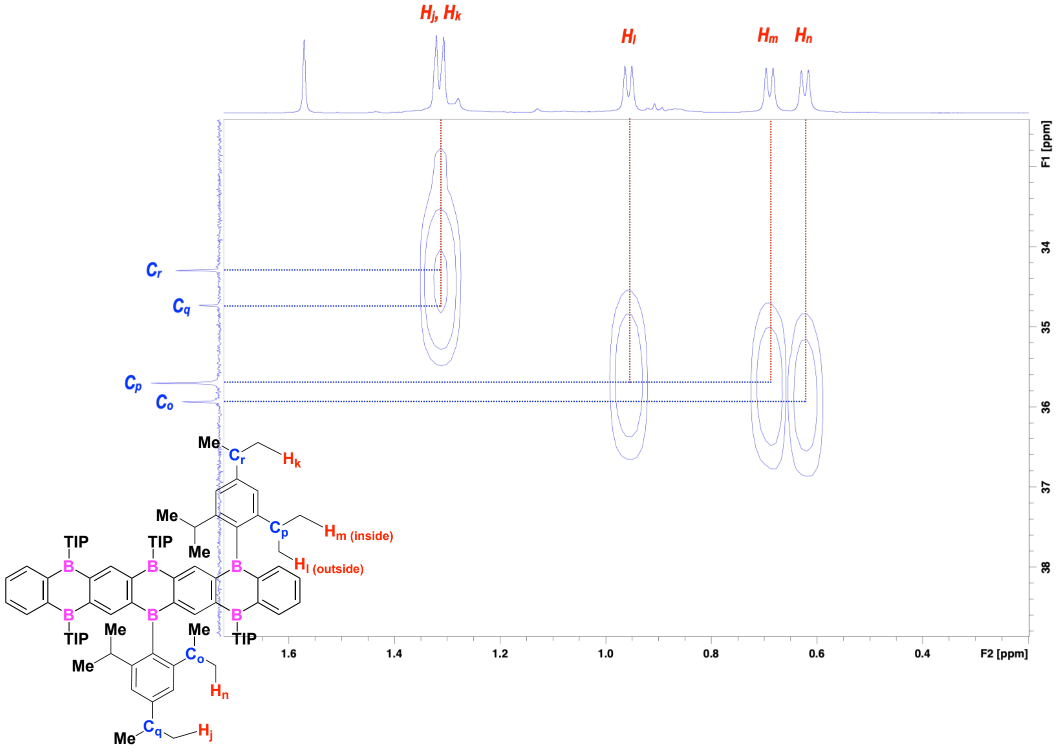

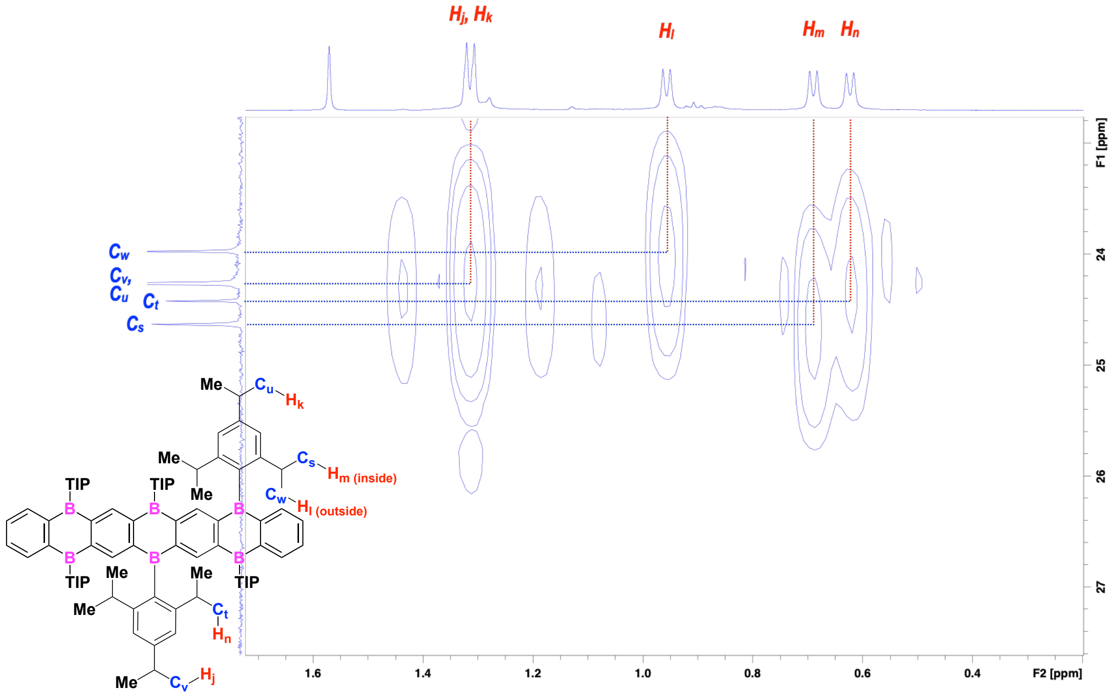

Figure S24(6, continued). Partial HMBC correlations of **B_6-hept** (^1H: 500 MHz, ^{13}C: 126 MHz) in $CDCl_3$ at 25 °C.

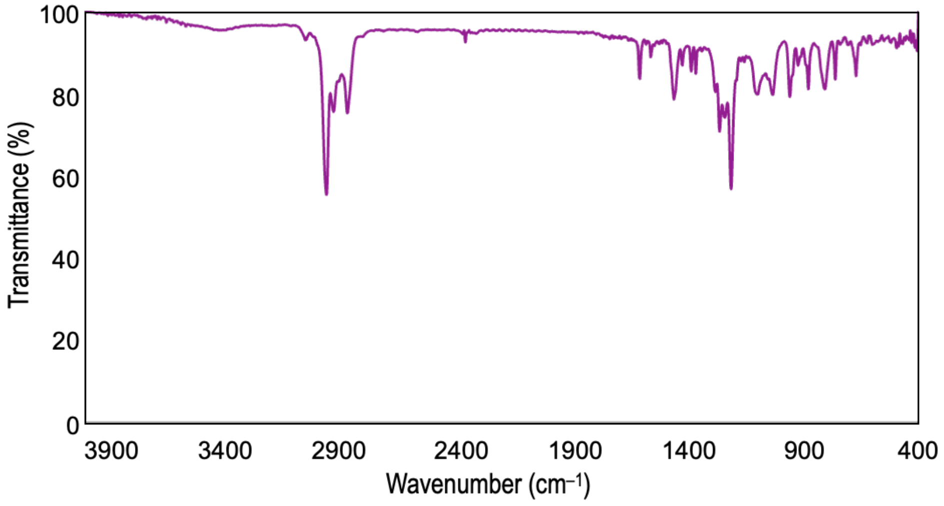

Figure S25. FT-IR spectrum of **B_6-hept** (KBr) at 25 °C.

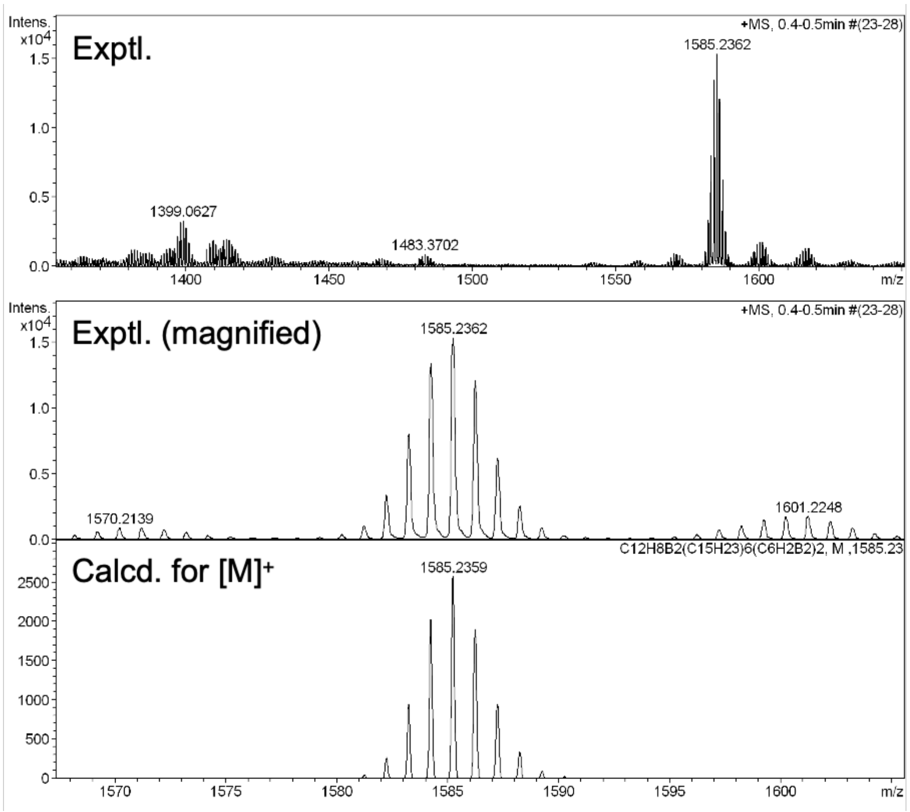

Figure S26. High-resolution APCI-TOF mass spectrum of **B_6-hept**.

8. Optimized Cartesian Coordinates (in Å) and Energies

Table S2. B_2-ant in vacuum

Energies [B3LYP-D3(BJ)/6-31G(d)]

Electronic Energy (EE)	-1683.038759	Hartree
Zero-point Energy Correction	0.863134	Hartree
Thermal Correction to Energy	0.908781	Hartree
Thermal Correction to Enthalpy	0.909725	Hartree
Thermal Correction to Free Energy	0.783858	Hartree

Cartesian coordinates [B3LYP-D3(BJ)/6-31G(d)]

C	0.66096100	-0.96988700	-3.67543300
C	-0.72773400	-0.83369500	-3.70130200
C	-1.41758200	-0.55129600	-2.52119700
C	1.34869500	-0.81437700	-2.47085800
C	-0.73294700	0.17494400	1.33796300
C	0.68558800	0.08932400	1.34884800
C	-1.40238700	0.47606300	2.53343900
C	1.37449400	0.35633800	2.54291600
C	-0.74386700	-0.40199800	-1.29894900
C	0.67074400	-0.52835600	-1.27536400
B	-1.52884100	-0.05185900	0.00852100
B	1.46312800	-0.30211200	0.05292300
C	0.69222400	0.67200800	3.71929000
C	-0.70229000	0.71915000	3.71698200
C	-3.09547200	0.10714800	-0.02040600
C	5.86312500	-0.13596200	-0.08376800
C	5.23672200	-1.32582000	0.28466200
C	3.84330200	-1.44964700	0.36438300
C	3.03533400	-0.33805300	0.04864200
C	3.66160800	0.87469500	-0.32087100
C	5.05241400	0.96305300	-0.38041700
C	7.37710700	-0.04131000	-0.15919700
C	7.85293900	0.27517400	-1.58694800
C	7.92854600	0.98387100	0.84582900
C	2.81369800	2.10341800	-0.63844300
C	3.03560300	3.22029600	0.39308000
C	3.03037600	2.59909600	-2.07559900
C	3.29871100	-2.78812600	0.85710000
C	3.25734400	-2.82039900	2.39756900
C	1.93742000	-3.19444900	0.28368200
C	-3.94868000	-1.02126500	0.11302000
C	-5.33675000	-0.85245500	0.09003400
C	-5.93567300	0.39481400	-0.08041800

C	-5.09228600	1.49245900	-0.21963300
C	-3.69601200	1.38223500	-0.18478900
C	-2.94848400	2.71800700	-0.28362000
C	-2.82407500	3.38438900	1.10100000
C	-1.58862300	2.71141300	-0.99145100
C	-7.44528700	0.55396500	-0.11391000
C	-8.06797500	-0.21142800	-1.29350800
C	-8.08667500	0.13105600	1.21825600
C	-3.48048900	-2.47580400	0.23455800
C	-3.42643100	-3.15533200	-1.14811300
C	-2.17413600	-2.72234300	0.99641400
H	1.20504200	-1.18996300	-4.59028100
H	-1.27003600	-0.94512000	-4.63662500
H	-2.49733900	-0.43434100	-2.54577300
H	2.43083300	-0.91067500	-2.45283300
H	-2.48780800	0.52199500	2.53278600
H	2.45994900	0.31260500	2.54586400
H	1.24421000	0.87389500	4.63348100
H	-1.24201400	0.95231900	4.63113800
H	5.85325000	-2.19182500	0.52148300
H	5.51410800	1.90498200	-0.66633000
H	7.77946500	-1.02577200	0.11516700
H	7.49019200	1.25658400	-1.91407500
H	7.48363900	-0.47076700	-2.29869600
H	8.94803900	0.28908800	-1.63857100
H	7.56882900	1.99345600	0.61543000
H	9.02440600	1.00507000	0.81906000
H	7.61307700	0.74258300	1.86644500
H	1.75562600	1.81601200	-0.56507400
H	4.07558200	3.56632100	0.38373800
H	2.80748400	2.86503800	1.40352600
H	2.39101800	4.08065600	0.17803800
H	4.06823800	2.91241700	-2.23658800
H	2.38292000	3.45735200	-2.28993300
H	2.80020000	1.80826000	-2.79698300
H	4.02208200	-3.55117000	0.53827400
H	2.52274100	-2.10414000	2.77951200
H	4.23323000	-2.56419000	2.82332600
H	2.97575500	-3.81773200	2.75656800
H	1.13148200	-2.54914600	0.65457900
H	1.68764300	-4.21379800	0.60013500
H	1.92210500	-3.15867700	-0.80886200
H	-5.96483300	-1.73398600	0.20196600
H	-5.53374400	2.47877400	-0.35409200
H	-3.59990600	3.36691500	-0.88424600

H	-2.13867900	2.82630900	1.74491200
H	-3.79644900	3.43404600	1.60231300
H	-2.43670100	4.40554900	1.00034300
H	-0.81717900	2.20319100	-0.40376000
H	-1.24678900	3.74440700	-1.12726700
H	-1.63582300	2.23447300	-1.97350200
H	-7.65657500	1.62173900	-0.26111000
H	-7.89921100	-1.29013100	-1.19552300
H	-7.63082700	0.10951800	-2.24497700
H	-9.15074600	-0.04492400	-1.33755600
H	-7.91766600	-0.93393200	1.41544800
H	-9.16976800	0.30022400	1.19980300
H	-7.66356100	0.69646400	2.05532600
H	-4.26831500	-2.98683300	0.80412900
H	-2.62191800	-2.73674200	-1.75946100
H	-4.36906300	-3.02086600	-1.68911900
H	-3.24268600	-4.23108400	-1.03903400
H	-1.29921800	-2.36853300	0.44063700
H	-2.03387900	-3.80008600	1.14088200
H	-2.17047200	-2.24143600	1.97790300

Table S3. B_4-Pent (bent-zigzag) in vacuum

Energies [B3LYP-D3(BJ)/6-31G(d)]

Electronic Energy (EE)	-3133.855576	Hartree
Zero-point Energy Correction	1.623061	Hartree
Thermal Correction to Energy	1.711840	Hartree
Thermal Correction to Enthalpy	1.712784	Hartree
Thermal Correction to Free Energy	1.49177	Hartree

Single-point energy [ωB97XD/6-311G(d,p)//B3LYP-D3(BJ)/6-31G(d)]

E(RωB97XD)	-3133.200014	Hartree

Cartesian coordinates [B3LYP-D3(BJ)/6-31G(d)]

C	-0.69779600	5.89042700	2.35782600
C	0.69760200	5.89048300	2.35776200
C	1.39263600	4.80269700	1.82277800
C	-1.39279300	4.80259400	1.82288800
C	0.70994200	1.17866400	0.34762700
C	-0.70996200	1.17864100	0.34761900
C	1.38540900	-0.00001600	0.00005900
C	-1.38538800	-0.00006200	0.00006400
C	0.71165400	-3.68751600	-1.31268100
C	-0.71154100	-3.68753100	-1.31270000
C	1.39279400	-4.80267400	-1.82276500

C	0.69780000	-5.89047800	-2.35776400
C	-0.69759900	-5.89048500	-2.35779600
C	-1.39263700	-4.80269600	-1.82282200
C	0.71154000	3.68749500	1.31274000
C	-0.71165400	3.68744600	1.31278300
B	1.48791200	2.48890600	0.68608900
B	-1.48797800	2.48884900	0.68608300
C	-0.70992300	-1.17874100	-0.34749500
B	-1.48790300	-2.48896600	-0.68598000
C	0.70998200	-1.17871600	-0.34750600
B	1.48799200	-2.48892300	-0.68601000
C	-3.00078800	2.62957800	0.29565300
C	-3.00072900	-2.62971100	-0.29562100
C	3.00081900	-2.62961400	-0.29562100
C	3.00072300	2.62965500	0.29566400
C	3.31341200	3.29853200	-0.90324900
C	4.64953400	3.43648400	-1.29021200
C	5.69048100	2.92035800	-0.51517700
C	5.36573100	2.24960000	0.66868500
C	4.04026800	2.09384600	1.08074400
C	4.04032200	-2.09380300	-1.08074900
C	5.36580400	-2.24952200	-0.66874100
C	5.69061300	-2.92025900	0.51511700
C	4.64970300	-3.43638900	1.29020200
C	3.31356300	-3.29846000	0.90329400
C	-3.31349900	-3.29859200	0.90327000
C	-4.64964300	-3.43652600	1.29015700
C	-5.69054200	-2.92036200	0.51508200
C	-5.36571300	-2.24956200	-0.66873500
C	-4.04022300	-2.09383100	-1.08072400
C	-3.31347300	3.29842900	-0.90327600
C	-4.64959600	3.43642900	-1.29021800
C	-5.69055300	2.92034600	-0.51516800
C	-5.36580400	2.24955000	0.66867500
C	-4.04034000	2.09376800	1.08072400
C	-7.13745600	3.08712700	-0.94712600
C	-7.83961800	1.73192400	-1.13247000
C	-7.91403500	3.97757600	0.03792000
C	-7.13746600	-3.08711000	0.94697900
C	-7.91390300	-3.97780700	-0.03796000
C	-7.83974000	-1.73192700	1.13200300
C	-2.19160800	-3.81786900	1.79518800
C	-2.11717600	-3.03311500	3.11442500
C	-2.28552800	-5.33167800	2.03055300
C	2.19164700	-3.81778400	1.79515200

C	2.28543800	-5.33164800	2.03021600
C	2.11731000	-3.03327800	3.11454000
C	7.13753500	-3.08697200	0.94703300
C	7.91399400	-3.97770100	-0.03786000
C	7.83978800	-1.73176700	1.13200200
C	3.71459600	-1.39155700	-2.39260000
C	4.42317700	-0.03723500	-2.53154400
C	4.01493900	-2.30654700	-3.59213000
C	3.71461100	1.39160300	2.39261300
C	4.42333000	0.03735900	2.53162200
C	4.01484900	2.30667200	3.59211200
C	7.13738500	3.08710400	-0.94714600
C	7.91385600	3.97786500	0.03770900
C	7.83966700	1.73191600	-1.13212200
C	2.19145000	3.81779800	-1.79508600
C	2.28532600	5.33160800	-2.03044900
C	2.11693200	3.03304200	-3.11431700
C	-3.71468900	1.39150100	2.39258100
C	-4.01489000	2.30658400	3.59208300
C	-4.42342700	0.03727100	2.53160700
C	-2.19153900	3.81774000	-1.79512100
C	-2.11752500	3.03364800	-3.11477000
C	-2.28504000	5.33170200	-2.02969200
C	-3.71448900	-1.39157100	-2.39256400
C	-4.01457100	-2.30668400	-3.59207000
C	-4.42324900	-0.03736100	-2.53168500
H	-1.24220200	6.74018500	2.76135300
H	1.24197800	6.74029000	2.76122700
H	2.47888000	4.81831900	1.79275300
H	-2.47904000	4.81813900	1.79295200
H	2.47121300	0.00000000	0.00005500
H	-2.47119300	-0.00007900	0.00006100
H	2.47903900	-4.81824200	-1.79276400
H	1.24220500	-6.74024700	-2.76126900
H	-1.24197500	-6.74025800	-2.76133300
H	-2.47888300	-4.81829300	-1.79286500
H	4.89140400	3.94439400	-2.22152300
H	6.16406400	1.83843500	1.28048100
H	6.16410400	-1.83834900	-1.28057700
H	4.89161800	-3.94428000	2.22151200
H	-4.89157100	-3.94443800	2.22145200
H	-6.16400000	-1.83835200	-1.28055800
H	-4.89146000	3.94435500	-2.22152100
H	-6.16413600	1.83838400	1.28047100
H	-7.13057500	3.59476200	-1.92103300

H	-7.89013900	1.18169400	-0.18646700
H	-7.30229000	1.10662600	-1.85272500
H	-8.86547900	1.87168200	-1.49308900
H	-7.96040100	3.51445700	1.03049000
H	-8.94268400	4.13646000	-0.30679400
H	-7.43217900	4.95487900	0.14806800
H	-7.13062700	-3.59454800	1.92099000
H	-7.96017700	-3.51488800	-1.03062800
H	-8.94258100	-4.13667900	0.30667500
H	-7.43197900	-4.95510500	-0.14785900
H	-7.89028000	-1.18189100	0.18588800
H	-7.30249700	-1.10643300	1.85215600
H	-8.86560300	-1.87170000	1.49261000
H	-1.24140500	-3.64210800	1.27238200
H	-3.03757700	-3.15211500	3.69765000
H	-1.27886400	-3.38391400	3.72814700
H	-1.97789400	-1.96450300	2.92091400
H	-3.20866600	-5.59756300	2.55828700
H	-1.44051800	-5.68184200	2.63538700
H	-2.27223700	-5.87232700	1.07832800
H	1.24144300	-3.64183100	1.27240400
H	3.20857600	-5.59771800	2.55785700
H	2.27206400	-5.87210800	1.07788400
H	1.44042200	-5.68185900	2.63501500
H	3.03772500	-3.15244200	3.69770800
H	1.27900000	-3.38414300	3.72822400
H	1.97808300	-1.96462200	2.92123500
H	7.13069700	-3.59437200	1.92106400
H	7.96028000	-3.51482400	-1.03054600
H	7.43208400	-4.95500800	-0.14772300
H	8.94266900	-4.13654400	0.30679700
H	7.89031200	-1.18176300	0.18586700
H	8.86565400	-1.87150800	1.49261300
H	7.30253700	-1.10625600	1.85213300
H	2.63344800	-1.19195400	-2.39895900
H	5.50829200	-0.16351200	-2.61836500
H	4.23161400	0.60809700	-1.67065100
H	4.08129300	0.48488100	-3.43198400
H	5.08084500	-2.56127400	-3.62628200
H	3.75311000	-1.81253000	-4.53535100
H	3.44726900	-3.24084200	-3.52635900
H	2.63348400	1.19188600	2.39897100
H	5.50842800	0.16374800	2.61849100
H	4.23186800	-0.60801600	1.67073900
H	4.08146400	-0.48476600	3.43206400

H	5.08073100	2.56150100	3.62625700
H	3.75306300	1.81266200	4.53534900
H	3.44708700	3.24090700	3.52630500
H	7.13049900	3.59449400	-1.92118100
H	7.96018400	3.51500000	1.03040000
H	7.43192700	4.95516300	0.14757600
H	8.94251500	4.13672700	-0.30698400
H	7.89023900	1.18192400	-0.18598300
H	8.86551700	1.87167600	-1.49277300
H	7.30240600	1.10638100	-1.85222400
H	1.24128700	3.64202000	-1.27220900
H	1.44026300	5.68176200	-2.63521400
H	2.27210200	5.87225000	-1.07821900
H	3.20841700	5.59751600	-2.55825400
H	1.97766000	1.96443200	-2.92079300
H	1.27858400	3.38384100	-3.72798700
H	3.03730200	3.15203700	-3.69759300
H	-2.63357100	1.19175500	2.39892900
H	-5.08076100	2.56145900	3.62622300
H	-3.44708800	3.24079600	3.52627900
H	-3.75312700	1.81256500	4.53532200
H	-5.50852100	0.16366700	2.61854500
H	-4.08151700	-0.48487100	3.43202000
H	-4.23201500	-0.60809100	1.67070000
H	-1.24131200	3.64140300	-1.27255000
H	-3.03795300	3.15322200	-3.69783200
H	-1.97853400	1.96489600	-2.92183300
H	-1.27918600	3.38451900	-3.72841300
H	-3.20821900	5.59812000	-2.55708700
H	-1.44005200	5.68192500	-2.63452400
H	-2.27138400	5.87185700	-1.07719300
H	-2.63337500	-1.19179700	-2.39882200
H	-5.08043800	-2.56156600	-3.62629800
H	-3.75272900	-1.81268100	-4.53529600
H	-3.44676800	-3.24089000	-3.52619900
H	-4.08126600	0.48475400	-3.43208700
H	-4.23192600	0.60804000	-1.67078800
H	-5.50833100	-0.16378300	-2.61873100

Table S4. B_4-Pent (twist) in vacuum

Energies [B3LYP-D3(BJ)/6-31G(d)]

Electronic Energy (EE)	-3133.853373	Hartree
Zero-point Energy Correction	1.623872	Hartree
Thermal Correction to Energy	1.712326	Hartree

Thermal Correction to Enthalpy	1.713271	Hartree
Thermal Correction to Free Energy	1.492174	Hartree

Single-point energy [ωB97XD/6-311G(d,p)/B3LYP-D3(BJ)/6-31G(d)]

E(RωB97XD)	-	-3133.197677	Hartree

Cartesian coordinates [B3LYP-D3(BJ)/6-31G(d)]

C	-0.71391900	6.34710400	0.30832200
C	0.66413000	6.33881500	0.52564400
C	1.36049900	5.12741300	0.52068100
C	-1.39021200	5.14392500	0.09007700
C	0.71091400	1.22774900	0.07210800
C	-0.70761300	1.22907500	0.00160000
C	1.38697700	0.00012300	-0.00044400
C	-1.38306300	-0.00004200	-0.00044300
C	0.69934800	-3.90893100	-0.30620100
C	-0.70886500	-3.91730200	-0.09261100
C	1.36108100	-5.12714000	-0.52180700
C	0.66484200	-6.33862000	-0.52687500
C	-0.71320200	-6.34708300	-0.30953900
C	-1.38962700	-5.14400000	-0.09116100
C	0.69890700	3.90910800	0.30516100
C	-0.70931000	3.91729900	0.09160300
B	1.48696300	2.56374900	0.29957500
B	-1.47901400	2.57928600	-0.13102500
C	-0.70746900	-1.22908100	-0.00252400
B	-1.47867600	-2.57938800	0.13024800
C	0.71105700	-1.22758000	-0.07304100
B	1.48725900	-2.56349200	-0.30042100
C	-2.98278100	2.57218100	-0.58079000
C	-2.98228600	-2.57250900	0.58055500
C	3.02187100	-2.49688200	-0.62446900
C	3.02147500	2.49725100	0.62411200
C	3.99346600	2.41167800	-0.39101000
C	5.34043300	2.28332400	-0.04239200
C	5.75139700	2.20751700	1.29055100
C	4.77570900	2.28532500	2.28944900
C	3.42289200	2.43843700	1.97327900
C	3.42389500	-2.43775400	-1.97344100
C	4.77683800	-2.28453400	-2.28896000
C	5.75208800	-2.20695400	-1.28959200
C	5.34053400	-2.28312000	0.04313300
C	3.99339200	-2.41156100	0.39109700
C	-3.28550900	-2.76384100	1.94234200
C	-4.61853500	-2.74257400	2.36286600

C	-5.66562200	-2.53478100	1.46234800
C	-5.35066100	-2.33648700	0.11405000
C	-4.02836200	-2.34601200	-0.33558300
C	-3.28650200	2.76347100	-1.94245900
C	-4.61966300	2.74188000	-2.36255000
C	-5.66639600	2.53379000	-1.46169600
C	-5.35094200	2.33557800	-0.11349700
C	-4.02850000	2.34542700	0.33569800
C	-7.10905900	2.53245100	-1.93750200
C	-7.82668900	1.21638200	-1.59746000
C	-7.87984700	3.73884500	-1.37421000
C	-7.10813900	-2.53373800	1.93860900
C	-7.87925500	-3.73960900	1.37467100
C	-7.82570100	-1.21731500	1.59976200
C	-2.15280700	-2.91159300	2.95072100
C	-1.94128900	-1.60077800	3.72796200
C	-2.34170700	-4.10699800	3.89429200
C	3.56593600	-2.50288600	1.85027400
C	3.62528200	-3.95855800	2.34732700
C	4.36619800	-1.57678700	2.77554200
C	7.22001700	-2.00224900	-1.62429900
C	7.74377200	-3.03502300	-2.63381800
C	7.47269700	-0.56693700	-2.11762800
C	2.37283700	-2.49907200	-3.07666200
C	2.21441800	-1.14606400	-3.78764700
C	2.64577300	-3.63544000	-4.07282200
C	2.37135800	2.49982500	3.07604300
C	2.21262300	1.14682700	3.78699600
C	2.64382200	3.63621500	4.07230400
C	7.21918000	2.00279300	1.62590100
C	7.74239100	3.03501100	2.63626900
C	7.47167200	0.56720600	2.11851900
C	3.56663300	2.50267100	-1.85039700
C	3.62528100	3.95837200	-2.34746100
C	4.36790800	1.57711600	-2.77532800
C	-3.70956600	2.16116900	1.81368000
C	-3.98874600	3.45467400	2.59802700
C	-4.43775400	0.96118100	2.43401600
C	-2.15421800	2.91154100	-2.95125600
C	-1.94347700	1.60124100	-3.72955100
C	-2.34323300	4.10775500	-3.89379200
C	-3.71001100	-2.16168900	-1.81368400
C	-3.98975400	-3.45505100	-2.59805600
C	-4.43825100	-0.96148200	-2.43355100
H	-1.25958900	7.28707600	0.30770300

H	1.19449300	7.27196200	0.69651800
H	2.43432700	5.12158000	0.68829000
H	-2.46272600	5.15199900	-0.08509200
H	2.47344300	0.00017900	-0.00040300
H	-2.46916700	-0.00009900	-0.00039200
H	2.43491200	-5.12118400	-0.68939400
H	1.19531100	-7.27169400	-0.69782600
H	-1.25876300	-7.28711800	-0.30898200
H	-2.46213000	-5.15221300	0.08406800
H	6.09543900	2.23021400	-0.82175600
H	5.07399800	2.21682500	3.33255400
H	5.07560900	-2.21578000	-3.33191300
H	6.09516900	-2.23021200	0.82287200
H	-4.85420700	-2.87689600	3.41609300
H	-6.15350000	-2.16948300	-0.59867600
H	-4.85569700	2.87611500	-3.41571000
H	-6.15350000	2.16833700	0.59948400
H	-7.09113400	2.62958300	-3.03133900
H	-7.90072300	1.07599900	-0.51371500
H	-7.28689900	0.35688600	-2.00796500
H	-8.84464000	1.21279300	-2.00450600
H	-7.93303900	3.69130800	-0.28020400
H	-8.90592100	3.76209700	-1.76008700
H	-7.38840000	4.67975500	-1.64367900
H	-7.08988000	-2.63169300	3.03236600
H	-7.93294000	-3.69121600	0.28072800
H	-8.90516400	-3.76306800	1.76097100
H	-7.38778500	-4.68078000	1.64318500
H	-7.89971300	-1.07598500	0.51613900
H	-7.28583400	-0.35822800	2.01101000
H	-8.84364400	-1.21400300	2.00683400
H	-1.22941500	-3.10025000	2.38563600
H	-2.83151900	-1.34930600	4.31598100
H	-1.09111800	-1.68724300	4.41521300
H	-1.74586400	-0.76977000	3.04216700
H	-3.22584400	-3.98341300	4.52989700
H	-1.47341500	-4.21665100	4.55405600
H	-2.46132900	-5.03636000	3.32722200
H	2.51457300	-2.18151400	1.90107600
H	4.65258000	-4.33819200	2.29305100
H	2.99071000	-4.61185300	1.74073200
H	3.29105300	-4.02824900	3.38949100
H	5.40465500	-1.91173400	2.87644400
H	3.92664100	-1.57204700	3.77898900
H	4.37830300	-0.54973900	2.39981700

H	7.78468200	-2.13375100	-0.69130900
H	7.57274300	-4.05661400	-2.27806100
H	7.24614900	-2.93128500	-3.60486600
H	8.81944100	-2.90287400	-2.79850500
H	6.93415900	-0.37588600	-3.05300900
H	8.54073400	-0.39763700	-2.29989600
H	7.12520000	0.16369300	-1.38031400
H	1.40729400	-2.72277400	-2.60237800
H	3.14131200	-0.85693300	-4.29498600
H	1.96394400	-0.35724300	-3.07076900
H	1.41691900	-1.19405100	-4.53868200
H	3.59268600	-3.48287500	-4.60320800
H	1.84851100	-3.69227200	-4.82293500
H	2.70108200	-4.60015200	-3.55720200
H	1.40601500	2.72350600	2.60134900
H	3.13929700	0.85771300	4.29474700
H	1.96245800	0.35798600	3.07003300
H	1.41479400	1.19483800	4.53767900
H	2.69938800	4.60091600	3.55669000
H	3.59046700	3.48366400	4.60317100
H	1.84618700	3.69306600	4.82201800
H	7.78430400	2.13486900	0.69327000
H	8.81802400	2.90292900	2.80123500
H	7.24448300	2.93056100	3.60709400
H	7.57130600	4.05679700	2.28110500
H	6.93251600	0.37556000	3.05342300
H	8.53960200	0.39786900	2.30138500
H	7.12470500	-0.16299400	1.38053100
H	2.51550200	2.18062000	-1.90165600
H	3.29147200	4.02781600	-3.38977700
H	2.99003700	4.61130600	-1.74118400
H	4.65231900	4.33864700	-2.29274200
H	4.38046900	0.55005300	-2.39966300
H	3.92881100	1.57216600	-3.77897500
H	5.40620100	1.91273000	-2.87575300
H	-2.63153200	1.95902400	1.89442700
H	-5.05035400	3.72185500	2.53561400
H	-3.40639300	4.29095900	2.19694800
H	-3.73019000	3.33323000	3.65670000
H	-5.52006800	1.12927700	2.47365200
H	-4.09487300	0.79402000	3.46100100
H	-4.26295300	0.04537600	1.86356100
H	-1.23048200	3.09947200	-2.38648700
H	-2.83406400	1.35040400	-4.31730100
H	-1.74793400	0.76970000	-3.04443000

H	-1.09363300	1.68796100	-4.41717200
H	-3.22791700	3.98506500	-4.52880800
H	-1.47537800	4.21752100	-4.55411400
H	-2.46200000	5.03672400	-3.32590000
H	-2.63197400	-1.95973100	-1.89486900
H	-5.05138000	-3.72206100	-2.53523000
H	-3.73162400	-3.33353500	-3.65682500
H	-3.40737800	-4.29148300	-2.19731500
H	-4.09583800	-0.79426300	-3.46068300
H	-4.26299100	-0.04577700	-1.86308000
H	-5.52062200	-1.12934200	-2.47266600

Table S5. B_6-hept [bent-zigzag(S_0)] in vacuum

Energies [B3LYP-D3(BJ)/6-31G(d)]

Electronic Energy (EE)	-4584.659933	Hartree
Zero-point Energy Correction	2.384566	Hartree
Thermal Correction to Energy	2.515424	Hartree
Thermal Correction to Enthalpy	2.516368	Hartree
Thermal Correction to Free Energy	2.202761	Hartree

Single-point energy [ωB97XD/6-311G(d,p)//B3LYP-D3(BJ)/6-31G(d)]

E(RωB97XD)	-4583.701688	Hartree

Cartesian coordinates [B3LYP-D3(BJ)/6-31G(d)]

C	6.41949000	-0.75010400	0.22282000
C	4.19991100	2.12697600	-3.60440600
H	4.32144100	1.30146500	-4.31564700
H	3.26813100	1.97022900	-3.05141100
H	4.09768300	3.05526300	-4.17803900
C	7.65140000	1.34933400	0.31981200
H	7.65769000	2.43612900	0.30938100
C	8.85856500	0.64876200	0.39038700
H	9.79951700	1.18885200	0.45504400
C	8.85403800	-0.74621700	0.36116400
H	9.79146600	-1.29460000	0.40337700
C	7.64248600	-1.43564500	0.26047300
H	7.64226800	-2.52106300	0.20413600
C	3.73522700	-0.74245600	0.29046100
C	3.73979400	0.67675800	0.32152100
C	2.51863400	1.35282700	0.45716600
H	2.51838400	2.43803200	0.48147000
C	1.29605000	0.67727400	0.55562000
C	1.29152500	-0.74285100	0.52661700
C	2.51117800	-1.41882900	0.39825700

H	2.50746900	-2.50403200	0.37681600
C	-1.38747300	-0.73200300	0.47713200
C	-1.38066100	0.68804300	0.49837100
C	-2.59082200	1.37466800	0.33772600
H	-2.58255400	2.46080400	0.35611800
C	-3.80998700	0.70804300	0.13982200
C	-3.81923000	-0.71121000	0.12451000
C	-2.60717800	-1.39860600	0.30195100
H	-2.61242900	-2.48486700	0.29240500
C	-6.48782000	-0.69058600	-0.22750400
C	-6.47748500	0.73371000	-0.21159700
C	-7.69295400	1.42370100	-0.33193200
H	-7.68352200	2.51023900	-0.35124100
C	-8.90646400	0.73902400	-0.43886100
H	-9.83881000	1.29154500	-0.52066000
C	-8.91633200	-0.65597100	-0.45743400
H	-9.85627100	-1.19290500	-0.55455500
C	-7.71280500	-1.36000600	-0.36665900
H	-7.71825500	-2.44577300	-0.41405300
C	5.04230600	-3.01461100	-0.40271600
C	5.20790200	-3.25908700	-1.78227500
C	5.20049600	-4.56931800	-2.26151400
H	5.32455000	-4.74725600	-3.32698300
C	5.02593800	-5.65878400	-1.40112500
C	4.83895900	-5.40251400	-0.04198700
H	4.69049200	-6.24333600	0.63129300
C	4.83637200	-4.09968800	0.46885900
C	5.34216100	-2.08834200	-2.74952000
H	5.31193800	-1.16134600	-2.16066100
C	4.15253300	-2.02681900	-3.72047500
H	4.23265500	-1.15316200	-4.37786300
H	4.10950500	-2.92177300	-4.35172500
H	3.20802200	-1.95861500	-3.17080400
C	6.68836300	-2.09571600	-3.48694500
H	7.51928800	-2.06864700	-2.77432400
H	6.79912500	-2.99577100	-4.10276400
H	6.77378600	-1.22382800	-4.14653100
C	5.03792100	-7.08373800	-1.92788900
H	4.88271200	-7.74965300	-1.06837000
C	3.89297300	-7.33471400	-2.92294000
H	2.92114800	-7.12747500	-2.46397300
H	3.98801600	-6.69047400	-3.80462800
H	3.89774900	-8.37589900	-3.26654800
C	6.39787800	-7.43870100	-2.55251800
H	6.60351400	-6.81239900	-3.42839500

H	7.21158800	-7.28634300	-1.83552400
H	6.41576300	-8.48553500	-2.87791900
C	4.65016800	-3.85846300	1.96127500
H	4.46262300	-2.78414500	2.09977500
C	3.43609800	-4.60652600	2.53250300
H	3.27282900	-4.33110800	3.58016800
H	2.52245100	-4.37954900	1.97637100
H	3.58498300	-5.69179800	2.49983700
C	5.93385400	-4.19936200	2.73725400
H	5.80914400	-3.99615900	3.80750500
H	6.18692800	-5.25970600	2.62011800
H	6.78141500	-3.60860000	2.37328500
C	5.04990500	2.97653800	-0.25034800
C	5.21747300	3.30366600	-1.61041400
C	5.16521600	4.64141700	-2.01405400
H	5.28439100	4.89123800	-3.06627400
C	4.94573500	5.67191800	-1.09821500
C	4.77106500	5.33386000	0.24741500
H	4.59443800	6.12406800	0.97153000
C	4.81235200	4.00747300	0.68169200
C	5.39710100	2.19680300	-2.64329200
H	5.42416000	1.23888900	-2.10655700
C	6.42408100	0.67367200	0.25384200
C	6.73089300	2.31366500	-3.39407100
H	7.57206200	2.29203700	-2.69329800
H	6.85160100	1.48302300	-4.09968900
H	6.78768300	3.24884400	-3.96297800
C	4.64702100	3.67822500	2.15967100
H	4.44344500	2.60076200	2.23671700
C	3.45901600	4.40927900	2.80163800
H	3.30686300	4.06358400	3.82977700
H	3.62999500	5.49115800	2.84360200
H	2.53307900	4.23984100	2.24569200
C	5.95213400	3.95026000	2.92770700
H	5.84489800	3.68625900	3.98655200
H	6.78175400	3.36807400	2.51251500
H	6.22123700	5.01145200	2.86696300
C	4.90267600	7.12208700	-1.54929300
H	5.01851700	7.12819700	-2.64146300
C	6.07097100	7.92497500	-0.95170800
H	7.03303800	7.46894500	-1.20841300
H	6.06692400	8.95611400	-1.32437500
H	6.00082500	7.96304600	0.14164000
C	3.55531200	7.78626700	-1.21973400
H	2.72609700	7.24389200	-1.68496000

H	3.37629000	7.80394400	-0.13876300
H	3.53465800	8.82171700	-1.57951100
C	-0.08109300	-3.02838100	1.08259700
C	-0.00514900	-4.07847000	0.15327800
C	-0.08633900	-5.40060900	0.60407900
H	-0.03193500	-6.21695300	-0.11244300
C	-0.24138100	-5.70586600	1.95689000
C	-0.29568900	-4.64915100	2.87459900
H	-0.40363200	-4.86863000	3.93408900
C	-0.21451200	-3.32095800	2.45457900
C	0.14720700	-3.77739400	-1.33105500
H	0.20366100	-2.68391300	-1.44120400
C	-1.07664100	-4.25401600	-2.12865300
H	-1.00249300	-3.94239300	-3.17699300
H	-1.15285700	-5.34708200	-2.11094900
H	-2.00684300	-3.85427600	-1.71678900
C	1.44775900	-4.36314100	-1.90286100
H	1.56369400	-4.09493300	-2.95924000
H	2.32927700	-4.00665100	-1.36354200
H	1.44254800	-5.45675400	-1.83697400
C	-0.35106000	-7.14989700	2.41420000
H	-0.26755600	-7.78061000	1.51902700
C	-1.72099600	-7.43230300	3.05296700
H	-1.81255600	-8.48939300	3.32912400
H	-1.86480000	-6.83372700	3.95991400
H	-2.53163700	-7.18322900	2.36069400
C	0.79495200	-7.53411200	3.36424100
H	0.72958000	-8.59159800	3.64628400
H	1.76834500	-7.36113000	2.89354900
H	0.76064700	-6.93970500	4.28454900
C	-0.24681200	-2.17781500	3.46324000
H	-0.14869500	-1.23737100	2.90184300
C	-1.58330000	-2.10036100	4.21374600
H	-1.58592500	-1.25619700	4.91325400
H	-2.41317300	-1.96748000	3.51255900
H	-1.76999600	-3.01525700	4.78713900
C	0.95077500	-2.23491300	4.42329400
H	1.89341400	-2.21324700	3.86703200
H	0.93722300	-1.38137900	5.11145000
H	0.93467900	-3.15236800	5.02282100
C	-0.05917200	2.95642000	1.16272000
C	0.03737800	4.01551400	0.24271900
C	-0.01649000	5.33257600	0.70332900
H	0.05281900	6.14850700	-0.01147700
C	-0.16359600	5.62523600	2.06297200

C	-0.24548200	4.56245000	2.96721700
H	-0.35263700	4.78250000	4.02736600
C	-0.19578700	3.23414300	2.53456200
C	0.17229400	3.72011300	-1.24451200
H	0.21840000	2.62684000	-1.36087500
C	-1.05606500	4.21332000	-2.02457700
H	-1.98637100	3.81725600	-1.60779900
H	-0.99548200	3.91217200	-3.07689400
H	-1.12217800	5.30547400	-1.99300000
C	1.47074200	4.29657200	-1.82846200
H	1.47099100	5.39039300	-1.76859100
H	1.57771900	4.02115100	-2.88386600
H	2.35351000	3.93924700	-1.29216800
C	-0.24357000	7.06367200	2.54251300
H	-0.34344000	7.03898300	3.63595100
C	-1.48745200	7.77088300	1.97650300
H	-2.39937200	7.21803600	2.22548600
H	-1.57632200	8.78703700	2.37863900
H	-1.43322000	7.84684000	0.88430700
C	1.03873700	7.84400500	2.20755200
H	0.98935600	8.86524300	2.60336400
H	1.91926600	7.35040800	2.63270100
H	1.18649900	7.91178900	1.12366400
C	-0.26898500	2.07986400	3.52765400
H	-0.23368100	1.14461500	2.95007500
C	-1.59342300	2.06113200	4.30304300
H	-1.62763500	1.20694200	4.98933900
H	-1.72115300	2.97453100	4.89478900
H	-2.44202800	1.98340200	3.61618800
C	0.94731700	2.05835500	4.46527800
H	1.87658400	1.98643500	3.89127100
H	0.99554300	2.97028500	5.07147000
H	0.89642700	1.20094100	5.14696900
C	-5.05426100	-3.01161300	-0.50983300
C	-4.97925000	-3.37634200	-1.86786800
C	-4.75378600	-4.71318600	-2.21362000
H	-4.66854400	-4.99114000	-3.26202500
C	-4.60384300	-5.70297100	-1.24122400
C	-4.70085800	-5.33076500	0.10391800
H	-4.59147300	-6.09174400	0.87025200
C	-4.91265300	-4.00445900	0.48249100
C	-5.07113600	-2.30447900	-2.94922200
H	-5.34746900	-1.36089600	-2.45990800
C	-6.17573300	-2.60249800	-3.97290900
H	-7.14546100	-2.71517300	-3.47646500

H	-6.25592900	-1.78747300	-4.70177600
H	-5.97092800	-3.52600700	-4.52643100
C	-3.71258200	-2.06845000	-3.62851000
H	-2.95357500	-1.78484400	-2.89238600
H	-3.36389600	-2.97340200	-4.13881100
H	-3.78625400	-1.26531300	-4.37173100
C	-4.34824200	-7.14774200	-1.63503500
H	-4.15751500	-7.16062300	-2.71665300
C	-5.58695000	-8.02047200	-1.36806000
H	-6.46301100	-7.63235600	-1.89848700
H	-5.41611600	-9.05385800	-1.69273900
H	-5.82524700	-8.03799300	-0.29797500
C	-3.10801200	-7.72963200	-0.93647200
H	-2.23039200	-7.09577100	-1.09579600
H	-3.26191600	-7.81231700	0.14528300
H	-2.88658800	-8.73390700	-1.31612700
C	-5.03033300	-3.61936900	1.95203900
H	-4.75020200	-2.55853300	2.03353400
C	-6.48778800	-3.74553900	2.43170600
H	-6.57923800	-3.44705900	3.48291600
H	-7.15765300	-3.11489300	1.83918200
H	-6.83051000	-4.78317100	2.34064700
C	-4.08610200	-4.40843800	2.86808700
H	-4.11010300	-3.99559000	3.88242700
H	-4.38364400	-5.46063500	2.94043800
H	-3.05356800	-4.37398800	2.50941800
C	-5.02632700	3.03378200	-0.48560900
C	-4.98624900	3.39724300	-1.84735200
C	-4.83690500	4.73980500	-2.20507700
H	-4.78968300	5.00665200	-3.25775500
C	-4.73598300	5.74341500	-1.23689300
C	-4.77239700	5.36909100	0.10826700
H	-4.68719400	6.14558600	0.86375200
C	-4.90447400	4.03385500	0.49903000
C	-5.06368100	2.31881100	-2.92371300
H	-5.22481500	1.35535700	-2.42130800
C	-6.26134400	2.52643600	-3.86176600
H	-7.19802600	2.54817500	-3.29474800
H	-6.17798700	3.47050700	-4.41233400
H	-6.32327000	1.71422100	-4.59565300
C	-3.74331400	2.19118600	-3.69910800
H	-2.91658900	1.95704300	-3.02123400
H	-3.81037200	1.39231600	-4.44737100
H	-3.49674900	3.12374400	-4.21946600
C	-4.54921400	7.20385600	-1.61403600

H	-4.71807000	7.79483400	-0.70371700
C	-5.56079100	7.67773600	-2.66839200
H	-5.45013800	8.75267200	-2.85209100
H	-5.41247400	7.16385800	-3.62500800
H	-6.58901000	7.48797200	-2.34262900
C	-3.10563900	7.47150600	-2.07385400
H	-2.95648700	8.53405300	-2.30059300
H	-2.39133000	7.17964500	-1.29686500
H	-2.87167100	6.89482200	-2.97630500
C	-4.96881800	3.65750200	1.97380800
H	-4.68208500	2.59852500	2.05287100
C	-6.40772700	3.78247400	2.50619300
H	-7.09659100	3.14738000	1.94029600
H	-6.46018300	3.48784200	3.56115700
H	-6.75739500	4.81848000	2.42378600
C	-3.99076300	4.45695100	2.84542400
H	-3.98384400	4.06331500	3.86753400
H	-2.97057600	4.40801300	2.45449800
H	-4.28013600	5.51254000	2.90710000
B	5.07066600	-1.51997600	0.07639700
B	5.08026200	1.45763600	0.14606000
B	-0.05300300	-1.51757600	0.66517500
B	-0.04255200	1.45571300	0.71445300
B	-5.14673800	-1.48339100	-0.16532900
B	-5.12320800	1.50459800	-0.14272200

Table S6. B_6-hept [twist1(S_0)] in vacuum

Energies [B3LYP-D3(BJ)/6-31G(d)]

Electronic Energy (EE)	-4584.660295	Hartree
Zero-point Energy Correction	2.384977	Hartree
Thermal Correction to Energy	2.515759	Hartree
Thermal Correction to Enthalpy	2.516703	Hartree
Thermal Correction to Free Energy	2.204298	Hartree

Single-point energy [ωB97XD/6-311G(d,p)/B3LYP-GD3BJ/6-31G(d)]

E(RωB97XD)	-4583.701859	Hartree

Cartesian coordinates [B3LYP-D3(BJ)/6-31G(d)]

C	-8.82732400	0.63265000	1.32968300
C	-8.85420700	-0.75371200	1.17373800
C	-7.68570500	-1.43717800	0.82693700
C	-7.63129100	1.32990900	1.13986800
C	-3.80518600	-0.77128800	0.29696000
C	-3.78033800	0.63867400	0.45868000

C	-2.59061900	-1.45368300	0.12845100
C	-2.54320700	1.29862600	0.44396500
C	1.31739200	-0.78021900	-0.21041700
C	1.30498600	0.64039800	-0.19183000
C	2.55025300	-1.43353300	-0.35548800
C	3.74561200	-0.73902400	-0.58666300
C	3.70112600	0.67434700	-0.72288600
C	2.48905600	1.33369300	-0.46903500
C	-6.46923000	-0.75794900	0.66493700
C	-6.44138800	0.65654800	0.82646400
B	-5.17633200	-1.51863400	0.23654300
B	-5.11481900	1.43845500	0.58655000
C	-1.33257700	0.61613800	0.27289400
B	0.02174400	1.38144700	0.27948300
C	-1.35179200	-0.79589300	0.11377600
B	-0.01157900	-1.58495300	-0.04761600
C	-5.10640800	2.99126400	0.35834000
C	0.13514500	2.80583400	0.92499500
C	-0.00564500	-3.15183500	-0.04690800
C	-5.23508600	-2.96093600	-0.37774400
C	-4.81579900	-4.10586900	0.33231400
C	-4.80690300	-5.34752500	-0.30461000
C	-5.20183800	-5.49033000	-1.63909600
C	-5.62409900	-4.35230200	-2.32822300
C	-5.64288000	-3.09265000	-1.71980200
C	-0.46638100	-3.87511400	-1.16734500
C	-0.48918000	-5.27158000	-1.12381600
C	-0.06279100	-5.97685400	0.00259400
C	0.40904800	-5.25145600	1.10138400
C	0.44757800	-3.85505200	1.09186500
C	-0.01997800	3.99442400	0.19069900
C	0.09738500	5.22536900	0.84069200
C	0.37215600	5.30505300	2.20966700
C	0.53078100	4.11518400	2.92540900
C	0.41352700	2.86938500	2.30322900
C	-5.41566000	3.46805500	-0.92965400
C	-5.38473100	4.84233800	-1.18546700
C	-5.04790400	5.76192100	-0.18988400
C	-4.73304700	5.27422900	1.08256400
C	-4.74998300	3.90728700	1.36728900
C	-5.03464400	7.25347000	-0.47909800
C	-3.66521900	7.88764900	-0.18458000
C	-6.15090700	7.97660400	0.29434000
C	0.49777000	6.65004100	2.90353700
C	1.67829900	7.46154100	2.34444400

C	-0.81153100	7.45270700	2.82010200
C	-0.31170400	3.93146500	-1.30219600
C	0.79814800	4.60337700	-2.12631100
C	-1.68989300	4.52348400	-1.63613500
C	0.90877700	-3.08279100	2.32352400
C	2.15107500	-3.68921700	2.98798600
C	-0.24167700	-2.91242000	3.33118600
C	-0.12622200	-7.49381700	0.02720100
C	1.24481500	-8.12232200	0.32242400
C	-1.18787100	-7.98720400	1.02492000
C	-0.83972800	-3.13196600	-2.44386400
C	-2.03116900	-3.74642200	-3.18764200
C	0.38511500	-3.00758700	-3.36892900
C	-6.04726400	-1.86070700	-2.52294300
C	-5.04699000	-1.56534500	-3.65142000
C	-7.48712100	-1.95916400	-3.04645700
C	-5.15171500	-6.84498100	-2.32460900
C	-6.07015600	-7.86661900	-1.63390300
C	-3.70936300	-7.37333400	-2.41534200
C	-4.43480400	-3.98600600	1.80214800
C	-5.63688300	-4.33185100	2.69929000
C	-3.20533300	-4.82169700	2.18103900
C	-4.41713900	3.40512000	2.76620700
C	-3.12950400	4.02638500	3.32551500
C	-5.60242300	3.61887700	3.72305600
C	-5.73490700	2.47983200	-2.04578500
C	-4.68024400	2.52813100	-3.16220100
C	-7.15854300	2.66347100	-2.59024400
C	0.59443700	1.57965200	3.09576900
C	-0.42146600	1.45146300	4.23980000
C	2.03995400	1.41553300	3.58791100
C	6.38531000	-0.69219900	-1.09054300
C	6.30808800	0.70153100	-1.37593200
C	7.60869400	-1.35605000	-1.26015600
C	8.73781400	-0.68002700	-1.73040100
C	8.66362000	0.68482000	-2.01082700
C	7.45953100	1.36775700	-1.82249700
B	4.97250000	1.47311100	-1.15051600
B	5.13458600	-1.44674100	-0.54214300
C	4.85273000	3.01958300	-1.39085200
C	5.27830900	-2.80399300	0.23409100
C	4.92284500	-4.05199400	-0.31657200
C	5.05216200	-5.20927300	0.45499300
C	5.51115300	-5.16340500	1.77516800
C	5.84139700	-3.91950600	2.31586400

C	5.73131500	-2.74378600	1.56828300
C	4.86713600	3.92392500	-0.30714600
C	4.71575700	5.29036000	-0.54678900
C	4.52983100	5.79072100	-1.84044700
C	4.49984800	4.88484100	-2.90132300
C	4.65889500	3.51022600	-2.69506400
C	4.37882000	7.28151300	-2.09009500
C	5.70329300	8.02008400	-1.82943200
C	3.23822800	7.89674300	-1.26400700
C	5.09345900	3.39744700	1.10488700
C	6.58653600	3.45523100	1.47395200
C	4.22946900	4.09991100	2.16072700
C	6.01373500	-1.39376000	2.21737900
C	4.89594100	-1.01973100	3.20488800
C	7.40154000	-1.32310900	2.86820900
C	5.64023600	-6.42985600	2.60366300
C	4.28897700	-7.14655800	2.76076800
C	6.70020600	-7.37747200	2.01698500
C	4.44680700	-4.14138900	-1.76076500
C	5.59080600	-4.59552900	-2.68417400
C	3.20990000	-5.03648700	-1.92673100
C	4.54128000	2.53682200	-3.86134000
C	3.08307000	2.43102400	-4.33934700
C	5.49316800	2.87769300	-5.01649000
H	-9.73702100	1.16953200	1.58548000
H	-9.78492400	-1.29865100	1.30821000
H	-7.71583600	-2.51227300	0.67061000
H	-7.61768700	2.41299400	1.22904700
H	-2.60915600	-2.53128700	-0.00571600
H	-2.52182400	2.37729200	0.56573400
H	2.57840400	-2.51514500	-0.26363200
H	2.47317300	2.41971000	-0.47020800
H	-4.48002300	-6.22479100	0.24580500
H	-5.92244900	-4.45098400	-3.36988000
H	-0.83963200	-5.83214900	-1.98531500
H	0.74582800	-5.78667300	1.98418300
H	-0.02707400	6.14179700	0.26955000
H	0.74750200	4.16881000	3.99020600
H	-5.61483600	5.21018200	-2.18328700
H	-4.46298600	5.97657600	1.86616600
H	-5.23639600	7.37989300	-1.55131500
H	-3.40859100	7.80288100	0.87720600
H	-3.66779500	8.95302700	-0.44323800
H	-2.87259500	7.39612800	-0.75775900
H	-5.99605800	7.88845200	1.37607400

H	-7.13137900	7.54757800	0.06185500
H	-6.17236900	9.04378500	0.04348800
H	0.70091400	6.45207800	3.96451300
H	2.61208700	6.89348800	2.41458900
H	1.80170500	8.40198500	2.89446700
H	1.51818000	7.70855300	1.28929400
H	-0.72497300	8.39879200	3.36740000
H	-1.64694600	6.88408600	3.24190300
H	-1.06192700	7.68908000	1.77971900
H	-0.33322900	2.86837400	-1.58802500
H	1.78462600	4.19400700	-1.89172200
H	0.83298700	5.68019000	-1.92528900
H	0.61921300	4.47097300	-3.19960500
H	-1.72012700	5.59215900	-1.39686500
H	-1.91337900	4.41365800	-2.70364000
H	-2.48955400	4.03971900	-1.06900100
H	1.19030600	-2.07571200	1.99131900
H	1.92969000	-4.66048800	3.44568400
H	2.51581100	-3.03062700	3.78368500
H	2.96082800	-3.83152100	2.26726400
H	-0.56454800	-3.88510900	3.71933700
H	-1.10828800	-2.43305400	2.86361300
H	0.07627300	-2.29426700	4.17992000
H	-0.43548300	-7.82154000	-0.97430700
H	1.59075400	-7.86195000	1.32857300
H	1.99869000	-7.77055300	-0.39020800
H	1.19304000	-9.21561400	0.26059100
H	-0.93815700	-7.67848600	2.04669700
H	-1.26175100	-9.08106500	1.00980100
H	-2.17169700	-7.57065700	0.78472100
H	-1.13118400	-2.11276600	-2.15817800
H	-2.89432400	-3.87323900	-2.52893500
H	-1.77934600	-4.72614100	-3.61007400
H	-2.32960300	-3.10282000	-4.02208800
H	0.72060100	-3.99847500	-3.69574000
H	1.22170100	-2.52163700	-2.85617100
H	0.13949900	-2.41874400	-4.26082900
H	-6.02052100	-0.99648500	-1.84699700
H	-5.33656000	-0.65887300	-4.19622800
H	-5.00165100	-2.39200200	-4.36944300
H	-4.04051300	-1.41619500	-3.24765600
H	-8.19068500	-2.10065800	-2.21935100
H	-7.60430200	-2.80109600	-3.73843500
H	-7.76683800	-1.04348600	-3.58091400
H	-5.51974300	-6.70586200	-3.35002500

H	-6.06576600	-8.82075500	-2.17385500
H	-5.73965500	-8.06278100	-0.60733800
H	-7.10148200	-7.50092500	-1.58833600
H	-3.67833400	-8.33159200	-2.94727900
H	-3.28289600	-7.52847500	-1.41779500
H	-3.06573200	-6.66313500	-2.94350400
H	-4.18162100	-2.93335200	1.99135500
H	-5.95339500	-5.36857200	2.53404700
H	-6.49041100	-3.68027800	2.48481300
H	-5.37826000	-4.21879900	3.75886900
H	-2.35789000	-4.61686300	1.52113300
H	-3.41754200	-5.89538300	2.13049700
H	-2.89910000	-4.60003600	3.20895500
H	-4.24752200	2.32085000	2.69442400
H	-2.28907400	3.89846700	2.63840800
H	-2.86211300	3.56180700	4.28099000
H	-3.25153800	5.10028600	3.50761300
H	-5.83601400	4.68623300	3.81485300
H	-5.37151100	3.23398000	4.72347500
H	-6.49983800	3.10765600	3.35896300
H	-5.69105100	1.46803500	-1.61870600
H	-4.65393500	3.51390800	-3.64043000
H	-4.89974200	1.78255400	-3.93536600
H	-3.68238200	2.32345100	-2.76098000
H	-7.28725400	3.65510600	-3.03931800
H	-7.89593200	2.55728500	-1.78759500
H	-7.37887900	1.91405700	-3.35999600
H	0.39901700	0.73932600	2.41520900
H	-0.29936800	2.25527400	4.97483200
H	-0.29542800	0.49545700	4.76167400
H	-1.44502500	1.50019200	3.85479100
H	2.31757700	2.22345700	4.27458100
H	2.73933900	1.43428100	2.74613500
H	2.16544300	0.46297800	4.11583600
H	7.67548900	-2.41281400	-1.01520300
H	9.67490700	-1.21352800	-1.86670100
H	9.54254600	1.21645100	-2.36586800
H	7.40646900	2.43476100	-2.02282700
H	4.78501000	-6.16892000	0.02176400
H	6.17607400	-3.87063000	3.34965600
H	4.73447100	5.98292300	0.28931000
H	4.33346300	5.26293300	-3.90761800
H	4.12883800	7.40984000	-3.15187000
H	6.51056100	7.60806000	-2.44425500
H	6.00146500	7.92392300	-0.77876300

H	5.60703000	9.08860100	-2.05614600
H	3.44896000	7.83684200	-0.19094800
H	2.29370500	7.37385100	-1.44564800
H	3.10320800	8.95468000	-1.51771800
H	4.80083800	2.33736900	1.10823600
H	6.94134200	4.49278100	1.47822700
H	7.19221300	2.89419500	0.75468200
H	6.75831700	3.03169900	2.47108200
H	4.53227200	5.14474700	2.29740400
H	4.33550700	3.60184500	3.13067000
H	3.17079100	4.08726400	1.88614900
H	6.00272700	-0.63240000	1.42748100
H	4.84864000	-1.73491000	4.03434900
H	3.92238600	-1.02060800	2.70448300
H	5.06384400	-0.02070900	3.62466800
H	7.58584700	-0.32030500	3.27077700
H	8.18460600	-1.54772600	2.13621200
H	7.49692000	-2.03594800	3.69514200
H	5.97792000	-6.13101500	3.60509600
H	3.53900100	-6.47920800	3.19755800
H	4.38832300	-8.02687000	3.40669900
H	3.90834100	-7.48470600	1.79093600
H	6.41055000	-7.71694400	1.01565400
H	6.82299200	-8.26473800	2.64929100
H	7.67088000	-6.87743800	1.93200800
H	4.15843600	-3.12837100	-2.07621200
H	5.93938800	-5.59607000	-2.40205000
H	6.44378600	-3.91145200	-2.62009300
H	5.25916800	-4.63223800	-3.72867000
H	3.44556400	-6.08691100	-1.72038700
H	2.83587700	-4.98211700	-2.95457100
H	2.40079200	-4.73879000	-1.25405000
H	4.83169100	1.54239100	-3.49475200
H	2.72237300	3.39768200	-4.70935200
H	2.42696800	2.12058600	-3.51944900
H	2.99048500	1.69936900	-5.15076800
H	5.25498800	3.85141900	-5.45935300
H	5.42076200	2.12553500	-5.81044000
H	6.53174800	2.91095300	-4.67007600

Table S7. B_6-hept [twist2(S_0)] in vacuum

Energies [B3LYP-D3(BJ)/6-31G(d)]

Electronic Energy (EE)	-4584.657348	Hartree
Zero-point Energy Correction	2.384893	Hartree

Thermal Correction to Energy	2.515583	Hartree
Thermal Correction to Enthalpy	2.516528	Hartree
Thermal Correction to Free Energy	2.204051	Hartree

Single-point energy [ωB97XD/6-311G(d,p)//B3LYP-D3(BJ)/6-31G(d)]

E(RωB97XD)	-4583.698575	Hartree

Cartesian coordinates [B3LYP-D3(BJ)/6-31G(d)]

C	8.91663400	0.70911700	-0.11984200
C	8.92537000	-0.68269700	-0.21625100
C	7.71734300	-1.38412400	-0.24655900
C	7.70011700	1.39387000	-0.05610700
C	3.79745200	-0.71364100	-0.23127500
C	3.79513200	0.70549300	-0.28048000
C	2.56645500	-1.38583000	-0.20175500
C	2.57172400	1.37732500	-0.42633900
C	-1.34423500	-0.70648500	-0.28383800
C	-1.34310200	0.70350700	-0.46139700
C	-2.56954600	-1.37996200	-0.20210300
C	-3.79898000	-0.70492600	-0.23152900
C	-3.79339300	0.71420100	-0.28062000
C	-2.56844300	1.38321100	-0.42657700
C	6.48558500	-0.71388500	-0.19994300
C	6.47778700	0.70815000	-0.10801200
B	5.14312200	-1.50710800	-0.22194700
B	5.12618600	1.48323400	-0.04266100
C	1.34482400	0.70045900	-0.46119100
B	0.00174300	1.46740300	-0.66110600
C	1.34269700	-0.70953100	-0.28360600
B	-0.00163500	-1.45356800	-0.04529500
C	5.05043100	2.98634600	0.40343900
C	0.00337900	2.95873900	-1.14628000
C	-0.00321800	-2.84574300	0.67229600
C	5.09595700	-3.07560200	-0.30098800
C	5.20501300	-3.70062300	-1.55877300
C	5.08765700	-5.09034400	-1.65927100
C	4.87520200	-5.88559900	-0.53021300
C	4.76869400	-5.25396900	0.71140000
C	4.86452100	-3.86684000	0.84366000
C	-0.00259900	-2.84782600	2.07932800
C	-0.00372400	-4.06773600	2.76177500
C	-0.00549500	-5.28670400	2.07619400
C	-0.00621500	-5.26551500	0.67756900
C	-0.00511100	-4.06164700	-0.03015900
C	0.00533300	4.03003400	-0.23460100

C	0.00667800	5.34210500	-0.71260300
C	0.00607000	5.61918100	-2.08374400
C	0.00406100	4.54532500	-2.97755200
C	0.00274200	3.22175400	-2.52719000
C	5.07132000	3.27576400	1.78279900
C	4.94024200	4.59972900	2.21205500
C	4.79169600	5.65161200	1.30527400
C	4.76684800	5.35025900	-0.05973800
C	4.88149300	4.03675800	-0.52020100
C	4.68051600	7.08590600	1.79380900
C	3.43341400	7.79629600	1.24389900
C	5.95496200	7.87986400	1.45687700
C	0.00745600	7.05066300	-2.59071700
C	-1.25856500	7.80493700	-2.15090800
C	1.27573900	7.80201600	-2.15244400
C	0.00562200	3.74967500	1.26167900
C	-1.26351300	4.28862400	1.93828000
C	1.27565100	4.28711000	1.93776800
C	-0.00575800	-4.04828800	-1.55187500
C	-1.27330700	-4.69949500	-2.12498400
C	1.26046000	-4.70126300	-2.12593400
C	-0.00657200	-6.60160200	2.83517800
C	-1.27727800	-7.41791700	2.54287300
C	1.26226200	-7.42048700	2.54187700
C	-0.00096300	-1.52205000	2.83426600
C	1.27181600	-1.33475300	3.67271100
C	-1.27318100	-1.33194300	3.67294200
C	4.77167700	-3.20430800	2.21267300
C	6.16440300	-3.09828500	2.86093900
C	3.78065700	-3.89378000	3.15983100
C	4.71825900	-7.39382700	-0.63126200
C	5.84456900	-8.05317300	-1.44137200
C	3.33778100	-7.76414200	-1.20036800
C	5.39577200	-2.85324600	-2.81194100
C	6.63108700	-3.27726400	-3.61958900
C	4.12665300	-2.84250300	-3.67951900
C	4.86203600	3.73638700	-2.01356100
C	3.72859600	4.46267100	-2.75210000
C	6.22833700	4.04083600	-2.65167700
C	5.14909400	2.13459200	2.79096700
C	3.76231400	1.83022800	3.38502700
C	6.19326800	2.37341600	3.89001600
C	0.00019000	2.06077600	-3.51372600
C	1.27163700	2.04153000	-4.37532900
C	-1.27604600	2.04180500	-4.36826700

C	-6.48709300	-0.69896300	-0.19924400
C	-6.47596300	0.72302400	-0.10686300
C	-7.72041400	-1.36634900	-0.24537300
C	-8.92680700	-0.66215300	-0.21411500
C	-8.91481300	0.72959900	-0.11719000
C	-7.69668300	1.41153000	-0.05395000
B	-5.12254200	1.49495900	-0.04199800
B	-5.14647100	-1.49527800	-0.22198700
C	-5.04293300	2.99772800	0.40460600
C	-5.10311400	-3.06386700	-0.30131800
C	-4.87335900	-3.85584800	0.84315900
C	-4.78147800	-5.24322400	0.71073100
C	-4.89031500	-5.87445200	-0.53089300
C	-5.10093300	-5.07849800	-1.65979800
C	-5.21436000	-3.68846000	-1.55912000
C	-4.87207500	4.04818800	-0.51864100
C	-4.75387900	5.36116600	-0.05759800
C	-4.77699800	5.66189800	1.30758800
C	-4.92745300	4.60993700	2.21394800
C	-5.06211800	3.28651200	1.78412100
C	-4.66203100	7.09568100	1.79674900
C	-5.93481400	7.89285700	1.46113300
C	-3.41363600	7.80336600	1.24628300
C	-4.85443200	3.74844600	-2.01215200
C	-6.22105000	4.05465500	-2.64877100
C	-3.72103200	4.47382900	-2.75161000
C	-5.40304900	-2.84039500	-2.81213000
C	-4.13385000	-2.83266600	-3.67963200
C	-6.63938000	-3.26115500	-3.61991700
C	-4.73752100	-7.38310700	-0.63201100
C	-5.86429900	-8.03930800	-1.44398400
C	-3.35714700	-7.75710100	-1.19901100
C	-4.77812600	-3.19374400	2.21221700
C	-6.17015400	-3.08486200	2.86149100
C	-3.78783500	-3.88550000	3.15848500
C	-5.14210600	2.14507700	2.79182700
C	-3.75584500	1.83732700	3.38534300
C	-6.18540800	2.38581600	3.89128700
H	9.85383100	1.25856800	-0.08715400
H	9.86914900	-1.21986700	-0.26028300
H	7.72431400	-2.46936300	-0.30681400
H	7.69455700	2.47654700	0.03867800
H	2.56101600	-2.46607000	-0.08799500
H	2.57125100	2.46131400	-0.49269600
H	-2.56660900	-2.46021600	-0.08840100

H	-2.56545900	2.46720700	-0.49288900
H	5.15280100	-5.55950400	-2.63747400
H	4.59682400	-5.86878300	1.59044000
H	-0.00326800	-4.07829000	3.84981300
H	-0.00762500	-6.20529900	0.13090800
H	0.00817000	6.16636600	-0.00402400
H	0.00350200	4.75237700	-4.04563100
H	4.93951800	4.82374800	3.27627700
H	4.64808800	6.15650600	-0.77779600
H	4.58900400	7.05093400	2.88776300
H	3.48140700	7.89793900	0.15429100
H	3.34381100	8.80338900	1.66755000
H	2.52295600	7.23812700	1.48469100
H	6.09548500	7.94556100	0.37149800
H	6.84215900	7.39631200	1.87934500
H	5.89655800	8.90105800	1.85206300
H	0.00675000	7.00804600	-3.68813200
H	-1.30329300	7.89324100	-1.05980700
H	-2.16154600	7.27915300	-2.47873000
H	-1.27526200	8.81776000	-2.57049800
H	1.32204200	7.89018100	-1.06139700
H	1.29422400	8.81481500	-2.57201600
H	2.17711400	7.27419900	-2.48142100
H	0.00501600	2.65640800	1.39239200
H	-2.17002500	3.90097500	1.46652400
H	-1.30235400	5.38186200	1.87653900
H	-1.28541400	4.01347900	2.99902500
H	1.31573600	5.38031300	1.87617000
H	1.29767600	4.01183600	2.99847300
H	2.18150800	3.89844300	1.46559800
H	-0.00515200	-2.99343700	-1.86872000
H	-1.29458100	-5.76995700	-1.89678300
H	-1.30816700	-4.58968900	-3.21524800
H	-2.18176100	-4.25709700	-1.70655900
H	1.28029300	-5.77178700	-1.89790100
H	2.16981900	-4.26028300	-1.70798500
H	1.29480300	-4.59132700	-3.21620400
H	-0.00591000	-6.35943500	3.90639500
H	-1.32055700	-7.71500900	1.48870400
H	-2.17720100	-6.83233200	2.75967100
H	-1.30313600	-8.33144100	3.14866100
H	1.30411600	-7.71761200	1.48766100
H	1.28674300	-8.33408200	3.14761600
H	2.16353000	-6.83672400	2.75799400
H	-0.00014200	-0.71578500	2.08863500

H	1.35773000	-2.10796200	4.44438700
H	1.26565800	-0.35797200	4.17038800
H	2.16314300	-1.38953300	3.03984900
H	-1.36069500	-2.10503700	4.44455600
H	-2.16474100	-1.38466500	3.04022800
H	-1.26474000	-0.35522100	4.17069800
H	4.40682000	-2.17862600	2.05333100
H	6.58327300	-4.09753200	3.02962900
H	6.10495100	-2.58557000	3.82862900
H	6.85937700	-2.54406700	2.22270900
H	2.79684000	-4.01197000	2.69614500
H	4.13828900	-4.88537900	3.46091700
H	3.65856200	-3.30328900	4.07402000
H	4.76855800	-7.79151000	0.39134700
H	5.82215600	-7.73492600	-2.48993600
H	5.74192900	-9.14440000	-1.42637000
H	6.82768000	-7.79405500	-1.03424800
H	3.22488700	-7.38698300	-2.22345700
H	2.53893600	-7.32573300	-0.59337300
H	3.20108900	-8.85205600	-1.22181300
H	5.57044100	-1.81725900	-2.48965500
H	6.53087700	-4.30156600	-3.99623100
H	7.53469500	-3.23356400	-3.00216700
H	6.77357400	-2.61729200	-4.48306700
H	3.87783300	-3.85257600	-4.02454400
H	4.26439600	-2.20607100	-4.56172500
H	3.27099200	-2.46273000	-3.11198300
H	4.68244300	2.65715000	-2.12869900
H	3.89163400	5.54651300	-2.76562900
H	2.75765900	4.27750900	-2.28461000
H	3.67423600	4.12820100	-3.79394300
H	6.47511200	5.10364800	-2.54317900
H	6.22287200	3.80012000	-3.72144900
H	7.02425000	3.45954200	-2.17444000
H	5.46771400	1.23465700	2.24776700
H	3.38195700	2.69155000	3.94615000
H	3.81105600	0.97238000	4.06642400
H	3.04038100	1.59853600	2.59533200
H	5.92774100	3.22955400	4.52047200
H	7.18005000	2.56714000	3.45618700
H	6.27074500	1.49563600	4.54173700
H	0.00175400	1.12869000	-2.92962400
H	1.35370100	2.95161100	-4.98057700
H	1.26693400	1.18231700	-5.05618700
H	2.16425800	1.97633600	-3.74466200

H	-1.36124500	2.95206300	-4.97282000
H	-2.16515100	1.97666300	-3.73263400
H	-1.27529700	1.18268700	-5.04926900
H	-7.72991400	-2.45154900	-0.30597600
H	-9.87184600	-1.19713300	-0.25779100
H	-9.85072300	1.28119200	-0.08371900
H	-7.68858300	2.49415100	0.04126400
H	-4.61101400	-5.85859900	1.58965700
H	-5.16777300	-5.54736700	-2.63801700
H	-4.63365400	6.16748600	-0.77532200
H	-4.92542300	4.83341800	3.27828300
H	-4.56982600	7.05995000	2.89062000
H	-6.07597300	7.95941800	0.37589000
H	-5.87366500	8.91371700	1.85676700
H	-6.82285800	7.41123200	1.88402200
H	-3.46218200	7.90565000	0.15676200
H	-2.50433100	7.24291600	1.48613200
H	-3.32132300	8.81003700	1.67035700
H	-4.67611200	2.66905700	-2.12791200
H	-6.46655300	5.11768000	-2.53946400
H	-7.01712500	3.47396300	-2.17109000
H	-6.21687200	3.81449600	-3.71867500
H	-3.88319400	5.55780900	-2.76492700
H	-3.66789800	4.13938100	-3.79351500
H	-2.74984300	4.28777100	-2.28499500
H	-5.57513900	-1.80403300	-2.48965200
H	-3.88759900	-3.84329300	-4.02488300
H	-3.27725900	-2.45522900	-3.11193900
H	-4.26990500	-2.19566500	-4.56169000
H	-6.54170000	-4.28559800	-3.99683800
H	-6.78020900	-2.60060600	-4.48322400
H	-7.54289300	-3.21537900	-3.00250500
H	-4.79051000	-7.78082000	0.39045200
H	-5.83917000	-7.72133100	-2.49256700
H	-6.84738100	-7.77735700	-1.03860500
H	-5.76477400	-9.13081600	-1.42865000
H	-3.24168300	-7.38008200	-2.22186600
H	-3.22337500	-8.84537900	-1.22040600
H	-2.55805000	-7.32094100	-0.59072500
H	-4.41120000	-2.16881700	2.05281100
H	-6.59102800	-4.08323400	3.03037300
H	-6.86439900	-2.52910900	2.22379100
H	-6.10894000	-2.57237400	3.82919100
H	-4.14724200	-4.87647300	3.45951800
H	-3.66390100	-3.29553800	4.07276600

H	-2.80458200	-4.00553400	2.69408000
H	-5.46293100	1.24608800	2.24836400
H	-3.37332500	2.69758800	3.94662400
H	-3.03469600	1.60421900	2.59535100
H	-3.80635100	0.97937400	4.06647800
H	-5.91777800	3.24112800	4.52197600
H	-6.26464600	1.50796900	4.54270800
H	-7.17188800	2.58190500	3.45783300

Table S8. B_6-hept [helical(S_0)] in vacuum

Energies [B3LYP-D3(BJ)/6-31G(d)]

Electronic Energy (EE)	-4584.658226	Hartree
Zero-point Energy Correction	2.386098	Hartree
Thermal Correction to Energy	2.516350	Hartree
Thermal Correction to Enthalpy	2.517295	Hartree
Thermal Correction to Free Energy	2.208018	Hartree

Single-point energy [ωB97XD/6-311G(d,p)//B3LYP-D3(BJ)/6-31G(d)]

E(RωB97XD)	-4583.699816	Hartree

Cartesian coordinates [B3LYP-D3(BJ)/6-31G(d)]

C	8.24175800	-3.41979600	0.00954100
C	8.66476800	-2.17939300	0.48817700
C	7.72904000	-1.16783200	0.71927400
C	6.88519000	-3.64219700	-0.24141700
C	3.81372300	-0.51568600	0.46827100
C	3.37430200	-1.82837500	0.14763100
C	2.85786300	0.50711200	0.55389900
C	1.99753500	-2.08603200	0.10705300
C	-1.06688100	1.09281200	0.27894600
C	-1.50687700	-0.24810900	0.44568100
C	-2.02932300	2.09648200	0.10065400
C	-3.40637900	1.83605000	0.16034800
C	-3.84159300	0.52142200	0.47509100
C	-2.88053300	-0.49670200	0.57288700
C	6.36045300	-1.37724900	0.49093000
C	5.93013400	-2.64403500	-0.00039600
B	5.32464500	-0.23909200	0.74612200
B	4.42072800	-2.89366400	-0.30055000
C	1.04004600	-1.07926100	0.29207800
B	-0.48092500	-1.41723300	0.34693600
C	1.48342300	0.26396000	0.42511000
B	0.45819900	1.42809700	0.27041100
C	3.94733900	-4.08184700	-1.21164600

C	-0.98724800	-2.89555700	0.24279300
C	0.94761700	2.89437200	0.01485700
C	5.75472100	1.14016900	1.36107200
C	5.97296200	1.23662400	2.74883200
C	6.33365000	2.46574400	3.31019300
C	6.48224900	3.61123100	2.52678700
C	6.25084200	3.50642400	1.15111100
C	5.88336600	2.29548300	0.56151600
C	0.91617000	3.42797600	-1.29221900
C	1.32360100	4.74723700	-1.50357900
C	1.76638300	5.55604600	-0.45447500
C	1.81157700	5.01056700	0.83175100
C	1.41341800	3.69412300	1.07864400
C	-1.05834500	-3.51812400	-1.02219400
C	-1.56506000	-4.81623500	-1.12355400
C	-1.98821900	-5.52407300	0.00621800
C	-1.89179800	-4.90367000	1.25324900
C	-1.40812100	-3.60002000	1.38833300
C	4.08417100	-3.93664900	-2.60803100
C	3.63881200	-4.95471200	-3.45585100
C	3.05491300	-6.12044600	-2.95572000
C	2.91143400	-6.24598400	-1.57070100
C	3.33677200	-5.24295500	-0.69662600
C	2.58275900	-7.21810100	-3.89358500
C	1.07998900	-7.50067700	-3.73180200
C	3.40685700	-8.50348400	-3.70693900
C	-2.56911400	-6.92218500	-0.10895600
C	-3.96644400	-6.87944600	-0.75217600
C	-1.63601500	-7.88061000	-0.86478500
C	-0.63676900	-2.74288100	-2.26641800
C	-1.84627300	-2.08654600	-2.95523600
C	0.17573700	-3.58830600	-3.25490900
C	1.41725300	3.13646700	2.49699800
C	0.18462100	3.61750300	3.28149500
C	2.71641400	3.43917800	3.25546900
C	2.17925700	6.99417200	-0.71330100
C	1.22060500	7.98048000	-0.02497900
C	3.63526000	7.25393400	-0.29422500
C	0.52146800	2.53115800	-2.46028000
C	1.75098600	1.78081600	-3.00826800
C	-0.21343800	3.26732400	-3.58615200
C	5.67226600	2.20122900	-0.94413700
C	7.02161100	2.07031500	-1.67198300
C	4.83951700	3.36144900	-1.50666100
C	6.88380500	4.93542300	3.15331700

C	8.27168700	5.38430800	2.66483900
C	5.83316900	6.02943900	2.90198100
C	5.73159800	0.02504700	3.64047300
C	6.92287300	-0.28679000	4.55662800
C	4.42632200	0.18995700	4.43758700
C	3.17907900	-5.41835200	0.80824500
C	1.77928800	-5.91057300	1.20580200
C	4.27600700	-6.34009200	1.36871300
C	4.62373900	-2.63214100	-3.18361200
C	3.47290700	-1.74943700	-3.69888500
C	5.69960800	-2.84424400	-4.25689700
C	-1.25951800	-2.95704300	2.76135400
C	0.11590800	-3.28855300	3.36952500
C	-2.39002300	-3.31905200	3.73262400
C	-5.96695300	2.64993900	0.04919500
C	-6.39163000	1.37512200	0.52310700
C	-6.92506600	3.65193900	-0.16268100
C	-8.27694700	3.42893200	0.11142500
C	-8.69331900	2.18305200	0.58188800
C	-7.75604000	1.16512300	0.77466600
B	-5.35591300	0.22608600	0.72120300
B	-4.46105100	2.90624400	-0.26270500
C	-5.76934100	-1.20127000	1.22852400
C	-3.98038300	4.10662300	-1.15220300
C	-4.09691600	3.97785800	-2.55133800
C	-3.58735200	4.98155800	-3.38144700
C	-2.95922000	6.11408600	-2.85955900
C	-2.85027900	6.22938500	-1.46995400
C	-3.34340000	5.24355700	-0.61347500
C	-5.93286600	-1.41560300	2.61117400
C	-6.20151500	-2.70180700	3.08976500
C	-6.32809400	-3.78924000	2.22118200
C	-6.17510700	-3.56120700	0.85110300
C	-5.88727900	-2.29273700	0.34261200
C	-6.57701700	-5.19770100	2.73431500
C	-7.75786800	-5.27234400	3.71367600
C	-5.29901300	-5.78248200	3.36087600
C	-5.76015600	-0.25461400	3.58425100
C	-6.98761900	-0.06454900	4.48720300
C	-4.46904000	-0.39732000	4.40571600
C	-3.23967300	5.40525400	0.89773300
C	-4.40390500	6.25702700	1.43448400
C	-1.88839400	5.97152200	1.35468200
C	-2.39245400	7.18328100	-3.77812200
C	-3.05528900	8.54956900	-3.53670500

C	-0.86264600	7.28211800	-3.64851900
C	-4.69428500	2.70805000	-3.14859200
C	-5.74501700	2.99043800	-4.23097700
C	-3.59055500	1.76761300	-3.66418800
C	-5.74722000	-2.06323200	-1.15707800
C	-5.11617300	-3.24673800	-1.90158200
C	-7.10550000	-1.68774100	-1.77815900
H	8.96682300	-4.20800000	-0.17576600
H	9.71999200	-1.99915700	0.67597900
H	8.06051900	-0.19918400	1.08398200
H	6.55910800	-4.60144600	-0.63487100
H	3.19716900	1.52748900	0.70642500
H	1.65543800	-3.09590300	-0.09920600
H	-1.69275700	3.10523100	-0.12082200
H	-3.21438900	-1.51802800	0.73408100
H	6.48748600	2.54290300	4.38420600
H	6.35594800	4.39041500	0.52886500
H	1.30417900	5.16407700	-2.50575900
H	2.15549800	5.62639200	1.65790700
H	-1.63478800	-5.28574300	-2.10027200
H	-2.20059300	-5.45820600	2.13454700
H	3.72909600	-4.83679000	-4.53311800
H	2.45182300	-7.14316100	-1.16602900
H	2.74690700	-6.86284100	-4.91978900
H	0.85471400	-7.87843800	-2.72861500
H	0.74664600	-8.25421500	-4.45505700
H	0.49071800	-6.59069700	-3.88304500
H	3.27494300	-8.90899800	-2.69696800
H	4.47509700	-8.31033500	-3.85201400
H	3.09505800	-9.27439200	-4.42154900
H	-2.68723400	-7.30882100	0.91231000
H	-3.91203700	-6.49346200	-1.77678300
H	-4.63453500	-6.22237900	-0.18531300
H	-4.41251600	-7.88048600	-0.78960100
H	-1.51144800	-7.57476700	-1.90926300
H	-2.04156500	-8.89903500	-0.86336300
H	-0.64213400	-7.90336300	-0.40430000
H	0.02040400	-1.92837900	-1.93506000
H	-2.40965400	-1.45960700	-2.25621800
H	-2.52766900	-2.84851300	-3.34833900
H	-1.51917100	-1.45722000	-3.79180100
H	-0.43893800	-4.37315500	-3.71108600
H	0.55783700	-2.96089600	-4.06737600
H	1.02794600	-4.06708700	-2.76558800
H	1.34040100	2.04336700	2.41951300

H	0.19956400	4.70743600	3.39449700
H	0.16283400	3.17079900	4.28270700
H	-0.74173000	3.34686800	2.76365900
H	2.81738100	4.51089800	3.46221100
H	3.59647400	3.12304200	2.68909900
H	2.72653300	2.91850800	4.21923500
H	2.10748800	7.16300400	-1.79601600
H	1.24934500	7.86024200	1.06421100
H	0.18831500	7.81137400	-0.34871000
H	1.49167900	9.01706400	-0.25768800
H	3.76300400	7.12636700	0.78632600
H	3.93757300	8.27675700	-0.54799400
H	4.31671500	6.55731700	-0.79431300
H	-0.17071700	1.77286300	-2.07052000
H	2.46901000	2.48762400	-3.43958400
H	1.45522800	1.07303900	-3.79208000
H	2.26338500	1.22120500	-2.21978900
H	0.44886400	3.96837600	-4.10760700
H	-1.07269500	3.82633100	-3.20539500
H	-0.57483300	2.55001500	-4.33079800
H	5.10827500	1.27839800	-1.14065000
H	7.63943500	2.96002600	-1.50164800
H	6.87357500	1.95990700	-2.75294900
H	7.57879700	1.19899900	-1.31120800
H	3.88843700	3.46738300	-0.97760300
H	5.37497800	4.31487900	-1.42975300
H	4.62293300	3.19473800	-2.56733500
H	6.94734400	4.77680000	4.23824000
H	9.02761400	4.61921000	2.87138500
H	8.57752800	6.31425600	3.15864500
H	8.26580100	5.56451400	1.58348800
H	4.84854000	5.71932400	3.26622200
H	5.73853400	6.24696300	1.83253200
H	6.11296700	6.96048400	3.40876900
H	5.59843000	-0.84820100	2.98756800
H	7.11309500	0.53009700	5.26195500
H	7.83435900	-0.44462800	3.97015900
H	6.73131800	-1.19304200	5.14259400
H	4.48604600	1.05299600	5.11081000
H	4.22300100	-0.70128800	5.04308500
H	3.57849400	0.34912500	3.76288900
H	3.31753900	-4.43052800	1.27122400
H	1.59932600	-6.93344800	0.85510700
H	0.99307200	-5.27362000	0.79085400
H	1.67310200	-5.91613300	2.29601500

H	4.20327300	-7.33996400	0.92470500
H	4.18289500	-6.44294500	2.45640800
H	5.27306900	-5.94487300	1.14641100
H	5.10451500	-2.08090600	-2.36521700
H	2.95315900	-2.23538700	-4.53265800
H	3.85029400	-0.78116000	-4.04902400
H	2.73921000	-1.56403100	-2.90765700
H	5.29549800	-3.34931900	-5.14149000
H	6.52385400	-3.45172700	-3.86819500
H	6.10776700	-1.88142300	-4.58494300
H	-1.29567300	-1.86787600	2.61610400
H	0.20656500	-4.36698000	3.54246000
H	0.24993500	-2.77607500	4.32971800
H	0.92935700	-2.98579600	2.70235400
H	-2.34955100	-4.37563800	4.02051900
H	-3.37286700	-3.12628300	3.29265600
H	-2.30289300	-2.72831400	4.65096700
H	-6.60500200	4.61473100	-0.55222500
H	-9.00374900	4.22078900	-0.04978100
H	-9.74481400	2.00264000	0.78921100
H	-8.08299700	0.18911100	1.12389900
H	-3.66048300	4.87582600	-4.46135600
H	-2.36295600	7.10413500	-1.04960000
H	-6.30165200	-2.85986600	4.16045300
H	-6.27532800	-4.40339100	0.17241400
H	-6.82882800	-5.81751800	1.86303600
H	-8.66857200	-4.85956800	3.26682100
H	-7.55150500	-4.71112300	4.63218300
H	-7.95519600	-6.31170200	4.00056000
H	-4.99562900	-5.20202800	4.23989100
H	-4.47076200	-5.75854400	2.64568500
H	-5.45706400	-6.82069900	3.67674900
H	-5.65997200	0.66442200	2.99098300
H	-7.15156500	-0.93788400	5.12872200
H	-7.89253400	0.08714700	3.88911900
H	-6.85699500	0.80745300	5.13836400
H	-4.49666600	-1.29806500	5.02893000
H	-4.33064200	0.46777100	5.06503000
H	-3.59692900	-0.47194900	3.74793800
H	-3.33815900	4.40366400	1.34163900
H	-4.34590200	6.35467400	2.52514200
H	-5.37077400	5.80923500	1.18296700
H	-4.37414300	7.26325300	0.99988100
H	-1.81329700	5.93786700	2.44688200
H	-1.76952400	7.01843800	1.05378900

H	-1.04998000	5.40653700	0.93710600
H	-2.61935200	6.88078000	-4.80927700
H	-2.84611200	8.91578200	-2.52492800
H	-4.14268000	8.48507800	-3.65017800
H	-2.67780700	9.29542300	-4.24604500
H	-0.57123900	7.57512300	-2.63370300
H	-0.46055500	8.02920400	-4.34311000
H	-0.38953700	6.31933200	-3.86352200
H	-5.21406200	2.17540600	-2.34218100
H	-5.30349400	3.48197500	-5.10531400
H	-6.53923200	3.63825900	-3.84491600
H	-6.20152500	2.05497200	-4.57418300
H	-3.04336200	2.22735800	-4.49464600
H	-4.02000300	0.82303200	-4.01946900
H	-2.86956400	1.53883900	-2.87251200
H	-5.07690800	-1.20182700	-1.29340800
H	-5.78423000	-4.11544500	-1.91604200
H	-4.17182700	-3.55309300	-1.44184800
H	-4.91869100	-2.97284600	-2.94347800
H	-7.82026300	-2.51062700	-1.65822000
H	-6.99905900	-1.48199000	-2.85017700
H	-7.52993800	-0.79936000	-1.30042600

Table S9. B_6-hept [planar(S_0)] in vacuum

Energies [B3LYP-D3(BJ)/6-31G(d)]

Electronic Energy (EE)	-4584.656628	Hartree
Zero-point Energy Correction	2.388313	Hartree
Thermal Correction to Energy	2.517643	Hartree
Thermal Correction to Enthalpy	2.518587	Hartree
Thermal Correction to Free Energy	2.215209	Hartree

Single-point energy [ωB97XD/6-311G(d,p)//B3LYP-D3(BJ)/6-31G(d)]

E(RωB97XD)	-4583.697930	Hartree

Cartesian coordinates [B3LYP-D3(BJ)/6-31G(d)]

C	8.56959800	0.27046000	-2.43602400
C	8.48569500	-1.12184900	-2.47497500
C	7.34704800	-1.76306000	-1.98055200
C	7.51326600	1.01550600	-1.90507400
C	3.71405600	-0.89460100	-0.64864200
C	3.78922100	0.52457600	-0.65014500
C	2.47735900	-1.49658800	-0.37842900
C	2.62272500	1.26353900	-0.40891000
C	-1.31843100	-0.61450700	0.40296000

C	-1.25688200	0.80452000	0.35818000
C	-2.55140700	-1.22704400	0.66641900
C	-3.72819900	-0.49838500	0.88470200
C	-3.67045300	0.92023800	0.84371500
C	-2.43655700	1.53458900	0.57589900
C	6.26077800	-1.03016300	-1.47986300
C	6.34500400	0.39081400	-1.44474900
B	5.00522000	-1.74689000	-0.89368200
B	5.17338600	1.22200500	-0.83648900
C	1.37541800	0.66086400	-0.18253700
B	0.09196300	1.52708900	0.03375000
C	1.30508300	-0.75848800	-0.15600700
B	-0.05450500	-1.48187100	0.11620500
C	5.41346400	2.67688700	-0.29424500
C	0.12945800	3.09058600	-0.08933000
C	-0.15953300	-3.04690300	0.11206000
C	5.04298900	-3.25086800	-0.44002400
C	4.33383100	-4.27227100	-1.11670900
C	4.29796500	-5.55989600	-0.58157400
C	4.95751200	-5.88225000	0.60865900
C	5.68475600	-4.88001700	1.24747200
C	5.73473000	-3.57374400	0.74707100
C	0.42227800	-3.77326200	1.17216800
C	0.32408400	-5.16922100	1.19764500
C	-0.34368300	-5.86980700	0.19806100
C	-0.92865100	-5.14163500	-0.84513500
C	-0.85196200	-3.75035600	-0.91035800
C	0.72030900	3.87712900	0.92511000
C	0.67663900	5.27177900	0.83533100
C	0.08506700	5.91618800	-0.25290900
C	-0.46578200	5.12953300	-1.26627100
C	-0.46401700	3.73505600	-1.20072900
C	6.05818200	2.80681500	0.95298600
C	6.29314800	4.07776400	1.48453100
C	5.89155600	5.23511600	0.81647600
C	5.24137900	5.09452600	-0.41305300
C	4.99454500	3.83932700	-0.97449500
C	6.13235800	6.60685900	1.42220600
C	4.80543200	7.31808100	1.73712100
C	7.02422200	7.47774800	0.52198400
C	0.01072200	7.43039200	-0.33155900
C	-0.98840800	7.97967500	0.70176500
C	1.39011900	8.08937900	-0.17854300
C	1.35333900	3.21859500	2.14660100
C	0.39956700	3.23868200	3.35224000

C	2.71555400	3.82803000	2.50576600
C	-1.48485600	-2.98853600	-2.07705800
C	-2.43068300	-3.83424100	-2.93554400
C	-0.42586200	-2.31918300	-2.97270900
C	-0.45231400	-7.38286000	0.25094700
C	-1.91010600	-7.82788600	0.46046400
C	0.15697600	-8.04108400	-0.99770700
C	1.08434000	-3.04838100	2.33886000
C	2.43673400	-3.65648100	2.73192700
C	0.13433200	-2.97181000	3.54626400
C	6.51935900	-2.51565100	1.51718900
C	5.99943700	-2.33406500	2.95137200
C	8.02615800	-2.81633700	1.50234600
C	4.85784900	-7.27680500	1.20158300
C	5.36015800	-8.35592300	0.22878000
C	3.41962300	-7.57483100	1.66100200
C	3.68624800	-3.98763000	-2.46683500
C	4.70108100	-4.22498000	-3.60124200
C	2.40098600	-4.78747300	-2.71438100
C	4.32126000	3.73444300	-2.33695300
C	3.07324100	4.62200100	-2.45268900
C	5.32436800	4.03677000	-3.46406800
C	6.46357900	1.56798300	1.74430800
C	5.77919100	1.52260400	3.11928800
C	7.98924200	1.43877200	1.86173400
C	-1.02424600	2.90684000	-2.35201100
C	0.09698600	2.49364700	-3.32444500
C	-2.16892500	3.59320300	-3.10711200
C	-6.33456700	-0.39410400	1.51012700
C	-6.27537700	1.02900900	1.49774800
C	-7.53027100	-1.02314900	1.88669000
C	-8.64263900	-0.27966600	2.28982600
C	-8.58406100	1.11428900	2.28240000
C	-7.41483200	1.75826800	1.86918100
B	-4.98845800	1.75217200	0.98960400
B	-5.10404500	-1.22313600	1.03179400
C	-5.02132600	3.24074500	0.48842400
C	-5.24894400	-2.71043100	0.54289500
C	-4.81758000	-3.82224800	1.29378800
C	-4.93570900	-5.10724300	0.75449900
C	-5.46620800	-5.32651000	-0.51777300
C	-5.88442000	-4.21599300	-1.25699000
C	-5.77798600	-2.91965600	-0.74891500
C	-5.57923300	3.49831900	-0.78313000
C	-5.52970800	4.78906600	-1.31661000

C	-4.94983200	5.84825600	-0.61440600
C	-4.42770700	5.58814000	0.65383200
C	-4.44483800	4.30702700	1.21141500
C	-4.84202100	7.23744900	-1.21897200
C	-6.19497600	7.77332900	-1.71124500
C	-3.79330000	7.25697100	-2.34496600
C	-6.23098300	2.38023700	-1.59195500
C	-7.74398900	2.60748400	-1.73514600
C	-5.56204900	2.17752500	-2.95899900
C	-6.19916400	-1.73430200	-1.61111200
C	-5.38573000	-1.66158100	-2.91191400
C	-7.71083200	-1.73249900	-1.88073800
C	-5.59012700	-6.73559900	-1.07256700
C	-7.06360600	-7.12657100	-1.27687900
C	-4.78916500	-6.91652700	-2.37226700
C	-4.27292700	-3.62658200	2.70253000
C	-5.39184500	-3.79439800	3.74641500
C	-3.08391600	-4.54329700	3.02212600
C	-3.90317200	4.07176200	2.61566600
C	-2.69336300	4.95093900	2.95840300
C	-5.01754500	4.25200600	3.66339900
H	9.45998300	0.77283600	-2.80483600
H	9.31056000	-1.70618600	-2.87412300
H	7.29970000	-2.84878700	-1.97460700
H	7.59498700	2.09735400	-1.84091000
H	2.41976500	-2.57962300	-0.33631400
H	2.68566900	2.34717200	-0.39061300
H	-2.60146900	-2.31173200	0.67003600
H	-2.39644500	2.61792500	0.50559600
H	3.73867500	-6.33363300	-1.09706700
H	6.20631800	-5.12010600	2.17134200
H	0.76689100	-5.72478000	2.01937500
H	-1.45492100	-5.68250500	-1.62284100
H	1.11081800	5.86798400	1.63232200
H	-0.90537400	5.62667400	-2.12489300
H	6.78520700	4.17460000	2.44988100
H	4.91646600	5.98534600	-0.94282900
H	6.66361800	6.45556700	2.37148600
H	4.23162500	7.49981200	0.82192800
H	4.98588300	8.28627900	2.21905400
H	4.18380500	6.71025300	2.40288500
H	6.53732400	7.67764700	-0.43955200
H	7.97887300	6.98207500	0.31600100
H	7.23225200	8.44327900	0.99783900
H	-0.37156900	7.68519800	-1.32912700

H	-0.65932800	7.75016500	1.72205200
H	-1.97654300	7.52938600	0.56229100
H	-1.08659700	9.06824300	0.61342500
H	1.80870000	7.90948200	0.81768400
H	1.32003000	9.17440400	-0.31803500
H	2.09804600	7.69110600	-0.91380500
H	1.53074100	2.16531900	1.90043900
H	-0.55460700	2.75979400	3.10818100
H	0.18850400	4.26708000	3.66509500
H	0.84299100	2.70817900	4.20342400
H	2.61552300	4.86568800	2.84411200
H	3.18140500	3.26552700	3.32179000
H	3.39816800	3.81868700	1.65224400
H	-2.08901600	-2.18291400	-1.64269300
H	-1.89660400	-4.64199500	-3.45003400
H	-2.89176400	-3.20835700	-3.70591700
H	-3.23358700	-4.27211500	-2.33598500
H	0.18356800	-3.07442600	-3.47913400
H	0.25317900	-1.67503200	-2.40642700
H	-0.91134900	-1.70195000	-3.73782100
H	0.12723400	-7.72025300	1.12053900
H	-2.53969100	-7.51738100	-0.38035300
H	-2.32711200	-7.38021500	1.36875100
H	-1.97790300	-8.91876400	0.54819200
H	-0.39569700	-7.76220500	-1.90210500
H	0.13148300	-9.13379600	-0.91366100
H	1.19819300	-7.72988100	-1.13690400
H	1.27957200	-2.01642700	2.02550900
H	2.32190000	-4.66503200	3.14486900
H	2.91738600	-3.04476800	3.50336800
H	3.11256800	-3.72203800	1.87538000
H	-0.10810600	-3.97444400	3.91598500
H	-0.80494800	-2.47800400	3.27602800
H	0.59613600	-2.40843500	4.36597000
H	6.38518300	-1.55430400	1.00813800
H	6.59226000	-1.57926700	3.48001400
H	6.06158900	-3.26660300	3.52293900
H	4.95415700	-2.01114800	2.95027900
H	8.40159300	-2.87395000	0.47594000
H	8.24377200	-3.77009400	1.99694000
H	8.58231900	-2.02936000	2.02531100
H	5.50367200	-7.30185500	2.08963000
H	5.33585100	-9.34483800	0.70123600
H	4.73394800	-8.40179500	-0.66961400
H	6.38771400	-8.15146700	-0.09019100

H	2.72391600	-7.54112900	0.81528400
H	3.08315600	-6.83462200	2.39396100
H	3.35232400	-8.56996700	2.11655600
H	3.41722600	-2.92344500	-2.49128200
H	5.03030700	-5.27085200	-3.60782200
H	5.58668200	-3.59293600	-3.48121800
H	4.25287400	-4.00135400	-4.57670900
H	1.68172000	-4.66378000	-1.90006500
H	2.60608100	-5.85840800	-2.82592800
H	1.92709200	-4.45598600	-3.64436100
H	3.99232900	2.69392700	-2.46581200
H	3.33270500	5.68630100	-2.41509300
H	2.35997000	4.42431900	-1.64808900
H	2.56776300	4.44382700	-3.40748800
H	5.70759200	5.06021300	-3.37542500
H	4.84930600	3.93492100	-4.44701800
H	6.17889900	3.35283000	-3.42551500
H	6.11156000	0.68604900	1.19323400
H	6.05932200	2.38540300	3.73368800
H	6.06346900	0.61607300	3.66367500
H	4.69041000	1.52889500	3.00914400
H	8.41662700	2.29259700	2.40027700
H	8.45232000	1.39662800	0.87071000
H	8.26036900	0.52615500	2.40594400
H	-1.43229000	1.98375000	-1.92227600
H	0.52297800	3.37632500	-3.81439700
H	-0.29311600	1.82627100	-4.10233600
H	0.90828800	1.97439200	-2.80439700
H	-1.81768200	4.46664200	-3.66859500
H	-2.95965000	3.92195400	-2.42571300
H	-2.60744500	2.90063100	-3.83335200
H	-7.58893200	-2.10792400	1.85610500
H	-9.55542800	-0.78499600	2.59413700
H	-9.45107900	1.69752900	2.58116800
H	-7.38512700	2.84366300	1.82494800
H	-4.60431800	-5.96533200	1.33204300
H	-6.29031000	-4.36343400	-2.25479900
H	-5.94365100	4.96991700	-2.30519300
H	-3.98880000	6.41041100	1.21077800
H	-4.48668200	7.90914600	-0.42584700
H	-6.93815600	7.76724600	-0.90682200
H	-6.58844500	7.16664800	-2.53491400
H	-6.09257800	8.80116600	-2.07816700
H	-4.09252300	6.59738900	-3.16804200
H	-2.82367800	6.90850500	-1.97629100

H	-3.66920700	8.26908300	-2.74835500
H	-6.10468400	1.44204600	-1.03812500
H	-7.95342600	3.53354900	-2.28309700
H	-8.22142000	2.68027400	-0.75303500
H	-8.20967400	1.77810000	-2.28072500
H	-5.58911200	3.09227400	-3.56105300
H	-6.07605000	1.39201900	-3.52372500
H	-4.51463700	1.88583100	-2.84059500
H	-5.97788100	-0.81536900	-1.05350500
H	-5.52193300	-2.56282400	-3.51945400
H	-4.31768000	-1.56332300	-2.69393400
H	-5.69309500	-0.80022000	-3.51390100
H	-7.99792600	-0.85444800	-2.47162900
H	-8.27178600	-1.71052600	-0.94083000
H	-8.01481800	-2.62727400	-2.43631600
H	-5.16477000	-7.41736800	-0.32411300
H	-7.54003200	-6.48165600	-2.02452200
H	-7.62872200	-7.02898900	-0.34389000
H	-7.14792800	-8.16282300	-1.62502200
H	-5.16940000	-6.26456300	-3.16703900
H	-4.85577600	-7.95141100	-2.72781800
H	-3.73273900	-6.67231500	-2.22221300
H	-3.91340300	-2.59034400	2.77490200
H	-5.81196800	-4.80608100	3.69873300
H	-6.20536900	-3.08252900	3.57327900
H	-5.00690800	-3.63234300	4.76030900
H	-3.39441300	-5.59225400	3.09256900
H	-2.64396900	-4.27041800	3.98699100
H	-2.30411100	-4.47434000	2.25799200
H	-3.57217300	3.02523900	2.66966000
H	-2.97809100	6.00444000	3.06014000
H	-1.91550800	4.88387000	2.19180200
H	-2.26103400	4.63922600	3.91460300
H	-5.41145500	5.27478200	3.62996200
H	-4.63444000	4.06672200	4.67400700
H	-5.84901500	3.56349200	3.48396300

Table S10. B_6-hept [bent-zigzag(S_0)] in toluene

Energies [CPCM(toluene)-B3LYP-D3(BJ)/6-31G(d)]

Electronic Energy (EE)	-4584.667025	Hartree
Zero-point Energy Correction	2.382722	Hartree
Thermal Correction to Energy	2.513640	Hartree
Thermal Correction to Enthalpy	2.514584	Hartree
Thermal Correction to Free Energy	2.201086	Hartree

Single-point energy [CPCM(toluene)-ωB97XD/6-311G(d,p)//B3LYP-D3(BJ)/6-31G(d)]
E(RωB97XD) -4583.711448 Hartree

Cartesian coordinates [CPCM(toluene)-B3LYP-D3(BJ)/6-31G(d)]

C	6.42659000	-0.72947000	0.23643200
C	4.21960100	2.15514700	-3.60995100
H	4.34963000	1.32922500	-4.31904900
H	3.28022400	2.00009700	-3.06921500
H	4.12725400	3.08355600	-4.18505600
C	7.65146100	1.37383200	0.34707700
H	7.65585000	2.46055900	0.33959600
C	8.86075000	0.67715000	0.42640400
H	9.79914600	1.22010300	0.49990500
C	8.86085900	-0.71812500	0.39451700
H	9.79935200	-1.26368100	0.44346200
C	7.65181900	-1.41095900	0.28268500
H	7.65693200	-2.49620500	0.22542700
C	3.74186800	-0.73049300	0.28859700
C	3.74181700	0.68881400	0.31969900
C	2.51804800	1.36083000	0.45170900
H	2.51460600	2.44603600	0.47534000
C	1.29711400	0.68163800	0.54849200
C	1.29715600	-0.73860600	0.52011900
C	2.51956400	-1.41057900	0.39369100
H	2.51955700	-2.49582700	0.37241000
C	-1.38280000	-0.73587200	0.47293000
C	-1.38060700	0.68415100	0.49365000
C	-2.59400100	1.36635800	0.33655900
H	-2.58944400	2.45248600	0.35468200
C	-3.81187600	0.69597900	0.14304700
C	-3.81627300	-0.72336900	0.12837500
C	-2.60115700	-1.40620500	0.30187100
H	-2.60300200	-2.49238800	0.29259400
C	-6.48642000	-0.71279400	-0.21351600
C	-6.48097800	0.71195400	-0.19966100
C	-7.69955400	1.39709500	-0.31914000
H	-7.69566500	2.48348700	-0.34058300
C	-8.91147000	0.70830100	-0.42256600
H	-9.84553400	1.25754500	-0.50375900
C	-8.91652400	-0.68704900	-0.43870700
H	-9.85444300	-1.22750700	-0.53323700
C	-7.70975300	-1.38643300	-0.34925900
H	-7.71311700	-2.47214400	-0.39520600
C	5.05981300	-2.99762900	-0.40181500

C	5.22597100	-3.23979200	-1.78185500
C	5.22475600	-4.54983300	-2.26279100
H	5.35001000	-4.72606000	-3.32837500
C	5.05574600	-5.64133300	-1.40305500
C	4.86914400	-5.38742300	-0.04291700
H	4.72744200	-6.22983600	0.62992500
C	4.86094100	-4.08459900	0.46909900
C	5.35984800	-2.06687400	-2.74663400
H	5.31931400	-1.14082500	-2.15677100
C	4.17891500	-2.01012300	-3.72835400
H	4.26041500	-1.13358100	-4.38157400
H	4.14828400	-2.90311500	-4.36307600
H	3.22858400	-1.94967800	-3.18754300
C	6.71199700	-2.06585800	-3.47347200
H	7.53746200	-2.03808700	-2.75421900
H	6.83052000	-2.96340700	-4.09148900
H	6.79808100	-1.19112500	-4.12904200
C	5.07427600	-7.06600200	-1.93101900
H	4.92474200	-7.73335400	-1.07186500
C	3.92857300	-7.32213300	-2.92405700
H	2.95625900	-7.12257400	-2.46229400
H	4.01857900	-6.67635700	-3.80523000
H	3.93951400	-8.36297500	-3.26829600
C	6.43446000	-7.41308900	-2.55982200
H	6.63408000	-6.78527200	-3.43605900
H	7.24950900	-7.25834000	-1.84458000
H	6.45583000	-8.45965800	-2.88558700
C	4.67773600	-3.84543600	1.96227900
H	4.49611400	-2.77045400	2.10380000
C	3.46076100	-4.58871800	2.53345000
H	3.30634700	-4.32056400	3.58434800
H	2.54560900	-4.34761200	1.98531300
H	3.60055700	-5.67474600	2.48948500
C	5.95990500	-4.19557600	2.73672200
H	5.83745900	-3.99001100	3.80672500
H	6.20413100	-5.25813100	2.62069000
H	6.81153800	-3.61171000	2.37095700
C	5.04757000	2.99429400	-0.24005200
C	5.21524800	3.32584700	-1.59914600
C	5.15930500	4.66490800	-1.99914300
H	5.27853400	4.91816000	-3.05050900
C	4.93609800	5.69236700	-1.07993600
C	4.76369900	5.34993500	0.26532000
H	4.58788300	6.13774900	0.99236800
C	4.80827400	4.02177000	0.69530300

C	5.40448900	2.22204400	-2.63365100
H	5.42450700	1.26231600	-2.09964000
C	6.42648900	0.69463300	0.26978700
C	6.74697300	2.34103600	-3.36879800
H	7.58032900	2.31870100	-2.65843500
H	6.87548800	1.51178300	-4.07455000
H	6.80881000	3.27759600	-3.93491200
C	4.64492200	3.68778000	2.17249900
H	4.46258100	2.60644000	2.24862300
C	3.44049100	4.39486500	2.81081600
H	3.29991700	4.05428800	3.84238800
H	3.58409900	5.48100200	2.84183900
H	2.51752000	4.19556400	2.25942000
C	5.94132200	3.98593700	2.94552600
H	5.83628400	3.71468200	4.00269000
H	6.78497700	3.42426900	2.53008800
H	6.18630000	5.05328800	2.89063500
C	4.88902500	7.14378100	-1.52714500
H	4.98760500	7.15205500	-2.62077900
C	6.06887500	7.94188800	-0.94559100
H	7.02591000	7.48599900	-1.22141200
H	6.05978500	8.97443900	-1.31404700
H	6.01648300	7.97493600	0.14899100
C	3.54946600	7.81167100	-1.17400200
H	2.70938100	7.26974900	-1.62026100
H	3.39226600	7.83609800	-0.08974900
H	3.52464900	8.84517600	-1.53871500
C	-0.06939700	-3.02947400	1.07227900
C	0.01139900	-4.07727900	0.14053600
C	-0.06717600	-5.40118900	0.58807200
H	-0.00983200	-6.21595400	-0.13010100
C	-0.22314600	-5.70967700	1.94062300
C	-0.28203300	-4.65488800	2.86083000
H	-0.39060900	-4.87697100	3.91965200
C	-0.20492300	-3.32532200	2.44357200
C	0.16486400	-3.77156900	-1.34289500
H	0.22072300	-2.67787400	-1.45013900
C	-1.05742500	-4.24676300	-2.14361000
H	-0.98049600	-3.93437800	-3.19146000
H	-1.13453500	-5.33978000	-2.12642300
H	-1.98794000	-3.84459700	-1.73439400
C	1.46578900	-4.35488000	-1.91611400
H	1.58129700	-4.08186500	-2.97131600
H	2.34685400	-3.99838300	-1.37558700
H	1.46059200	-5.44880200	-1.85469100

C	-0.32728300	-7.15527600	2.39499800
H	-0.24974000	-7.78358600	1.49778800
C	-1.69072500	-7.44235800	3.04557600
H	-1.77635100	-8.50073900	3.31827600
H	-1.82672700	-6.84770100	3.95630700
H	-2.50924000	-7.19389700	2.36221000
C	0.82729300	-7.53916400	3.33503900
H	0.76423800	-8.59690400	3.61638000
H	1.79673700	-7.36665500	2.85568800
H	0.79972600	-6.94512100	4.25588800
C	-0.24022900	-2.18388900	3.45413100
H	-0.16363600	-1.24131200	2.89276000
C	-1.56606900	-2.12497300	4.22477400
H	-1.56725800	-1.28190700	4.92542200
H	-2.40845100	-2.00124500	3.53680500
H	-1.73247600	-3.04283800	4.79958400
C	0.97153600	-2.22585000	4.39724300
H	1.90630100	-2.19196800	3.82817500
H	0.95566500	-1.37249900	5.08546300
H	0.97465300	-3.14397600	4.99595600
C	-0.06698000	2.95793900	1.15139800
C	0.03277000	4.01544500	0.22954800
C	-0.03118100	5.33376900	0.68646900
H	0.04055500	6.14863400	-0.02935300
C	-0.19286900	5.62859200	2.04451700
C	-0.27805600	4.56723800	2.95071000
H	-0.39751100	4.78896000	4.00915800
C	-0.21693600	3.23796100	2.52160300
C	0.17912100	3.71668400	-1.25612400
H	0.24250900	2.62400300	-1.36859100
C	-1.05348800	4.18771700	-2.04313700
H	-1.97803100	3.77204700	-1.63218000
H	-0.98142500	3.88790400	-3.09506400
H	-1.14023200	5.27859200	-2.01125600
C	1.47023400	4.31051900	-1.83846700
H	1.45248600	5.40475800	-1.78705500
H	1.58542100	4.02894700	-2.89132900
H	2.35625000	3.96923200	-1.29676200
C	-0.28511400	7.06784700	2.52000600
H	-0.39413800	7.04520200	3.61240100
C	-1.52783700	7.76689200	1.94110300
H	-2.43975200	7.21145500	2.18493400
H	-1.62432300	8.78360700	2.33977700
H	-1.46418100	7.84007000	0.84916200
C	0.99518900	7.85509300	2.19328200

H	0.93598900	8.87617700	2.58789200
H	1.87605800	7.36835500	2.62600800
H	1.15007800	7.92293600	1.11037100
C	-0.30192100	2.08502200	3.51558400
H	-0.21068400	1.14922800	2.94481100
C	-1.66210800	2.03226300	4.22527600
H	-1.70808400	1.17602600	4.90820500
H	-1.83882600	2.94189900	4.80998700
H	-2.47472500	1.93574300	3.49814800
C	0.86498900	2.09903200	4.51387200
H	1.82451700	2.05431000	3.98894200
H	0.85559500	3.01088300	5.12195500
H	0.80264300	1.23979200	5.19208500
C	-5.04536800	-3.02879400	-0.50102600
C	-4.96848600	-3.39122800	-1.85966000
C	-4.74276000	-4.72781200	-2.20783300
H	-4.65781700	-5.00404900	-3.25669200
C	-4.59397800	-5.71951800	-1.23657700
C	-4.69217000	-5.34931200	0.10969400
H	-4.58597800	-6.11211900	0.87481500
C	-4.90481000	-4.02312700	0.49017400
C	-5.06567000	-2.31802500	-2.93932700
H	-5.32821700	-1.37235100	-2.44637200
C	-6.18638400	-2.60837600	-3.94785700
H	-7.15037900	-2.71290800	-3.43820400
H	-6.26951400	-1.79305600	-4.67600200
H	-5.99532300	-3.53411600	-4.50259700
C	-3.71559700	-2.09062600	-3.63780200
H	-2.94440900	-1.81225300	-2.91230200
H	-3.38008200	-2.99724300	-4.15389500
H	-3.79603000	-1.28641600	-4.37899900
C	-4.33813000	-7.16406900	-1.63196300
H	-4.16538500	-7.17848700	-2.71634400
C	-5.56715800	-8.04326000	-1.34217000
H	-6.45454600	-7.66133800	-1.85842500
H	-5.39508900	-9.07542600	-1.66967300
H	-5.78629700	-8.06224200	-0.26798600
C	-3.08353900	-7.73766200	-0.95215000
H	-2.20874600	-7.10686200	-1.13813200
H	-3.21566600	-7.80790800	0.13338800
H	-2.86985100	-8.74586000	-1.32550700
C	-5.02623300	-3.64032300	1.96013000
H	-4.75424800	-2.57747700	2.04310800
C	-6.48289800	-3.77841700	2.43908600
H	-6.57690100	-3.47805400	3.48947500

H	-7.15808700	-3.15537700	1.84443000
H	-6.81593300	-4.81948500	2.35082700
C	-4.07751700	-4.42374200	2.87641200
H	-4.10828500	-4.01405200	3.89178500
H	-4.36494200	-5.47898400	2.94350400
H	-3.04397400	-4.37762000	2.52174700
C	-5.03811500	3.01672300	-0.48333300
C	-4.99333300	3.37401000	-1.84684500
C	-4.84945800	4.71583200	-2.21044600
H	-4.79952700	4.97831700	-3.26413400
C	-4.75735400	5.72477200	-1.24609800
C	-4.79920900	5.35666300	0.10110200
H	-4.72331000	6.13733200	0.85329300
C	-4.92733200	4.02193400	0.49745600
C	-5.06819200	2.29037800	-2.91834400
H	-5.20572200	1.32601600	-2.41056000
C	-6.28391300	2.47679000	-3.83782200
H	-7.21191600	2.48660300	-3.25603000
H	-6.22218100	3.42037500	-4.39212500
H	-6.34473000	1.66096700	-4.56773000
C	-3.75966200	2.17980500	-3.71578900
H	-2.91784200	1.96048600	-3.05159600
H	-3.82890600	1.37688600	-4.45934500
H	-3.53626000	3.11353300	-4.24438900
C	-4.57807100	7.18417700	-1.63128800
H	-4.73189500	7.77850500	-0.72072400
C	-5.60916000	7.65092500	-2.67022300
H	-5.50036100	8.72425700	-2.86384200
H	-5.47940000	7.12854900	-3.62493200
H	-6.63134600	7.46593600	-2.32282600
C	-3.14361700	7.45256500	-2.11853200
H	-3.00272100	8.51422700	-2.35387400
H	-2.41373000	7.16928700	-1.35274900
H	-2.92392400	6.87175300	-3.02198700
C	-4.99969400	3.65178800	1.97357100
H	-4.70993700	2.59411500	2.05935200
C	-6.44223500	3.77452300	2.49687600
H	-7.12646300	3.13704700	1.92805100
H	-6.49942500	3.48149000	3.55197400
H	-6.79325700	4.80983400	2.41105400
C	-4.03158300	4.45911300	2.84895800
H	-4.03302300	4.07066200	3.87296700
H	-3.00744100	4.40942800	2.46828900
H	-4.32394800	5.51425400	2.90113300
B	5.08141400	-1.50347300	0.07972300

B	5.08123700	1.47439300	0.15291500
B	-0.04533500	-1.51766500	0.65848000
B	-0.04424900	1.45627500	0.70692100
B	-5.14243300	-1.50067000	-0.15591000
B	-5.12935900	1.48774900	-0.13618900

Table S11. B_6-hept [twist1(S_0)] in toluene

Energies[CPCM(toluene)-B3LYP-D3(BJ)/6-31G(d)]

Electronic Energy (EE)	-4584.667204	Hartree
Zero-point Energy Correction	2.383292	Hartree
Thermal Correction to Energy	2.514064	Hartree
Thermal Correction to Enthalpy	2.515008	Hartree
Thermal Correction to Free Energy	2.202958	Hartree

Single-point energy [CPCM(toluene)-ωB97XD/6-311G(d,p)//B3LYP-D3(BJ)/6-31G(d)]

E(RωB97XD)	-4583.711411	Hartree

Cartesian coordinates [CPCM(toluene)-B3LYP-D3(BJ)/6-31G(d)]

C	8.82439800	0.73504500	-1.33093900
C	8.86767500	-0.65113400	-1.17414100
C	7.70751500	-1.34758600	-0.82354800
C	7.62048700	1.41847100	-1.13782600
C	3.82040100	-0.72596900	-0.28437700
C	3.77898500	0.68381000	-0.44473200
C	2.61403500	-1.42257000	-0.11524800
C	2.53409600	1.32913500	-0.42710600
C	-1.30165400	-0.79581900	0.23031000
C	-1.30630600	0.62491400	0.21421700
C	-2.52697100	-1.46402100	0.37273000
C	-3.73118900	-0.78443400	0.60239200
C	-3.70431300	0.62925600	0.74038200
C	-2.49944200	1.30318800	0.49057000
C	6.48343600	-0.68249400	-0.65840000
C	6.43890300	0.73194600	-0.82047300
B	5.20037500	-1.45790000	-0.22764700
B	5.10437100	1.49884200	-0.57734900
C	1.33156500	0.63255100	-0.25390800
B	-0.03253300	1.38116700	-0.25943600
C	1.36752200	-0.77938000	-0.09701400
B	0.03655700	-1.58455800	0.06304600
C	5.07643600	3.05220600	-0.35296000
C	-0.16443400	2.80157500	-0.91030300
C	0.04895000	-3.15143500	0.05283900
C	5.27487900	-2.90100200	0.38367600

C	4.87784200	-4.04984000	-0.33265700
C	4.88389700	-5.29406400	0.30047300
C	5.27137200	-5.43456000	1.63792500
C	5.67138900	-4.29178900	2.33350900
C	5.67522000	-3.03011800	1.72827200
C	0.51190300	-3.87725600	1.17069100
C	0.55004700	-5.27345700	1.11770700
C	0.13770200	-5.97557100	-0.01654500
C	-0.33514300	-5.24724600	-1.11343100
C	-0.38940000	-3.85116900	-1.09374300
C	-0.03025000	3.99560400	-0.18032000
C	-0.16206200	5.22245900	-0.83631300
C	-0.43228900	5.29191700	-2.20727300
C	-0.57157900	4.09644400	-2.91834200
C	-0.43834200	2.85497000	-2.29020300
C	5.36983600	3.53638700	0.93610800
C	5.31733600	4.91101700	1.18847000
C	4.97460600	5.82358200	0.18767700
C	4.67853200	5.32852200	-1.08697100
C	4.71676100	3.96064100	-1.36755100
C	4.93755600	7.31549000	0.47394500
C	3.56855800	7.93452800	0.14693800
C	6.06260100	8.04972700	-0.27629100
C	-0.57345000	6.63175400	-2.90853900
C	-1.76673000	7.43048200	-2.35803900
C	0.72416200	7.45318200	-2.82326500
C	0.25586400	3.94303700	1.31424700
C	-0.86235000	4.60996700	2.13090500
C	1.62715100	4.54908200	1.65119500
C	-0.85278700	-3.07472800	-2.32203500
C	-2.09775800	-3.67864000	-2.98403800
C	0.29399900	-2.90287300	-3.33340400
C	0.21531300	-7.49192200	-0.05090000
C	-1.14904900	-8.13065900	-0.35577700
C	1.28405400	-7.97009200	-1.04856100
C	0.87108100	-3.13756600	2.45341800
C	2.06960000	-3.73922600	3.19640100
C	-0.35701800	-3.03768900	3.37711600
C	6.05937200	-1.79385800	2.53465100
C	5.05763000	-1.51801700	3.66668500
C	7.50193100	-1.86981200	3.05495200
C	5.23932500	-6.79209600	2.31921100
C	6.18589900	-7.79331000	1.63629500
C	3.80702700	-7.34999900	2.39069600
C	4.50392900	-3.93073200	-1.80444600

C	5.71015100	-4.27539800	-2.69639600
C	3.27766800	-4.76887100	-2.18852400
C	4.40280200	3.45013200	-2.76788200
C	3.09913900	4.03471600	-3.32961900
C	5.58308200	3.70009700	-3.72208100
C	5.69973200	2.55472500	2.05494600
C	4.64157900	2.58717900	3.16856300
C	7.11832900	2.76176500	2.60455900
C	-0.59975400	1.55891000	-3.07697000
C	0.40667700	1.44606900	-4.23097700
C	-2.04597000	1.36342000	-3.55588800
C	-6.37242500	-0.77113200	1.10177000
C	-6.31383200	0.62411600	1.38546300
C	-7.58713300	-1.45095900	1.27228600
C	-8.72578100	-0.78988700	1.74208500
C	-8.66998900	0.57660000	2.02044400
C	-7.47475100	1.27522900	1.83067100
B	-4.98853800	1.41294100	1.15929200
B	-5.11203200	-1.50842200	0.55273900
C	-4.89285200	2.96338400	1.38629900
C	-5.23890500	-2.86321800	-0.23106900
C	-4.87555800	-4.11173000	0.31353000
C	-4.99221100	-5.26557600	-0.46586100
C	-5.44695100	-5.21510200	-1.78794600
C	-5.78686800	-3.97044900	-2.32178300
C	-5.68804200	-2.79836700	-1.56617700
C	-4.92285400	3.85734500	0.29401500
C	-4.79567500	5.22890800	0.52095500
C	-4.61923100	5.74439000	1.81064600
C	-4.57451300	4.84872800	2.88017900
C	-4.70911100	3.46935200	2.68616700
C	-4.49426000	7.23994400	2.04707200
C	-5.82991100	7.95404500	1.77487100
C	-3.36183300	7.86663700	1.21825700
C	-5.14170700	3.31324500	-1.11267700
C	-6.63554600	3.34546300	-1.48209300
C	-4.28943600	4.01764500	-2.17671900
C	-5.99242300	-1.44847100	-2.20586200
C	-4.93119800	-1.08419400	-3.25679300
C	-7.41513900	-1.37483200	-2.77702400
C	-5.56257100	-6.47743400	-2.62492900
C	-4.20636700	-7.18636300	-2.77508900
C	-6.62156500	-7.43442400	-2.05148600
C	-4.40503200	-4.20595300	1.75925100
C	-5.54419900	-4.69117000	2.67279100

C	-3.15281600	-5.07999400	1.92241900
C	-4.58039500	2.50913600	3.86215100
C	-3.12511400	2.43882400	4.35516900
C	-5.55008000	2.84014900	5.00549400
H	9.72682900	1.28222700	-1.58970000
H	9.80391400	-1.18550200	-1.31088600
H	7.75171700	-2.42215700	-0.66777300
H	7.59558700	2.50112800	-1.22848400
H	2.64536400	-2.50022200	0.01592800
H	2.49999100	2.40749600	-0.54870000
H	-2.54197400	-2.54570400	0.27900300
H	-2.49710600	2.38927700	0.49285600
H	4.57695800	-6.17512200	-0.25548400
H	5.96428500	-4.38892800	3.37682300
H	0.89962200	-5.83654600	1.97810900
H	-0.65869100	-5.77970700	-2.00297400
H	-0.05291700	6.14320300	-0.26888800
H	-0.78549300	4.14214500	-3.98403500
H	5.53498100	5.28455400	2.18694100
H	4.40785100	6.02535400	-1.87540000
H	5.11397800	7.44571400	1.54995000
H	3.34048800	7.85216300	-0.92156400
H	3.55312700	8.99859000	0.41002500
H	2.76653400	7.43269900	0.69803400
H	5.93302300	7.95626500	-1.36098000
H	7.04294200	7.63424800	-0.01912200
H	6.06466400	9.11763900	-0.02798200
H	-0.76943100	6.42571300	-3.96912800
H	-2.69349400	6.85075800	-2.42836900
H	-1.90004000	8.36556800	-2.91466500
H	-1.61287700	7.68644100	-1.30404400
H	0.62583000	8.39459400	-3.37642500
H	1.57015900	6.89542200	-3.23884700
H	0.96583200	7.69922000	-1.78302500
H	0.28558900	2.88193200	1.60624300
H	-1.84361300	4.18654300	1.89845900
H	-0.90794600	5.68488000	1.92171700
H	-0.68393300	4.48706200	3.20533800
H	1.64902300	5.61686800	1.40663500
H	1.84603300	4.44640400	2.72032500
H	2.43343500	4.06726900	1.09146900
H	-1.13221200	-2.06793600	-1.98685400
H	-1.88083300	-4.65302100	-3.43693400
H	-2.45965100	-3.02127900	-3.78211300
H	-2.90839200	-3.81371900	-2.26258900

H	0.61455700	-3.87462000	-3.72579800
H	1.16245700	-2.42557400	-2.86709000
H	-0.02719900	-2.28140400	-4.17833500
H	0.52394600	-7.82350300	0.94936600
H	-1.49319000	-7.86625300	-1.36155400
H	-1.90944900	-7.79263400	0.35675400
H	-1.08621900	-9.22368900	-0.30162800
H	1.03425700	-7.65784900	-2.06931800
H	1.36589800	-9.06334300	-1.03895700
H	2.26443100	-7.54853600	-0.80272700
H	1.14794500	-2.11202600	2.17545400
H	2.93827900	-3.84326100	2.54072700
H	1.83320000	-4.72769300	3.60693000
H	2.35302600	-3.09861600	4.03831800
H	-0.67680300	-4.03537400	3.69877900
H	-1.20061900	-2.56298900	2.86520400
H	-0.12152000	-2.44905500	4.27183000
H	6.01623200	-0.92815800	1.86118700
H	5.33316700	-0.60662100	4.21034600
H	5.03052000	-2.34583400	4.38428300
H	4.04701700	-1.38701700	3.26673200
H	8.20623200	-1.99883900	2.22613100
H	7.63326400	-2.71091400	3.74540100
H	7.76710300	-0.95043100	3.59034600
H	5.59206800	-6.64873400	3.34917500
H	6.19202700	-8.74905400	2.17322600
H	5.87200000	-7.99146800	0.60486100
H	7.21103400	-7.40854400	1.60650000
H	3.78989600	-8.30854400	2.92248900
H	3.39736900	-7.51518900	1.38764600
H	3.14127600	-6.65440800	2.91108100
H	4.24994900	-2.87843600	-1.99519400
H	6.02457900	-5.31299200	-2.53245600
H	6.56312500	-3.62496400	-2.47591600
H	5.45622000	-4.15933300	-3.75672600
H	2.42456300	-4.55941800	-1.53712500
H	3.48982400	-5.84202400	-2.12912000
H	2.97952000	-4.55281600	-3.21999100
H	4.26474100	2.36119400	-2.69806900
H	2.25871700	3.87061700	-2.64985000
H	2.85368100	3.57100200	-4.29139800
H	3.18705800	5.11391500	-3.49916800
H	5.78215100	4.77426700	-3.81582200
H	5.36648100	3.30585600	-4.72199200
H	6.49549000	3.21855000	-3.35454100

H	5.67356200	1.54185800	1.62881900
H	4.60007400	3.57230600	3.64706400
H	4.87122800	1.84451600	3.94149500
H	3.64766000	2.36762900	2.76507400
H	7.22775100	3.75456900	3.05617500
H	7.86042400	2.67014600	1.80413900
H	7.34810500	2.01433100	3.37331600
H	-0.38097700	0.72512900	-2.39550400
H	0.26046300	2.24365900	-4.96829800
H	0.29309700	0.48523700	-4.74666500
H	1.43339100	1.51666400	-3.85775000
H	-2.34520300	2.16243200	-4.24384300
H	-2.73933700	1.37137300	-2.70882100
H	-2.15501700	0.40593800	-4.07836300
H	-7.64098000	-2.50898800	1.03005100
H	-9.65520500	-1.33592500	1.87943800
H	-9.55567900	1.09691400	2.37476300
H	-7.43776400	2.34303900	2.02965400
H	-4.72030000	-6.22602600	-0.03720200
H	-6.12018600	-3.91738000	-3.35592900
H	-4.82820800	5.91365100	-0.32124300
H	-4.41658500	5.23891800	3.88318500
H	-4.25016000	7.38224200	3.10821800
H	-6.63249300	7.53541600	2.39167000
H	-6.12240600	7.84382000	0.72391900
H	-5.75087900	9.02577900	1.99241000
H	-3.56872800	7.79272100	0.14527700
H	-2.40795100	7.36366700	1.40830300
H	-3.24680500	8.92906900	1.46242400
H	-4.83408900	2.25751500	-1.10573300
H	-7.00425100	4.37802400	-1.50240600
H	-7.23314500	2.78692900	-0.75401400
H	-6.80087400	2.90404900	-2.47244000
H	-4.60304100	5.05843000	-2.31878300
H	-4.39413500	3.51102600	-3.14231800
H	-3.22926400	4.01670900	-1.90681800
H	-5.93159600	-0.68288900	-1.42225000
H	-4.93381500	-1.80117000	-4.08599600
H	-3.93081700	-1.08749000	-2.81259500
H	-5.11903500	-0.08550800	-3.66861200
H	-7.61463800	-0.37661300	-3.18388000
H	-8.15627400	-1.58189100	-1.99742600
H	-7.56157500	-2.10079600	-3.58492000
H	-5.89410400	-6.17467800	-3.62702100
H	-3.45503600	-6.51288400	-3.20043700

H	-4.29652300	-8.06156500	-3.42908500
H	-3.83341600	-7.53124800	-1.80456500
H	-6.33771200	-7.77714400	-1.04953600
H	-6.73298100	-8.31877900	-2.68981300
H	-7.59647300	-6.94118500	-1.97318400
H	-4.13724600	-3.19087000	2.08605200
H	-5.86882900	-5.69735400	2.38220600
H	-6.41144200	-4.02518400	2.60832100
H	-5.21708000	-4.72748300	3.71865900
H	-3.36646500	-6.13083800	1.69577700
H	-2.78976400	-5.03525100	2.95465400
H	-2.34286300	-4.75431900	1.26351300
H	-4.84561600	1.50537800	3.50184100
H	-2.78959700	3.41551900	4.72242500
H	-2.45406000	2.13739100	3.54390200
H	-3.02570000	1.71468600	5.17241800
H	-5.33648700	3.82314500	5.44030500
H	-5.46781400	2.09717500	5.80705900
H	-6.58612200	2.84701800	4.64963500

Table S12. B_6-hept in toluene [optimized S_1 geometry starting from the bent-zigzag(S_0) structure]

Energies [CPCM(toluene)-TD-CAM-B3LYP-D3(BJ)/6-31G(d)]

Electronic Energy (EE)	-4581.409373	Hartree
Zero-point Energy Correction	2.402747	Hartree
Thermal Correction to Energy	2.532913	Hartree
Thermal Correction to Enthalpy	2.533857	Hartree
Thermal Correction to Free Energy	2.221654	Hartree

Single-point energy [CPCM(toluene)-TD-ωB97XD/6-311G(d,p)//B3LYP-D3(BJ)/6-31G(d)]

E(RωB97XD)	-4583.594170	Hartree

Cartesian coordinates [CPCM(toluene)-TD-CAM-B3LYP-D3(BJ)/6-31G(d)]

C	6.40900	-0.63770	0.25550
C	4.12940	2.22520	-3.55620
H	4.26280	1.41040	-4.27570
H	3.20670	2.04020	-2.99840
H	4.00180	3.15540	-4.11990
C	7.60450	1.47080	0.38160
H	7.59710	2.55700	0.37680
C	8.81700	0.79340	0.46560
H	9.74700	1.34820	0.54530
C	8.83440	-0.59680	0.42970
H	9.77820	-1.13130	0.48330

C	7.63990	-1.30030	0.30870
H	7.66030	-2.38470	0.25020
C	3.73530	-0.67950	0.25810
C	3.71820	0.74530	0.30390
C	2.49870	1.39810	0.41350
H	2.48050	2.48380	0.43150
C	1.27460	0.70270	0.46460
C	1.28820	-0.71070	0.44280
C	2.52660	-1.36550	0.32020
H	2.53410	-2.45070	0.28580
C	-1.33990	-0.74350	0.37200
C	-1.36060	0.67070	0.38590
C	-2.59840	1.33490	0.27350
H	-2.60760	2.42130	0.28680
C	-3.79620	0.65230	0.11030
C	-3.77910	-0.77340	0.08120
C	-2.55710	-1.42880	0.20640
H	-2.53710	-2.51460	0.18790
C	-6.44430	-0.79310	-0.17700
C	-6.46040	0.62470	-0.15550
C	-7.68880	1.28950	-0.24440
H	-7.70300	2.37550	-0.25620
C	-8.88680	0.58780	-0.33250
H	-9.82910	1.12380	-0.39410
C	-8.87090	-0.80260	-0.35810
H	-9.80110	-1.35700	-0.43890
C	-7.65780	-1.48040	-0.29350
H	-7.64780	-2.56550	-0.34130
C	5.09900	-2.94080	-0.36580
C	5.26570	-3.19640	-1.73940
C	5.30580	-4.50750	-2.20150
H	5.42820	-4.69270	-3.26560
C	5.18600	-5.58920	-1.33000
C	5.00500	-5.32420	0.02300
H	4.90340	-6.16200	0.70810
C	4.95150	-4.02040	0.51610
C	5.36610	-2.04070	-2.72480
H	5.33720	-1.10630	-2.15160
C	4.16450	-2.00750	-3.67360
H	4.23100	-1.14990	-4.35160
H	4.11510	-2.91600	-4.28310
H	3.22870	-1.92860	-3.11250
C	6.69290	-2.04590	-3.48700
H	7.53890	-2.00850	-2.79420
H	6.79750	-2.94720	-4.10000

H	6.75810	-1.17870	-4.15300
C	5.23930	-7.01680	-1.83810
H	5.18230	-7.67450	-0.96210
C	4.04130	-7.33850	-2.73780
H	3.09820	-7.18300	-2.20610
H	4.03330	-6.69730	-3.62570
H	4.07710	-8.37960	-3.07560
C	6.55940	-7.31420	-2.55660
H	6.66580	-6.70140	-3.45800
H	7.41650	-7.10700	-1.90880
H	6.60580	-8.36480	-2.86150
C	4.76800	-3.77600	2.00520
H	4.53400	-2.71160	2.13680
C	3.59660	-4.57050	2.58750
H	3.43110	-4.29810	3.63460
H	2.67000	-4.38110	2.03900
H	3.78970	-5.64770	2.55700
C	6.06310	-4.05930	2.77440
H	5.93450	-3.85360	3.84230
H	6.35680	-5.10890	2.66510
H	6.88550	-3.43940	2.40480
C	5.00460	3.07430	-0.21220
C	5.14980	3.40580	-1.56930
C	5.09390	4.74060	-1.96700
H	5.19600	4.99280	-3.01990
C	4.89730	5.76600	-1.04790
C	4.75440	5.42550	0.29590
H	4.60350	6.21500	1.02640
C	4.79740	4.10160	0.72380
C	5.33000	2.30940	-2.60950
H	5.37970	1.34990	-2.08130
C	6.39160	0.77950	0.29230
C	6.64750	2.45380	-3.37460
H	7.49840	2.45110	-2.68680
H	6.77560	1.62520	-4.07930
H	6.67750	3.38720	-3.94630
C	4.65830	3.77470	2.20230
H	4.43940	2.70220	2.28290
C	3.49890	4.52170	2.86550
H	3.35720	4.17440	3.89340
H	3.68750	5.59960	2.90870
H	2.56280	4.36690	2.32260
C	5.97280	4.03290	2.94750
H	5.87760	3.77280	4.00700
H	6.78970	3.44130	2.52440

H	6.25240	5.09000	2.88140
C	4.84930	7.21410	-1.49470
H	4.90970	7.21860	-2.58980
C	6.05130	7.99940	-0.95790
H	6.99340	7.53700	-1.26700
H	6.03820	9.03090	-1.32530
H	6.03870	8.03300	0.13680
C	3.53450	7.89420	-1.09850
H	2.67290	7.35490	-1.50330
H	3.42170	7.93450	-0.01010
H	3.50220	8.92200	-1.47440
C	-0.02340	-3.04090	0.95130
C	0.04680	-4.09220	0.01890
C	-0.01530	-5.41000	0.46310
H	0.03460	-6.22310	-0.25650
C	-0.14280	-5.72240	1.81380
C	-0.19010	-4.67340	2.73600
H	-0.27530	-4.90160	3.79520
C	-0.12970	-3.34840	2.32700
C	0.18490	-3.79910	-1.46390
H	0.20960	-2.70850	-1.58150
C	-1.01790	-4.32180	-2.25450
H	-0.95170	-4.01560	-3.30370
H	-1.05830	-5.41590	-2.23050
H	-1.96070	-3.94690	-1.84920
C	1.49530	-4.35640	-2.02840
H	1.60690	-4.08470	-3.08320
H	2.36700	-3.97780	-1.48850
H	1.51440	-5.44910	-1.96400
C	-0.21530	-7.16580	2.26540
H	-0.19850	-7.78850	1.36280
C	-1.52220	-7.46110	3.00870
H	-1.58340	-8.52160	3.27350
H	-1.59240	-6.88040	3.93440
H	-2.39030	-7.20940	2.39270
C	1.00140	-7.54510	3.11700
H	0.96090	-8.60190	3.40030
H	1.93300	-7.37080	2.57070
H	1.03780	-6.95210	4.03700
C	-0.16210	-2.22810	3.35620
H	-0.11170	-1.27840	2.81270
C	-1.46930	-2.20980	4.15130
H	-1.47180	-1.37800	4.86390
H	-2.32800	-2.09060	3.48430
H	-1.60850	-3.13670	4.71700

C	1.06160	-2.27210	4.27540
H	1.98600	-2.20240	3.69480
H	1.03910	-1.43750	4.98440
H	1.09540	-3.20260	4.85200
C	-0.09310	2.96270	0.99210
C	-0.04540	4.05640	0.08090
C	-0.13210	5.34510	0.56260
H	-0.10090	6.17960	-0.13060
C	-0.26520	5.60750	1.93690
C	-0.29330	4.53430	2.83340
H	-0.38800	4.74610	3.89430
C	-0.20420	3.22560	2.39410
C	0.09140	3.79710	-1.40440
H	0.12270	2.71020	-1.54380
C	-1.11760	4.33440	-2.17600
H	-2.05860	3.94980	-1.77460
H	-1.05410	4.04630	-3.22980
H	-1.15860	5.42710	-2.13170
C	1.39900	4.37730	-1.95230
H	1.40880	5.46880	-1.86620
H	1.51220	4.12570	-3.01110
H	2.27170	3.99360	-1.41890
C	-0.37710	7.02670	2.43370
H	-0.46690	6.98680	3.52480
C	-1.63900	7.70360	1.88050
H	-2.53620	7.12600	2.12080
H	-1.74810	8.70470	2.30820
H	-1.58800	7.80850	0.79230
C	0.88210	7.83390	2.09120
H	0.80950	8.84220	2.50970
H	1.77960	7.35680	2.49590
H	1.01110	7.92860	1.00850
C	-0.22770	2.07830	3.38590
H	-0.13790	1.14870	2.81810
C	-1.55790	2.01220	4.14220
H	-1.56020	1.15380	4.82120
H	-1.72880	2.91450	4.73790
H	-2.39530	1.89930	3.44790
C	0.96910	2.13360	4.34030
H	1.91110	2.10090	3.78580
H	0.96210	3.04590	4.94560
H	0.94460	1.27700	5.02120
C	-5.00460	-3.10890	-0.45570
C	-4.97020	-3.49790	-1.80410
C	-4.77230	-4.83900	-2.13310

H	-4.72330	-5.13480	-3.17850
C	-4.61130	-5.81170	-1.15350
C	-4.66930	-5.41720	0.18300
H	-4.55310	-6.16830	0.95790
C	-4.85660	-4.08750	0.54480
C	-5.10150	-2.45620	-2.90690
H	-5.33450	-1.49540	-2.43340
C	-6.26230	-2.76680	-3.85500
H	-7.20440	-2.84840	-3.30430
H	-6.37100	-1.97350	-4.60190
H	-6.10200	-3.70850	-4.39000
C	-3.78710	-2.26360	-3.66880
H	-2.98340	-1.96470	-2.98980
H	-3.47880	-3.18790	-4.16850
H	-3.89610	-1.48590	-4.43270
C	-4.37860	-7.26220	-1.52780
H	-4.29160	-7.30500	-2.62050
C	-5.56580	-8.14120	-1.11930
H	-6.49570	-7.78220	-1.57050
H	-5.40930	-9.17830	-1.43440
H	-5.69840	-8.13750	-0.03210
C	-3.07210	-7.80220	-0.93610
H	-2.22080	-7.17780	-1.22160
H	-3.11260	-7.82670	0.15780
H	-2.88310	-8.82310	-1.28370
C	-4.94130	-3.68700	2.00990
H	-4.62090	-2.63880	2.07820
C	-6.38990	-3.75950	2.50980
H	-6.45450	-3.44790	3.55790
H	-7.04890	-3.11440	1.92300
H	-6.76760	-4.78530	2.43670
C	-4.02240	-4.50150	2.92070
H	-4.01910	-4.07760	3.92960
H	-4.35930	-5.53970	3.00790
H	-2.99330	-4.50920	2.55240
C	-5.08120	2.97060	-0.41420
C	-5.06240	3.36560	-1.76390
C	-4.96310	4.71530	-2.09120
H	-4.93370	5.00720	-3.13780
C	-4.89360	5.69810	-1.10570
C	-4.91830	5.29540	0.22590
H	-4.86800	6.05830	0.99780
C	-5.00200	3.95160	0.58680
C	-5.12420	2.31710	-2.86660
H	-5.27340	1.34000	-2.39230

C	-6.31940	2.53590	-3.79740
H	-7.25680	2.53990	-3.23290
H	-6.24260	3.48920	-4.33060
H	-6.37470	1.73830	-4.54560
C	-3.80920	2.22820	-3.64580
H	-2.97730	1.98430	-2.97890
H	-3.86970	1.44950	-4.41370
H	-3.57650	3.17600	-4.14270
C	-4.77190	7.16760	-1.45960
H	-4.85630	7.73250	-0.52310
C	-5.89800	7.63460	-2.38650
H	-5.82370	8.71110	-2.57160
H	-5.85060	7.12790	-3.35600
H	-6.87960	7.42890	-1.94940
C	-3.39890	7.48050	-2.06420
H	-3.30030	8.55000	-2.27750
H	-2.59640	7.19130	-1.37880
H	-3.24930	6.93390	-3.00140
C	-5.05330	3.54970	2.05270
H	-4.73100	2.50190	2.11420
C	-6.48770	3.62280	2.59030
H	-7.15980	2.97660	2.01960
H	-6.52580	3.31080	3.63950
H	-6.86910	4.64790	2.52680
C	-4.10680	4.36560	2.93530
H	-4.07890	3.95020	3.94720
H	-3.08810	4.36230	2.53870
H	-4.43420	5.40700	3.02120
B	5.07610	-1.43690	0.09260
B	5.03960	1.54860	0.16840
B	-0.01930	-1.52910	0.55260
B	-0.05690	1.48260	0.54380
B	-5.08940	-1.57040	-0.14140
B	-5.12260	1.42750	-0.11030

Table S13. B_6-hept in toluene [optimized S_1 geometry starting from the twist1(S_0) structure]

Energies [CPCM(toluene)-TD-CAM-B3LYP-D3(BJ)/6-31G(d)]

Electronic Energy (EE)	-4581.409686	Hartree
Zero-point Energy Correction	2.403935	Hartree
Thermal Correction to Energy	2.533791	Hartree
Thermal Correction to Enthalpy	2.534736	Hartree
Thermal Correction to Free Energy	2.226248	Hartree

Single-point energy [CPCM(toluene)-TD-ωB97XD/6-311G(d,p)//B3LYP-D3(BJ)/6-31G(d)]

E(RωB97XD) -4583.594082 Hartree

Cartesian coordinates [CPCM(toluene)-TD-CAM-B3LYP-D3(BJ)/6-31G(d)]

C	8.78940	0.63020	-1.32260
C	8.81370	-0.75040	-1.15660
C	7.64730	-1.42560	-0.81010
C	7.59800	1.32580	-1.14160
C	3.78400	-0.75730	-0.27180
C	3.76000	0.65820	-0.44640
C	2.57870	-1.42630	-0.08490
C	2.53810	1.31430	-0.41680
C	-1.28740	-0.76680	0.24120
C	-1.28850	0.65090	0.21570
C	-2.52770	-1.42090	0.36990
C	-3.72730	-0.74190	0.54390
C	-3.69720	0.67970	0.66700
C	-2.49570	1.33820	0.44630
C	6.43370	-0.74760	-0.65560
C	6.40900	0.66000	-0.82500
B	5.13740	-1.51100	-0.23220
B	5.08470	1.45130	-0.59340
C	1.31880	0.63520	-0.22110
B	-0.00460	1.42020	-0.15540
C	1.33020	-0.77510	-0.07930
B	0.01790	-1.57860	0.08200
C	5.09420	3.01030	-0.38370
C	-0.11860	2.83210	-0.77650
C	0.01770	-3.14920	0.08730
C	5.20210	-2.96400	0.36500
C	4.83600	-4.11080	-0.36110
C	4.83750	-5.35310	0.26580
C	5.19140	-5.49570	1.60680
C	5.56050	-4.35590	2.31280
C	5.56870	-3.09680	1.71400
C	0.44760	-3.86870	1.22290
C	0.48780	-5.25940	1.18400
C	0.10160	-5.97170	0.05300
C	-0.34860	-5.25730	-1.05720
C	-0.39620	-3.86760	-1.05860
C	0.02270	4.04780	-0.03880
C	-0.09390	5.25230	-0.69200
C	-0.35620	5.31770	-2.07600
C	-0.50400	4.12900	-2.79940
C	-0.39180	2.89600	-2.18830

C	5.38890	3.49060	0.90220
C	5.37530	4.86240	1.14890
C	5.07540	5.77910	0.14640
C	4.78370	5.29060	-1.12590
C	4.78220	3.92590	-1.40150
C	5.07890	7.26860	0.43030
C	3.73570	7.92270	0.09070
C	6.22610	7.96960	-0.30600
C	-0.47700	6.65090	-2.76520
C	-1.66400	7.44930	-2.20910
C	0.82880	7.44980	-2.65340
C	0.30430	3.99650	1.44710
C	-0.80050	4.69130	2.24970
C	1.68130	4.58840	1.76720
C	-0.84850	-3.11550	-2.30290
C	-1.95690	-3.82710	-3.07900
C	0.34130	-2.79810	-3.21780
C	0.17470	-7.48430	0.03680
C	-1.19790	-8.11620	-0.21510
C	1.20230	-7.98150	-0.98590
C	0.79810	-3.12860	2.50490
C	1.90600	-3.79530	3.32040
C	-0.45660	-2.92590	3.36520
C	5.92680	-1.86790	2.53800
C	4.87100	-1.58000	3.60900
C	7.33100	-1.96370	3.13870
C	5.15300	-6.85090	2.28550
C	6.07140	-7.86360	1.59440
C	3.71920	-7.38590	2.37790
C	4.48750	-3.99370	-1.83650
C	5.70990	-4.30910	-2.70730
C	3.29280	-4.85590	-2.24590
C	4.47340	3.42970	-2.80530
C	3.22040	4.07990	-3.39590
C	5.67750	3.61760	-3.73470
C	5.69380	2.51510	2.03010
C	4.63200	2.57030	3.13190
C	7.10440	2.71200	2.59030
C	-0.55870	1.62450	-2.99530
C	0.49500	1.51670	-4.10220
C	-1.98390	1.50140	-3.54590
C	-6.37120	-0.70470	0.94710
C	-6.31540	0.68590	1.22200
C	-7.59160	-1.37350	1.08300
C	-8.73800	-0.70580	1.50380

C	-8.68410	0.65720	1.77480
C	-7.48310	1.34330	1.62480
B	-4.97720	1.46800	1.04760
B	-5.09320	-1.46360	0.46320
C	-4.88850	3.01790	1.30400
C	-5.21160	-2.84740	-0.27660
C	-4.92380	-4.08370	0.32350
C	-5.04330	-5.25600	-0.41920
C	-5.42940	-5.23810	-1.75790
C	-5.69460	-4.00630	-2.34690
C	-5.59020	-2.81690	-1.62930
C	-4.95680	3.93910	0.24430
C	-4.83990	5.30100	0.50500
C	-4.64030	5.78170	1.79890
C	-4.56470	4.86050	2.83760
C	-4.68780	3.49020	2.60880
C	-4.52170	7.26810	2.07320
C	-5.85170	7.98610	1.81810
C	-3.39200	7.91440	1.26580
C	-5.19700	3.44120	-1.17280
C	-6.68010	3.54740	-1.54570
C	-4.31730	4.14090	-2.21080
C	-5.82910	-1.48770	-2.33110
C	-4.72440	-1.19600	-3.35130
C	-7.22120	-1.40130	-2.96020
C	-5.55350	-6.52080	-2.55620
C	-4.22510	-7.28110	-2.61850
C	-6.66930	-7.41590	-2.00630
C	-4.51660	-4.14940	1.78690
C	-5.69260	-4.59640	2.66270
C	-3.29060	-5.03650	2.01900
C	-4.55670	2.50800	3.76280
C	-3.11680	2.45340	4.28350
C	-5.55120	2.79790	4.88940
H	9.69930	1.16450	-1.57900
H	9.74280	-1.29800	-1.28390
H	7.67790	-2.49930	-0.64850
H	7.58950	2.40770	-1.23960
H	2.59340	-2.50270	0.06120
H	2.52090	2.39560	-0.52210
H	-2.53990	-2.50480	0.29490
H	-2.48300	2.42470	0.46330
H	4.55130	-6.23430	-0.30040
H	5.83040	-4.45430	3.36180
H	0.82300	-5.81450	2.05410

H	-0.66160	-5.80240	-1.94180
H	0.01680	6.17790	-0.13690
H	-0.70840	4.19190	-3.86360
H	5.59540	5.23160	2.14800
H	4.55040	5.99350	-1.92080
H	5.24740	7.39350	1.50680
H	3.52300	7.85550	-0.98110
H	3.74230	8.98340	0.36200
H	2.91320	7.43780	0.62540
H	6.10620	7.87910	-1.39100
H	7.19170	7.53020	-0.03890
H	6.25400	9.03610	-0.05910
H	-0.66600	6.45650	-3.82630
H	-2.59580	6.88180	-2.28780
H	-1.78040	8.38310	-2.76690
H	-1.51220	7.70350	-1.15610
H	0.74300	8.38560	-3.21330
H	1.67510	6.88480	-3.05400
H	1.05370	7.70110	-1.61260
H	0.31700	2.93960	1.73780
H	-1.78850	4.28430	2.02040
H	-0.82340	5.76600	2.03970
H	-0.62340	4.56630	3.32210
H	1.71330	5.65670	1.52900
H	1.90350	4.47710	2.83270
H	2.47770	4.09770	1.20270
H	-1.25950	-2.15750	-1.96860
H	-1.59690	-4.74660	-3.55310
H	-2.33200	-3.17820	-3.87630
H	-2.79900	-4.08390	-2.43060
H	0.78210	-3.71920	-3.61250
H	1.12320	-2.25370	-2.68020
H	0.01920	-2.18450	-4.06700
H	0.51320	-7.80260	1.03030
H	-1.57180	-7.85980	-1.21100
H	-1.93290	-7.76780	0.51710
H	-1.13920	-9.20780	-0.15150
H	0.91510	-7.69380	-2.00280
H	1.28400	-9.07300	-0.95520
H	2.19090	-7.55670	-0.78820
H	1.15950	-2.13470	2.22030
H	2.79500	-3.98650	2.71340
H	1.57550	-4.74640	3.75160
H	2.19810	-3.14870	4.15320
H	-0.85910	-3.89120	3.68980

H	-1.24050	-2.40310	2.81010
H	-0.21990	-2.33810	4.25870
H	5.93510	-1.00320	1.86500
H	5.12590	-0.67240	4.16690
H	4.79450	-2.40590	4.32370
H	3.88620	-1.43720	3.15440
H	8.08010	-2.11210	2.35500
H	7.40980	-2.79780	3.84360
H	7.58070	-1.04470	3.67970
H	5.52170	-6.71130	3.30910
H	6.07370	-8.81560	2.13530
H	5.73900	-8.06420	0.57050
H	7.10030	-7.49450	1.54570
H	3.69470	-8.34360	2.90850
H	3.29410	-7.54380	1.38110
H	3.07110	-6.68220	2.90780
H	4.21070	-2.94850	-2.02490
H	6.04430	-5.33900	-2.54100
H	6.54530	-3.64210	-2.47610
H	5.46960	-4.19770	-3.77010
H	2.42320	-4.66850	-1.61060
H	3.52750	-5.92370	-2.18930
H	3.01030	-4.64150	-3.28120
H	4.27470	2.35240	-2.73640
H	2.36630	3.97950	-2.72120
H	2.95750	3.61240	-4.34990
H	3.37480	5.14730	-3.58640
H	5.93560	4.67860	-3.82250
H	5.45820	3.23770	-4.73820
H	6.55560	3.08750	-3.35510
H	5.66010	1.50050	1.61510
H	4.58880	3.56230	3.59390
H	4.85510	1.84180	3.91840
H	3.64090	2.34290	2.72840
H	7.22000	3.70400	3.03960
H	7.85390	2.60960	1.79980
H	7.31920	1.96650	3.36330
H	-0.40210	0.77750	-2.32450
H	0.40720	2.33070	-4.82910
H	0.37340	0.57070	-4.63860
H	1.50440	1.54200	-3.68210
H	-2.22360	2.31960	-4.23290
H	-2.71740	1.51060	-2.73470
H	-2.09290	0.55860	-4.09010
H	-7.64370	-2.43330	0.85070

H	-9.67400	-1.24580	1.61170
H	-9.57710	1.18470	2.09650
H	-7.44660	2.41040	1.82550
H	-4.82740	-6.20900	0.05530
H	-5.97730	-3.97840	-3.39660
H	-4.90350	6.00820	-0.31700
H	-4.39680	5.22440	3.84840
H	-4.27910	7.38290	3.13670
H	-6.65490	7.55280	2.42150
H	-6.14320	7.90420	0.76540
H	-5.77390	9.05050	2.06330
H	-3.59760	7.86670	0.19170
H	-2.43830	7.40900	1.44610
H	-3.27790	8.96950	1.53520
H	-4.92970	2.37660	-1.19490
H	-7.00720	4.59290	-1.53100
H	-7.30350	2.98870	-0.84180
H	-6.85860	3.14930	-2.55040
H	-4.59210	5.19460	-2.32750
H	-4.42940	3.66430	-3.18970
H	-3.26140	4.09600	-1.93010
H	-5.77760	-0.69290	-1.57850
H	-4.71250	-1.94780	-4.14760
H	-3.74210	-1.20150	-2.86920
H	-4.87280	-0.21410	-3.81390
H	-7.37810	-0.41670	-3.41300
H	-7.99810	-1.55760	-2.20560
H	-7.35540	-2.15380	-3.74400
H	-5.82590	-6.23960	-3.58090
H	-3.42730	-6.65020	-3.02200
H	-4.31560	-8.16960	-3.25210
H	-3.91590	-7.61260	-1.62230
H	-6.44660	-7.73410	-0.98230
H	-6.78360	-8.31620	-2.61910
H	-7.62730	-6.88740	-1.99110
H	-4.24350	-3.13280	2.09830
H	-6.02120	-5.60350	2.38360
H	-6.54660	-3.92140	2.55200
H	-5.40690	-4.61410	3.71980
H	-3.51090	-6.08800	1.80670
H	-2.96940	-4.97510	3.06330
H	-2.44960	-4.73700	1.38780
H	-4.79610	1.50860	3.37850
H	-2.80350	3.42720	4.67460
H	-2.42390	2.17240	3.48460

H	-3.02410	1.71890	5.09030
H	-5.36440	3.77440	5.34790
H	-5.47130	2.04110	5.67630
H	-6.57930	2.79310	4.51480
